# Supplementary material for: Do care plans and annual reviews of physical health influence unplanned hospital utilisation for people with serious mental illness? Analysis of linked longitudinal primary and secondary healthcare records in England
Source: BMJ Open. 2018 Nov 28;8(11):e023135. doi: 10.1136/bmjopen-2018-023135 (PMC6278786; doi:10.1136/bmjopen-2018-023135)
Supplement: Supplementary data [file bmjopen-2018-023135supp001.pdf]

Supplementary Table 1: Conditions classified as ambulatory care sensitive admissions[^]

Condition	ICD-10 codes used to identify the condition[#]
Angina	I10 I24.0 I24.8 I24.9
Asthma	J45 J46
Chronic obstructive pulmonary disease	J41 J42 J43 J44 J47<br>First diagnosis: J20 + Second diagnosis one of: J41 J42 J43 J44 J47
Congestive heart failure	I50 I11.0 J81
Diabetes (in any diagnosis field)	E10.0 E10.1 E10.2 E10.3 E10.4 E10.5 E10.6 E10.7 E10.8<br>E11.0 E10.1 E11.2 E10.3 E11.4 E10.5 E11.6 E10.7 E11.8<br>E12.0 E10.1 E12.2 E10.3 E12.4 E10.5 E12.6 E10.7 E12.8<br>E13.0 E10.1 E13.2 E10.3 E13.4 E10.5 E13.6 E10.7 E13.8<br>E14.0 E10.1 E14.2 E10.3 E14.4 E10.5 E14.6 E10.7 E14.8
Epilepsy	G40 G41 O15 R56
Hypertension	I10 I11.9
Anaemia	D50.0 D50.8 D50.9
Cellulitis	L03 L04 L08 L88 L98.0 L98.3
Dehydration	E86
Dental	A69.0 K02 K03 K04 K05 K06 K08 K09.8 K09.9 K12 K13
Ear, nose and throat infections	H66 H67 J02 J03 J06 J31.2
Gangrene (in any diagnosis field)	R02
Gastroenteritis	K52.2 K52.8 K52.9

Nutritional deficiencies	E40 E41 E42 E43 E55 E64.3
Perforated or bleeding ulcer	K25.0 K25.1 K25.2 K25.4 K25.5 K25.6<br>K26.0 K26.1 K26.2 K26.4 K26.5 K26.6<br>K27.0 K27.1 K27.2 K27.4 K27.5 K27.6<br>K28.0 K28.1 K28.2 K28.4 K28.5 K28.6
Urinary tract infection or pyelonephritis	N10 N11 N12 N13.6 N39.0
Influenza (in any diagnosis field, exclude secondary diagnosis of D57)	J10 J11
Pneumonia (in any diagnosis field, exclude secondary diagnosis of D57)	J13 J14 J15.3 J15.4 J15.7 J15.9 J16.8 J18.1 J18.8
Tuberculosis	A15 A16 A19
Other vaccine-preventable diseases (in any diagnosis field)	A35 A36 A37 A80 B05 B06 B16.1 B16.9 B18.0 B18.1<br>B26 G00.0 M01.4

^Based on Bardsley et al. (2013)

#Based on the first diagnosis field in HES data unless otherwise specified.

Supplementary Table 2: Read codes used to identify SMI diagnostic categories

Category	Read codes used to identify diagnostic category
Schizophrenia and other psychoses	E100.00 E100.11 E100000 E100100 E100200 E100300 E100400 E100500 E100z00 E101.00 E101000 E101400 E101500 E101z00 E102.00 E102000 E102100 E102500 E102z00 E103.00 E103000 E103200 E103300 E103400 E103500 E103z00 E104.00 E105.00 E105000 E105200 E105500 E105z00 E106.00 E107.00 E107.11 E107000 E107100 E107200 E107300 E107400 E107500 E107z00 E10y.00 E10y.11 E10y000 E10y100 E10yz00 E10z.00 E120.00 E121.00 E122.00 E123.00 E123.11 E12y.00 E12y000 E12yz00 E12z.00 E13..00 E13..11 E131.00 E132.00 E133.00 E133.11 E134.00 E13y.00 E13y100 E13yz00 E13z.00 E13z.11 E1z..00 E212200 Eu20.00 Eu20000 Eu20011 Eu20100 Eu20111 Eu20200 Eu20211 Eu20212 Eu20213 Eu20214 Eu20300 Eu20311 Eu20400 Eu20500 Eu20511 Eu20600 Eu20y00 Eu20y12 Eu20y13 Eu20z00 Eu21.00 Eu21.11 Eu21.12 Eu21.13 Eu21.14 Eu21.15 Eu21.16 Eu21.17 Eu21.18 Eu22.00 Eu22000 Eu22011 Eu22012 Eu22013 Eu22014 Eu22015 Eu22100 Eu22111 Eu22200 Eu22300 Eu22y00 Eu22y11 Eu22y12 Eu22y13 Eu22z00 Eu23.00 Eu23000 Eu23011 Eu23012 Eu23100 Eu23112 Eu23200 Eu23211 Eu23212 Eu23214 Eu23300 Eu23312 Eu23y00 Eu23z00 Eu23z11 Eu23z12 Eu24.00 Eu24.12 Eu24.13 Eu25.00 Eu25000 Eu25011 Eu25012 Eu25100 Eu25111 Eu25112 Eu25200 Eu25212 Eu25y00 Eu25z00 Eu25z11 Eu26.00 Eu2y.00 Eu2y.11 Eu2z.00 Eu2z.11 Eu44.14

Bipolar disorder and affective psychoses	E11..00 E11..12 E110.00 E110.11 E110000 E110100 E110200 E110300 E110400 E110600 E110z00 E111.00 E111000 E111100 E111200 E111300 E111400 E111500 E111600 E111z00 E112400 E113400 E114.00 E114.11 E114000 E114100 E114200 E114300 E114400 E114500 E114600 E114z00 E115.00 E115.11 E115000 E115100 E115200 E115300 E115400 E115500 E115600 E115z00 E116.00 E116000 E116100 E116200 E116300 E116400 E116500 E116600 E116z00 E117.00 E117000 E117100 E117200 E117300 E117400 E117500 E117600 E117z00 E11y.00 E11y000 E11y100 E11y300 E11yz00 E11z.00 E11z000 E11zz00 E130.00 E130.11 E13y000 Eu30.00 Eu30.11 Eu30000 Eu30100 Eu30200 Eu30211 Eu30212 Eu30y00 Eu30z00 Eu30z11 Eu31.00 Eu31.11 Eu31.12 Eu31.13 Eu31000 Eu31100 Eu31200 Eu31300 Eu31400 Eu31500 Eu31600 Eu31700 Eu31800 Eu31900 Eu31911 Eu31y00 Eu31y11 Eu31y12 Eu31z00 Eu32300 Eu32311 Eu32312 Eu32313 Eu32314 Eu32800 Eu33213 Eu33300 Eu33311 Eu33312 Eu33313 Eu33314 Eu33315 Eu33316 Eu3z.11

Rates of care indicators over the study period

Supplementary Figure 1 shows the rate of care indicators recorded by GP practices per patient-year for each financial year in our sample. This rate is the number of times an indicator is recorded in that year across all patients, divided by the number of patient-years of observation in that financial year (which takes into account partial years for patients who were not observed for the full financial year). For example, in 2007/08 the rate of annual reviews (composite measure) recorded was 1.06 per patient-year.

The CP indicator shows a decline over the study period until 2012/13, when there was an increase in the threshold of achievement required to attain the maximum payment. The original AR indicator shows a marked drop-off after it ceased to be incentivized in 2010/11, while the aggregate indicator composed of the 'health risk' checks shows less marked change but a small increase after the specific components began to be incentivized in 2011/12. The composite of these two AR indicators shows little change in response to the QOF rule changes.

Supplementary Figure 1: Rate of care indicators per patient-year

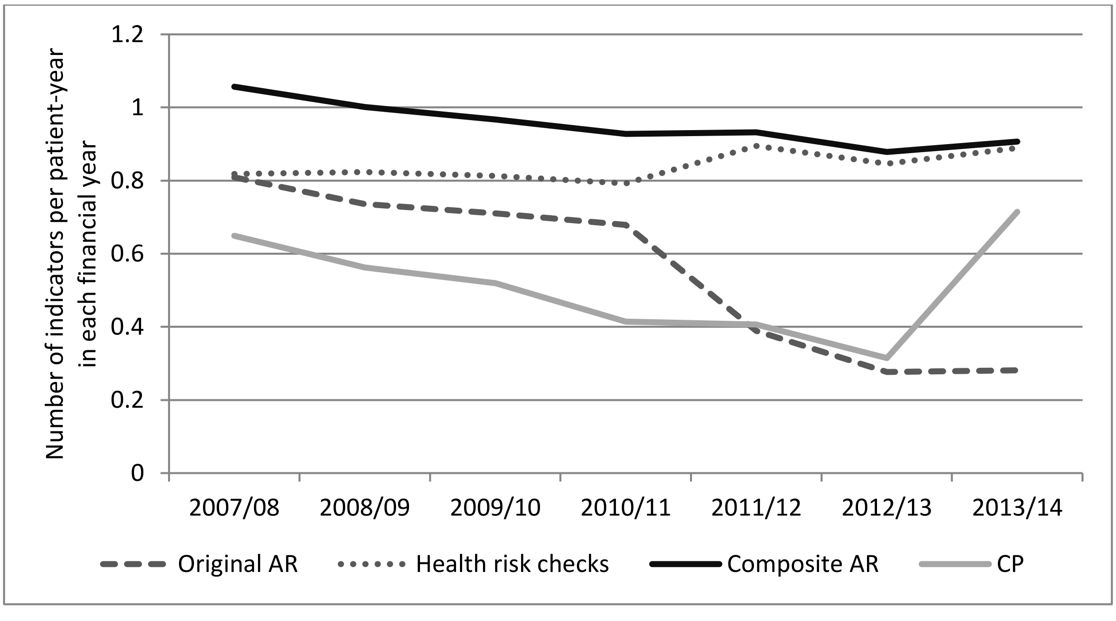

Survival analysis

We estimate the following hazard function for each outcome, which is the product of the baseline hazard, $\lambda_0(t)$, and an exponentiated linear function of patients' characteristics fixed at baseline (X_i) and care quality indicators for patient *i* at practice g at time *t*:

$$h_{ig}(t) = \lambda_{0_g}(t)\exp\{\beta_1 X_{ig1} + \cdots + \beta_k X_{igk} + \alpha_1 CP_{ig}^{cur.}(t) + \alpha_2 CP_{ig}^{exp.}(t) + \alpha_3 AR_{ig}(t)\} \quad (1)$$

The care quality indicators are represented by three time-varying variables:

$$CP_i^{cur.}(t) = \begin{cases} = 1 \text{ if a care plan has been documented in the last 12 months} \\ = 0 \text{ if a care plan has not been documented in the last 12 months} \end{cases}$$

$$CP_i^{exp.}(t) = \begin{cases} = 1 \text{ if a care plan has been documented before the last 12 months} \\ = 0 \text{ if a care plan has never been documented} \end{cases}$$

$$AR_i(t) = \begin{cases} = 1 \text{ if an annual review has been documented in the last 12 months} \\ = 0 \text{ if an annual review has not been documented in the last 12 months} \end{cases}$$

λ_{0_g}: the baseline hazard is allowed to vary by practice

Supplementary Table 3: Distribution of person-years observed across patient characteristics in sample for analysis of A&E presentations

Sample for A&E presentations	Person-years overall	Care plans: % of row person-years with current CP	Care plans: % of row person-years with expired CP	Care plans: % of row person-years with no CP	Annual reviews: % of row person-years with current AR	Annual reviews: % of row person-years with no current AR
Sample overall	6,654	40%	20%	40%	56%	44%
Age at diagnosis						
18-35	1,901	41%	19%	40%	49%	51%
36-45	1,496	41%	20%	39%	53%	47%
46-55	1.233	41%	23%	36%	62%	38%
56-65	842	39%	20%	41%	62%	38%
>65	1,181	37%	16%	47%	58%	42%
Index of multiple deprivation						
Quintile 1 (least deprived)	1,246	43%	21%	36%	59%	41%
Quintile 2	1,326	41%	185	41%	58%	42%
Quintile 3	1,188	37%	20%	43%	52%	48%
Quintile 4	1,464	40%	20%	40%	55%	45%
Quintile 5 (most deprived)	1,430	39%	19%	42%	54%	46%
Gender						
Male	3,131	41%	20%	39%	54%	46%
Female	3,522	39%	20%	41%	57%	43%
Ethnicity						
White	4,620	40%	19%	41%	56%	44%
Black & minority ethnicities	2,034	40%	21%	39%	54%	46%
Number of primary care contacts in year preceding diagnosis						
0-4	1,726	38%	22%	40%	49%	51%
5-9	1,894	42%	19%	39%	56%	44%
10-14	1,255	41%	18%	41%	58%	42%

Sample for A&E presentations	Person-years overall	Care plans			Annual reviews	
		% of row person-years with current CP	% of row person-years with expired CP	% of row person-years with no CP	% of row person-years with current AR	% of row person-years with no current AR
15-19	694	40%	18%	42%	59%	41%
>=20	1,085	40%	18%	42%	61%	39%
Number of hospital admissions in year preceding diagnosis						
0	4,119	38%	20%	42%	55%	45%
1	1,510	43%	21%	36%	56%	44%
2	611	46%	18%	36%	62%	38%
3	414	40%	19%	41%	57%	43%
Number of Charlson Index comorbidities at time of diagnosis						
0	4,346	40%	20%	40%	54%	46%
1	1,702	41%	20%	39%	58%	42%
2	405	34%	15%	51%	61%	39%
3 or more	201	31%	16%	52%	64%	36%
Comorbid depression at time of diagnosis						
History of depression	4,409	41%	19%	40%	56%	44%
No recorded history of depression	2,245	39%	21%	40%	54%	46%
Smoking status						
Current or ex-smoker	4,976	39%	19%	42%	56%	44%
No recorded history of smoking	1,678	42%	21%	37%	56%	44%
SMI diagnostic group						
Schizophrenia & other psychoses	3,427	40%	20%	40%	54%	46%
Bipolar disorder & affective psychoses	2,887	40%	19%	41%	57%	43%
Both	341	47%	22%	31%	61%	39%
Financial year of diagnosis						

Sample for A&E presentations	Person-years overall	Care plans			Annual reviews	
		% of row person-years with current CP	% of row person-years with expired CP	% of row person-years with no CP	% of row person-years with current AR	% of row person-years with no current AR
2007/08	1,407	43%	30%	27%	65%	35%
2008/09	1,265	40%	28%	32%	61%	39%
2009/10	1,159	38%	23%	39%	59%	41%
2010/11	931	41%	18%	41%	57%	43%
2011/12	937	38%	9%	53%	49%	51%
2012/13	684	39%	2%	59%	40%	60%
2013/14	272	39%	0	61%	29%	71%

Supplementary Table 4: Distribution of person-years observed across patient characteristics in sample for analysis of SMI admissions

Sample for SMI admissions	Person-years overall	Care plans			Annual reviews	
		% of row person-years with current CP	% of row person-years with expired CP	% of row person-years with no CP	% of row person-years with current AR	% of row person-years with no current AR
Sample overall	12,724	39%	27%	34%	59%	41%
Age at diagnosis						
18-35	3,825	40%	26%	34%	51%	29%
36-45	2,886	39%	29%	32%	57%	43%
46-55	2,080	40%	27%	33%	65%	35%
56-65	1,570	38%	29%	33%	68%	32%
>65	2,362	35%	24%	41%	63%	37%
Index of multiple deprivation						
Quintile 1 (least deprived)	2,255	39%	27%	34%	60%	40%
Quintile 2	2,394	40%	26%	34%	60%	40%
Quintile 3	2,327	35%	29%	36%	56%	44%
Quintile 4	2,874	40%	27%	33%	60%	40%
Quintile 5 (most deprived)	2,875	39%	26%	35%	58%	42%
Gender						
Male	5,976	38%	28%	34%	55%	45%
Female	6,748	39%	27%	34%	62%	38%
Ethnicity						
White	9,154	38%	27%	35%	59%	41%
Black & minority ethnicities	3,570	38%	28%	34%	58%	42%
Number of primary care contacts in year preceding diagnosis						
0-4	2,913	37%	29%	34%	50%	50%
5-9	3,587	39%	27%	34%	57%	43%
10-14	2,373	40%	27%	33%	60%	40%

Sample for SMI admissions	Person-years overall	Care plans			Annual reviews	
		% of row person-years with current CP	% of row person-years with expired CP	% of row person-years with no CP	% of row person-years with current AR	% of row person-years with no current AR
15-19	1,422	39%	25%	36%	63%	37%
>=20	2,429	38%	26%	36%	68%	32%
Number of hospital admissions in year preceding diagnosis						
0	7,723	37%	28%	35%	57%	43%
1	2,899	41%	27%	32%	60%	40%
2	1,192	42%	25%	33%	64%	36%
3	910	38%	26%	36%	62%	38%
Number of Charlson Index comorbidities at time of diagnosis						
0	7,970	39%	28%	33%	56%	44%
1	3,472	40%	27%	33%	62%	38%
2	849	33%	22%	45%	66%	34%
3 or more	432	31%	23%	46%	68%	32%
Comorbid depression at time of diagnosis						
History of depression	8,641	40%	27%	33%	60%	40%
No recorded history of depression	4,083	37%	27%	36%	56%	44%
Smoking status						
Current or ex-smoker	9,823	39%	27%	34%	59%	41%
No recorded history of smoking	2,901	39%	27%	34%	58%	42%
SMI diagnostic group						
Schizophrenia & other psychoses	6,394	39%	28%	33%	61%	39%
Bipolar disorder & affective psychoses	5,811	39%	28%	33%	61%	39%
Both	582	44%	30%	26%	63%	37%
Financial year of diagnosis						

Sample for SMI admissions	Person-years overall	Care plans			Annual reviews	
		% of row person-years with current CP	% of row person-years with expired CP	% of row person-years with no CP	% of row person-years with current AR	% of row person-years with no current AR
2006/07	2,752	38%	39%	23%	65%	35%
2007/08	2,077	40%	34%	26%	64%	36%
2008/09	2,006	38%	32%	30%	62%	38%
2009/10	1,818	36%	29%	35%	59%	41%
2010/11	1,454	42%	21%	37%	60%	40%
2011/12	1,402	37%	12%	51%	50%	50%
2012/13	901	40%	3%	57%	42%	58%
2013/14	314	40%	0	60%	28%	72%

Supplementary Table 5: Distribution of person-years observed across patient characteristics in sample for analysis of ACSC admissions

Sample for ACSC admissions	Person-years overall	Care plans			Annual reviews	
		% of row person-years with current CP	% of row person-years with expired CP	% of row person-years with no CP	% of row person-years with current AR	% of row person-years with no current AR
Sample overall	13,203	40%	27%	33%	59%	41%
Age at diagnosis						
18-35	4,111	41%	27%	32%	52%	48%
36-45	2,990	40%	29%	31%	57%	43%
46-55	2,215	41%	29%	31%	65%	35%
56-65	1,618	41%	28%	31%	68%	32%
>65	2,269	37%	23%	40%	63%	37%
Index of multiple deprivation						
Quintile 1 (least deprived)	2,364	41%	27%	32%	61%	39%
Quintile 2	2,500	41%	27%	32%	61%	39%
Quintile 3	2,408	37%	29%	34%	56%	44%
Quintile 4	2,979	42%	26%	32%	60%	40%
Quintile 5 (most deprived)	2,952	40%	26%	34%	58%	42%
Gender						
Male	6,305	40%	27%	33%	56%	44%
Female	6,898	40%	27%	33%	62%	38%
Ethnicity						
White	9,527	40%	27%	33%	60%	40%
Black & minority ethnicities	3,676	39%	27%	34%	57%	43%
Number of primary care contacts in year preceding diagnosis						
0-4	3,209	38%	29%	33%	51%	49%
5-9	3,746	41%	27%	32%	58%	42%
10-14	2,415	41%	27%	32%	61%	39%

15-19	1,447	40%	25%	35%	63%	27%
>=20	2,386	39%	27%	34%	67%	33%
Number of hospital admissions in year preceding diagnosis						
0	8,010	38%	28%	34%	58%	42%
1	3,071	43%	27%	30%	60%	40%
2	1,221	44%	25%	31%	63%	37%
3	901	41%	26%	33%	63%	37%
Number of Charlson Index comorbidities at time of diagnosis						
0	8,502	40%	28%	32%	57%	43%
1	3,534	41%	27%	32%	62%	38%
2	778	35%	22%	43%	66%	34%
3 or more	389	30%	23%	47%	68%	32%
Comorbid depression at time of diagnosis						
History of depression	8,860	41%	27%	32%	60%	40%
No recorded history of depression	4,343	38%	28%	34%	57%	43%
Smoking status						
Current or ex-smoker	10,118	40%	27%	33%	60%	40%
No recorded history of smoking	3,085	40%	28%	32%	58%	42%
SMI diagnostic group						
Schizophrenia & other psychoses	6,687	40%	26%	34%	57%	43%
Bipolar disorder & affective psychoses	5,820	40%	28%	32%	61%	39%
Both	696	44%	32%	24%	66%	34%
Financial year of diagnosis						
2006/07	2,884	39%	39%	21%	65%	35%
2007/08	2,151	42%	34%	24%	65%	35%
2008/09	2,033	40%	31%	29%	63%	37%
2009/10	1,936	38%	30%	32%	60%	40%
2010/11	1,511	42%	22%	36%	60%	40%

2011/12	1,438	38%	12%	50%	50%	50%
2012/13	932	41%	2%	56%	43%	57%
2013/14	319	40%	0	60%	29%	71%

Supplementary Table 6: Prevalence of Charlson Index comorbidities at time of SMI diagnosis

Comorbidities at time of SMI diagnosis	Proportion of sample (%)
Cancer	7.31
Cerebrovascular disease	5.08
COPD/ Asthma	19.72
CHD	2.12
Dementia	3.06
Diabetes	7.37
Hemiplegia	0.60
Liver disease	0.78
Myocardial infarction	2.68
Peptic ulcer	2.54
Peripheral vascular disease	1.96
Renal disease	7.60
Rheumatological disease	2.56

Supplementary Table 7: Sensitivity tests with A&E presentation as the outcome

Model specifications	Current AR		Current CP		Expired CP	
	HR	(95% CI)	HR	(95% CI)	HR	(95% CI)
Main analysis	0.96	(0.86-1.08)	0.87*	(0.77-0.98)	0.81*	(0.67-0.97)
AR using QOF codes only (limited to 2010/11)	0.92	(0.74-1.15)	0.88	(0.68-1.15)	0.64*	(0.42-0.99)
CP and AR current for 15 months	0.93	(0.83-1.05)	0.87*	(0.77-0.98)	0.85	(0.69-1.04)
Number of Charlson index comorbidities varies over time	0.96	(0.85-1.07)	0.87*	(0.77-0.98)	0.81*	(0.67-0.98)
Observed practice characteristics and cluster-adjusted SEs	0.95	(0.86-1.05)	0.89*	(0.80-0.99)	0.82*	(0.69-0.96)
Fixed practice effects and cluster-adjusted SEs	0.96	(0.86-1.06)	0.87*	(0.77-0.97)	0.83*	(0.70-0.98)

*p<0.05, **p<0.01, ***p<0.001

CP: Care plan (base: never had a care plan)

AR: Annual review (base: no annual review in last 12 months)

Hazard ratios represent the relative hazard of a patient with a care indicator compared with an otherwise identical patient with the base level care indicator.

Supplementary Table 8: Sensitivity tests with SMI admission as the outcome

Model specifications	Current AR		Current CP		Expired CP	
	HR	(95% CI)	HR	(95% CI)	HR	(95% CI)
Main analysis	1.06	(0.84-1.35)	1.02	(0.81-1.28)	0.90	(0.63-1.29)
AR using original codes only (limited to 2010/11)	1.03	(0.72-1.47)	1.01	(0.69-1.47)	0.84	(0.46-1.53)
CP and AR current for 15 months	1.06	(0.82-1.36)	1.00	(0.79-1.27)	1.00	(0.68-1.48)
Number of Charlson index comorbidities varies over time	1.08	(0.86-1.37)	1.00	(0.80-1.26)	0.89	(0.62-1.27)
Observed practice characteristics and cluster-adjusted SEs	1.08	(0.87-1.34)	1.05	(0.84-1.31)	0.98	(0.72-1.32)
Fixed practice effects and cluster-adjusted SEs	1.11	(0.88-1.40)	1.06	(0.85-1.33)	1.04	(0.75-1.43)

*p<0.05, **p<0.01, ***p<0.001

CP: Care plan (base: never had a care plan)

AR: Annual review (base: no annual review in last 12 months)

Hazard ratios represent the relative hazard of a patient with a care indicator compared with an otherwise identical patient with the base level care indicator.

Supplementary Table 9: Sensitivity tests with ACSC admission as the outcome

Model specifications	Current AR		Current CP		Expired CP	
	HR	(95% CI)	HR	(95% CI)	HR	(95% CI)
Main analysis	1.13	(0.87-1.47)	0.77*	(0.60-0.99)	0.88	(0.60-1.28)
AR using original codes only (limited to 2010/11)	0.85	(0.57-1.28)	1.07	(0.67-1.70)	0.84	(0.44-1.58)
CP and AR current for 15 months	1.08	(0.83-1.41)	0.79	(0.61-1.02)	0.90	(0.62-1.30)
Number of Charlson index comorbidities varies over time	1.08	(0.83-1.42)	0.79	(0.61-1.02)	0.92	(0.62-1.36)
Observed practice characteristics and cluster-adjusted SEs	1.05	(0.84-1.32)	0.79*	(0.63-0.99)	0.88	(0.63-1.22)
Fixed practice effects and cluster-adjusted SEs	1.03	(0.81-1.30)	0.82	(0.64-1.05)	0.95	(0.66-1.35)

*p<0.05, **p<0.01, ***p<0.001

CP: Care plan (base: never had a care plan)

AR: Annual review (base: no annual review in last 12 months)

Hazard ratios represent the relative hazard of a patient with a care indicator compared with an otherwise identical patient with the base level care indicator.
