# Supplementary material for: Do care plans and annual reviews of physical health influence unplanned hospital utilisation for people with serious mental illness? Analysis of linked longitudinal primary and secondary healthcare records in England
Source: BMJ Open. 2018 Nov 28;8(11):e023135. doi: 10.1136/bmjopen-2018-023135 (PMC6278786; doi:10.1136/bmjopen-2018-023135)
Supplement: Author's manuscript [file bmjopen-2018-023135.draft_revisions.pdf]

BMJ Open is committed to open peer review. As part of this commitment we make the peer review history of every article we publish publicly available.

When an article is published we post the peer reviewers' comments and the authors' responses online. We also post the versions of the paper that were used during peer review. These are the versions that the peer review comments apply to.

The versions of the paper that follow are the versions that were submitted during the peer review process. They are not the versions of record or the final published versions. They should not be cited or distributed as the published version of this manuscript.

BMJ Open is an open access journal and the full, final, typeset and author-corrected version of record of the manuscript is available on our site with no access controls, subscription charges or pay-per-view fees (http://bmjopen.bmj.com).

If you have any questions on BMJ Open's open peer review process please email info.bmjopen@bmj.com

BMJ Open

Do care plans and annual reviews of physical health influence unplanned hospital utilisation for people with serious mental illness? Analysis of linked longitudinal primary and secondary healthcare records

Journal:	*BMJ Open*
Manuscript ID	bmjopen-2018-023135
Article Type:	Research
Date Submitted by the Author:	23-Mar-2018
Complete List of Authors:	Ride, Jemimah; University of York Centre for Health Economics,<br>Kasteridis, Panagiotis; University of York Centre for Health Economics<br>Gutacker, Nils; University of York, Centre for Health Economics<br>Kronenberg, Christoph; University Duisburg-Essen, CINCH ; Leibniz Science Campus Ruhr<br>Doran, Tim; University of York, Health Sciences<br>Mason, Anne; University of York, Centre for Health Economics<br>Rice, Nigel; University of York Centre for Health Economics<br>Gravelle, Hugh; University of York Centre for Health Economics<br>Goddard, Maria; University of York, Centre for Health Economics<br>Kendrick, Tony; University of Southampton, Primary Care and Population Sciences<br>Siddiqi, Najma; University of York, Psychiatry, Hull York Medical School, York and Bradford District Care NHS Foundation Trust, Bradford, UK<br>Gilbody, Simon; The University of York, Department of Health Sciences<br>Dare, Ceri; McPin Foundation,<br>Aylott, Lauren<br>Williams, Rachael; Clinical Practice Research Datalink,<br>Jacobs, Rowena; University of York, Centre for Health Economics
Keywords:	Schizophrenia & psychotic disorders < PSYCHIATRY, PRIMARY CARE, Quality in health care < HEALTH SERVICES ADMINISTRATION & MANAGEMENT, Data linkage, Hospital utilisation

SCHOLARONE™
Manuscripts

Do care plans and annual reviews of physical health influence unplanned hospital utilisation for people with serious mental illness? Analysis of linked longitudinal primary and secondary healthcare records

Authors:

Jemimah Ride (corresponding author): Centre for Health Economics, Alcuin 'A' Block, University of York, Heslington, YO10 5DD, United Kingdom. jemimah.ride@york.ac.uk (+44) 01904 321 459

Panos Kasteridis[a] (panos.kasteridis@york.ac.uk); Nils Gutacker[a] (nils.gutacker@york.ac.uk); Christoph Kronenberg[b] (christoph.kronenberg@uni-due.de); Tim Doran[c] (tim.doran@york.ac.uk); Anne Mason[a] (anne.mason@york.ac.uk); Nigel Rice[a] (nigel.rice@york.ac.uk); Hugh Gravelle[a] (hugh.gravelle@york.ac.uk); Maria Goddard[a] (maria.goddard@york.ac.uk); Tony Kendrick[d] (A.R.Kendrick@soton.ac.uk); Najma Siddiqi[c] (najma.siddiqi@york.ac.uk); Simon Gilbody[c] (simon.gilbody@york.ac.uk); Ceri R Dare[e] (ceri.owen@gmail.com); Lauren Aylott[e] (lmeaylott@gmail.com); Rachael Williams[f] (Rachael.Williams@mhra.gov.uk); Rowena Jacobs[a] (rowena.jacobs@york.ac.uk)

a: Centre for Health Economics, University of York

b: CINCH, University Duisburg-Essen, Essen, Germany; Leibniz Science Campus Ruhr, Essen, Germany; RWI – Leibniz-Institute for Economic Research, Essen, Germany

c: Department of Health Sciences, University of York

d: Primary Care and Population Sciences, University of Southampton

e: Service user

f: Clinical Practice Research Datalink

Word count: 3,689

For peer review only - http://bmjopen.bmj.com/site/about/guidelines.xhtml

ABSTRACT

Objective: To investigate whether two primary care activities that are framed as indicators of primary care quality (comprehensive care plans and annual reviews of physical health) influence unplanned utilisation of hospital services for people with serious mental illness (SMI).

Design & setting: Retrospective observational cohort study using linked primary care and hospital records (Hospital Episode Statistics) for 5,158 patients diagnosed with SMI between April 2006 and March 2014, who attended 213 primary care practices in England that contribute to the Clinical Practice Research Datalink GOLD database. Cox duration models were used to estimate the associations between two primary care quality indicators (care plans and annual reviews of physical health) and the hazards of three types of unplanned hospital utilisation: presentation to Accident & Emergency departments (A&E), admission for SMI, and admission for ambulatory care sensitive conditions.

Results: Risk of A&E presentation was 14% lower (hazard ratio (HR) 0.86, 95% CI 0.76-0.98) for patients with a care plan documented in the previous year compared to those without a care plan, and 19% lower for those who had a care plan documented earlier but not updated in the previous year (HR: 0.81, 95% CI 0.67-0.97). There was no association between risk of A&E presentation and a review of physical health in the previous year. Risks of hospital admission for SMI or for ambulatory care sensitive conditions were not associated with care plans or annual reviews.

Conclusions*:* Care plans for people with SMI are associated with reduced risk of A&E attendance, but not with risk of SMI or ambulatory care sensitive hospital admissions.

For peer review only - http://bmjopen.bmj.com/site/about/guidelines.xhtml

Strengths and limitations of this study

- Innovative use of linked individual-level patient data from primary care, inpatient admissions and A&E, allowing the sequence of care indicators and unplanned hospital utilisation to be identified.
- Contributes to a limited evidence base on the association of patient outcomes with the types of care incentivised under a national primary care physician incentive scheme.
- Due to the observational nature of the data, no information was available on the circumstances leading to documentation of care quality indicators, or their quality or content.
- Partial capacity to account for patient health status and factors driving utilisation of primary and secondary care.

Keywords

Schizophrenia & psychotic disorders; primary care; quality in health care; data linkage; hospital utilisation.

Funding statement

This project was funded by the National Institute for Health Research HS&DR programme (project number 13/54/40). The views expressed are those of the author(s) and not necessarily those of the NHS, the NIHR or the Department of Health.

Competing interests

No competing interests.

For peer review only - http://bmjopen.bmj.com/site/about/guidelines.xhtml

INTRODUCTION

Serious mental illness (SMI) comprises a set of conditions including schizophrenia and bipolar disorder with profound impacts on the wellbeing of patients and high costs to society. It is linked with a high disease burden [1 2], poor health outcomes, high treatment costs, and lower life expectancy, primarily attributed to preventable physical causes [3-6]. Patients with SMI have high rates of A&E attendance and hospital admission, for both physical and mental health problems[7-10].

In the UK, while specialist mental health services are important to many patients' care, general practice provides the majority of care for patients with SMI [11]. High quality primary care therefore has the potential to improve the management, health and wellbeing of patients with SMI, and can help meet the aims of policymakers seeking to improve population wellbeing and contain costs [12 13].

Several countries have adopted incentive programs to improve the quality and value of healthcare [14], despite mixed evidence of their effectiveness [15-17]. England introduced a voluntary scheme for primary care in 2004, the Quality and Outcomes Framework (QOF), offering GPs incentives to meet quality targets for patients with several chronic conditions, including SMI [18]. Two key SMI indicators in the QOF, which apply to all registered patients with SMI, promote proactive management of physical and mental health: the proportion of patients on the practice SMI register who had an in-date comprehensive care plan, and the proportion who had an annual physical health review.

For peer review only - http://bmjopen.bmj.com/site/about/guidelines.xhtml

For peer review only

There is little evidence on whether these incentivised activities improve patients' mental or physical health, modify patterns of healthcare utilisation, or reduce costs. They derive from NICE guideline recommendations for primary care management of SMI, which are based on consensus in the absence of robust evidence [19 20]. As the indicators are regularly reviewed and revised, new evidence on their potential impacts is useful for decision-makers. Our study contributes such evidence, examining whether care plans and annual reviews are associated with utilisation of unplanned secondary care for patients with SMI, by analysing linked primary care and hospital records.

Quality indicators

A good quality care plan documents the patient's current health and social care needs, and how these will be addressed, in agreement with patients and caregivers. It should specify arrangements with secondary care services (where applicable), and recommendations in case of relapse, including the patient's care preferences and goals [19 20]. The QOF incentivises GPs to document care plans annually, so we analyse 'current' care plans, those recorded in the last twelve months. However, we hypothesise that an older care plan may still influence patients' health service utilisation, and therefore also analyse 'expired' care plans, those recorded more than a year ago.

The annual review of physical health indicator changed over the study period. From 2006/7 to 2010/11 the annual review indicator entailed the patient being given appropriate health promotion and prevention advice. In 2011/12, this broad indicator was split into more specific indicators: a record of alcohol consumption, checks of

For peer review only - http://bmjopen.bmj.com/site/about/guidelines.xhtml

blood pressure, body mass index (BMI), blood glucose or glycated haemoglobin, and ratio of total to high-density lipoprotein cholesterol, and, where appropriate, cervical screening. Unlike care plans, only ‘current’ annual reviews (recorded within the last twelve months) are included in the analysis, since expired monitoring of physical health is unlikely to have ongoing benefits.

A previous study using data aggregated to practice level found that higher rates of SMI admission were associated with higher achievement on the annual physical review indicator, but not the care plan indicator [21]. However, that study could not ascertain which individuals within a practice received care or whether the care preceded or followed admissions. An innovation of the current study is that we observe the sequence of events for individual patients (dates of SMI diagnosis, care indicators, and hospital utilisation), which allows us to identify whether the care indicator precedes the outcome of interest.

We hypothesise that care plans and annual reviews may modify risk of three outcomes: presentations to Accident and Emergency departments (A&E), unplanned hospital admission for SMI, and unplanned admissions for ambulatory care sensitive conditions (ACSCs), which are potentially preventable by high-quality primary care [22]. Patients with SMI are less likely to report health problems than their peers due to self-neglect, impaired motivation, and social withdrawal [23 24]. Proactive care by GPs may help to identify and manage physical and mental health problems without the need for hospital care.

For peer review only - http://bmjopen.bmj.com/site/about/guidelines.xhtml

DATA

We analyse linked primary care, inpatient care, and A&E data for patients with a diagnosis of SMI. The main data source is the Clinical Practice Research Datalink (CPRD) GOLD, which holds linked individual-level anonymized primary care records from participating general practices in the UK. These data are representative of the English population with respect to age and gender, but not region. For example, the north-east of England is under-sampled relative to areas in the west and south [25].

The CPRD data service provided information on all patients eligible for linkage and registered with a participating practice in England, and with a diagnosis of SMI documented on or before 31 March 2014. Most of the primary care information is recorded using a hierarchical coding system known as 'Read codes' [26], which we use to identify records of care quality indicators, morbidity profiles, and smoking and alcohol consumption status.

Data linkages

The Hospital Episode Statistics (HES) comprise detailed records for all NHS patients admitted to hospital and presentations to A&E in England. CPRD provided deterministically linked admission and A&E data for all patients included in the study. Unplanned admissions were classified for SMI by primary diagnosis International Classification of Diseases (ICD-10) codes, categories F20-F31 [27], and for chronic ACSC as defined by Bardsley, et al. [28]. We specified chronic ACSCs since these would be most amenable to the types of GP care we investigate and are a proxy for avoidable admissions [29]. A&E presentations of any cause (mental or physical health) were included.

For peer review only - http://bmjopen.bmj.com/site/about/guidelines.xhtml

To preserve anonymity, all linkages were carried out under the CPRD routine linkage scheme. The study was approved by the Independent Scientific Advisory Committee (Protocol 14_168).

METHODS

Sample

Our sample covers eight financial years, from 1 April 2006 to 31 March 2014. The care plan indicator was introduced in 2006/07 and our last year of HES data is 2013/14. The sample comprises patients whose earliest recorded diagnosis of SMI in primary care was after 31 March 2006, and who were registered with the same practice for at least 365 days before that diagnosis. A&E data are only available from 2007/08, so the analysis of A&E presentations is limited to patients with a first diagnosis after 31 March 2007. The restriction to newly diagnosed patients excludes those whose unobserved past care or events could influence their subsequent care. We use data from the year prior to diagnosis to capture elements of medical history. Following clinical advice, we constructed three diagnostic categories: schizophrenia and other psychoses (group 1), bipolar disorder and affective psychoses (group 2) [1, 2], and those who had a diagnosis from each group applied at some point. (See Supplementary Table 1 for the list of codes used to identify each category.)

In order to explore annual reviews for the full period of our analysis, given the changes to the annual review incentives in 2011/12, we formulate an aggregate indicator. This signifies that the patient had at least three of four 'health risk' checks (blood pressure, BMI, cholesterol, and glucose) documented within a three month

For peer review only - http://bmjopen.bmj.com/site/about/guidelines.xhtml

period [30]. The date of the aggregate indicator is the date of the final check. In our analysis, patients are considered to have a 'current' annual review for twelve months after either an annual review (using the original Read coding), or from the date of this aggregate indicator.

Study design

We investigate the relationship between having a current or expired care plan or a current annual physical health review, and the occurrence of three separate outcomes: A&E presentations, SMI admissions, and ACSC admissions. We use duration analysis to examine how time to an outcome is affected by receipt of care plans and annual reviews. Our approach accommodates the time-varying nature of care plans and annual reviews, which do not necessarily occur at consistent intervals after diagnosis.

Patient involvement

Two co-authors on the multidisciplinary team responsible for this study had lived experience of SMI. They contributed to the design of the research questions and methodological approach, interpretation of the findings, and writing the paper.

Analytical approach

We estimate Cox proportional hazard models [31] with the same basic structure, examining duration from SMI diagnosis to each of the three outcomes: first A&E presentation, first SMI admission, and first ACSC admission. We follow each individual until the outcome of interest or until censoring. Censoring can occur

For peer review only - http://bmjopen.bmj.com/site/about/guidelines.xhtml

because i) a patient dies, ii) registration with the practice ends, or iii) the study period ends (i.e. the patient is still registered on 31 March 2014).

We control for demographic variables at baseline including gender, ethnicity, and age at first diagnosis (the start of the observation period). To account for patients' state of health at entry to the study, we include the number of Charlson comorbidities [32] listed in their primary care records, and whether a diagnosis of depression was recorded. Patients' history of alcohol consumption and smoking are classified as binary variables (ever/ never) according to whether a relevant diagnostic or management code was ever recorded in CPRD GOLD before the first SMI diagnosis. Historical healthcare utilisation is captured by the number of GP contacts (face-to-face visits and telephone calls) and the number of inpatient admissions (all-cause) in the year preceding diagnosis, and we control for the calendar year of first diagnosis.

Survival analysis

We estimate the following hazard function for each outcome, which is the product of the baseline hazard, $\lambda_0(t)$, and an exponentiated linear function of patients' characteristics and three care indicator variables for current and expired care plans and (current only) annual physical reviews:

$$h_{ig}(t) = \lambda_{0_g}(t)\exp\{\beta_1 X_{ig1} + \cdots + \beta_k X_{igk} + \alpha_1 CP_{ig}^{cur.}(t) + \alpha_2 CP_{ig}^{exp.}(t) + \alpha_3 AR_{ig}(t)\} \quad (1)$$

Patient demographic and medical characteristics, $X_{i1},\ldots X_{iK,}$ are assumed to be fixed over time, whereas the three care indicators may vary over time. We stratify on practice, g, so that the baseline hazard varies across practices, to allow for unobserved differences in patient populations and practice characteristics.

For peer review only - http://bmjopen.bmj.com/site/about/guidelines.xhtml

Our design deals with irregularity in the frequency and timing of care indicators through the three time-varying variables. The base case is a patient who has never received a care plan and had no annual review of physical health within the last twelve months. Two variables indicate whether a care plan or annual review has been recorded within the last twelve months (‘current care plan’ and ‘current annual review’). If a patient receives a further care plan or annual review within the twelve-month window, the period of the ‘current’ period is extended accordingly. The third variable indicates that the patient received a care plan more than twelve months ago (‘expired care plan’). The choice of the twelve month window to determine expiration status is based on the QOF guidance that a care plan or annual review should be reviewed annually [3].

Standard errors are clustered at practice level to account for within-practice correlation. Coefficient estimates are presented as hazard ratios (HRs) with associated 95% confidence intervals (CIs), where values greater than 1 indicate an increase in the hazard of the outcome associated with a unit change in the explanatory variable, and vice versa for a HR below 1. All analyses were performed in Stata 14 (StataCorp LP, College Station, TX, US).

Sensitivity analyses

We undertake five tests of the sensitivity of our findings to alternate specifications of the model. First, we test the robustness of the annual review variable, which in our main analysis is the composite indicator constructed from both the original annual review codes and the specific health checks that contribute to such reviews. We

For peer review only - http://bmjopen.bmj.com/site/about/guidelines.xhtml

explore the impact of restricting this to the original annual review codes, which necessitates limiting the observation period to 2006/07 to 2010/11 (or 2007/08 to 2010/11 for the A&E outcome). Second, we assume the care indicators expire after 15 months instead of 12 months. Third, instead of fixing the number of Charlson index comorbidities at baseline, we allow this to increase over the period of observation if patients are diagnosed with new comorbidities. We lastly explore the sensitivity of the results to two alternate model specifications of practice-level characteristics, instead of stratifying on practice. One assumes no practice-level differences in baseline hazard, but includes practice characteristics (rurality and distance from closest acute hospital and inpatient psychiatric unit) as covariates. The second includes practice fixed effects as explanatory variables. Both models adjust the standard errors for clustering at the practice level as before.

DESCRIPTIVE STATISTICS

The sample consists of 5,158 newly diagnosed patients with SMI from 213 practices in England and covers the period 1 April 2006 to 31 March 2014 (for A&E analysis 4,446 patients from 213 practices from 1 April 2007 to 31 March 2014). Summary statistics for the care quality and outcome variables are presented in Table 1. Over the study period, 69% of patients received at least one care plan (average 1.2 annually in those who had at least one), while 72% received at least one annual review or equivalent composite health checks (average 2.1 annually). More detail on the annual rates of care plan and annual review indicators is available in supplementary material (Supplementary Figure 1).

For peer review only - http://bmjopen.bmj.com/site/about/guidelines.xhtml

Table 1. Main survival analysis: descriptive statistics

	Full sample	At least one CP^	At least one AR^
A&E analysis sample			
N	4446	2398	2567
N with A&E presentation	2213	1006	1879
Person-years#^	6660	5156	5608
Mean days till outcome	396	624	610
Events per person-year	0.33	0.20	0.34
SMI analysis sample			
N	5158	3332	3537
N with SMI admission	566	279	313
Person-years#^	12860	10525	11420
Mean days till outcome	448	755	730
Events per person-year	0.04	0.03	0.03
ACSC analysis sample			
N	5158	3515	3681
N with ACSC admission	180	102	131
Person-years#^	14067	11745	12589
Mean days till outcome	693	948	887
Events per person-year	0.01	0.01	0.01

^Observed before outcome event or censoring.
#Person-years is the sum of observation periods for the sample, taking into account partial years.
CP: Care plan. AR: Annual review of physical health.

During the period of observation, 49.8% had an A&E presentation, 11% had an SMI admission, 3.5% had an ACSC admission, and 10.5% died. Summary statistics for the explanatory variables for the full sample are presented in Table 2. The mean age at diagnosis in the sample was 50, and the most common physical comorbidities were respiratory disease, renal disease, cancer, and diabetes (further details available in Supplementary Table 2).

For peer review only - http://bmjopen.bmj.com/site/about/guidelines.xhtml

Table 2. Explanatory variables

	N (%)
Age at diagnosis	
18-35	1,540 (30)
36-45	1,008 (20)
46-55	736 (14)
56-65	561 (11)
>65	1,313 (25)
Index of multiple deprivation	
Quintile 1 (least deprived)	925 (18)
Quintile 2	980 (19)
Quintile 3	951 (18)
Quintile 4	1,150 (22)
Quintile 5 (most deprived)	1,155 (22)
Male	2,430 (47)
White	4,233 (82)
Number of primary care contacts in year preceding diagnosis	
0-4	1,143 (22)
5-9	1,341 (26)
10-14	946 (18)
15-19	618 (12)
>=20	1,110 (22)
Number of hospital admissions in year preceding diagnosis	
0	2,883 (56)
1	1,237 (24)
2	552 (11)
3	486 (9)
Number of Charlson Index comorbidities at time of diagnosis	
0	3,047 (59)
1	1,384 (27)
2	446 (9)
3 or more	281 (5)
History of depression at time of diagnosis	3,358 (65)
Current or ex-smoker	3,960 (77)
Current or ex-alcohol consumption	3,656 (71)
SMI diagnostic group	
Schizophrenia & other psychoses	2,884 (56)
Bipolar disorder & affective psychoses	2,078 (40)
Both	196 (4)
Total number of patients	5,158

For peer review only

For peer review only - http://bmjopen.bmj.com/site/about/guidelines.xhtml

RESULTS OF DURATION ANALYSIS

Results for each of the outcomes are presented in Figure 1. Since our research question addresses the relationship between care quality indicators and outcomes, we focus on the HRs for the three key explanatory variables: current annual review, current care plan and expired care plan. HRs for other explanatory variables are presented in Supplementary Table 3.

[Figure 1 about here]

The hazard of A&E presentation is 14% lower for patients who have a current care plan (HR 0.86, 95% CI 0.76-0.98) and 19% lower for those with an expired care plan (HR 0.81, 95% CI 0.67-0.97), but there is no statistically significant association with annual reviews of physical health (HR 0.96, 95% CI 0.86-1.08). Neither indicator shows a statistically significant association with the hazard of either first SMI admission or first ACSC admission after SMI diagnosis.

Table 3 shows the results of sensitivity testing. Under all assumptions tested, there was no association between annual reviews of physical health and A&E presentations, or between care plans and the two types of hospital admissions. The association between care plans and A&E presentations remained essentially unchanged with alternate practice effects specifications, and with the use of time-varying comorbidity instead of fixing this at baseline. In sensitivity tests that use the original annual review indicator instead of the composite indicator, the association between annual review and all three outcomes remained unchanged. However, the association between

For peer review only - http://bmjopen.bmj.com/site/about/guidelines.xhtml

current care plans and A&E attendance became non-significant as confidence intervals widened (this analysis was limited to 2007/08 – 2010/11 when the original indicator was in use). Assuming that the indicators were effective (current) for 15 months instead of 12 did not change the association between current care plans and hazard of A&E, but there was no longer an association with expired care plans (written more than 15 months ago rather 12 months).

For peer review only

For peer review only - http://bmjopen.bmj.com/site/about/guidelines.xhtml

Table 3. Sensitivity testing - hazard ratios for care indicator variables

Model specifications	Care indicator	A&E presentation HR	A&E presentation (95% CI)	SMI admission HR	SMI admission (95% CI)	ACSC admission HR	ACSC admission (95% CI)
Main analysis	Current AR	0.96	(0.86-1.08)	1.08	(0.85-1.36)	1.21	(0.78-1.87)
	Current CP	0.86*	(0.76-0.98)	1.01	(0.80-1.27)	0.89	(0.58-1.36)
	Expired CP	0.81*	(0.67-0.97)	0.90	(0.63-1.28)	0.91	(0.48-1.71)
AR using original codes only (limited to 2010/11)	Current AR	0.92	(0.74-1.15)	1.05	(0.74-1.51)	0.78	(0.38-1.57)
	Current CP	0.88	(0.68-1.15)	1.00	(0.69-1.46)	1.38	(0.62-3.08)
	Expired CP	0.64*	(0.42-0.99)	0.84	(0.46-1.53)	1.39	(0.44-4.38)
CP and AR current for 15 months	Current AR	0.94	(0.83-1.05)	1.07	(0.83-1.38)	1.06	(0.66-1.69)
	Current CP	0.87*	(0.77-0.98)	0.99	(0.79-1.26)	0.92	(0.60-1.42)
	Expired CP	0.85	(0.69-1.04)	1.00	(0.68-1.47)	0.94	(0.48-1.84)
Number of Charlson index comorbidities varies over time	Current AR	0.96	(0.85-1.07)	1.10	(0.87-1.39)	1.19	(0.76-1.85)
	Current CP	0.87*	(0.77-0.98)	1.00	(0.79-1.25)	0.90	(0.58-1.39)
	Expired CP	0.81*	(0.67-0.97)	0.89	(0.62-1.26)	0.99	(0.54-1.83)
Observed practice characteristics and cluster-adjusted SEs	Current AR	0.95	(0.76-1.05)	1.09	(0.88-1.35)	1.33	(0.91-1.94)
	Current CP	0.89*	(0.80-0.99)	1.05	(0.84-1.31)	0.83	(0.57-1.21)
	Expired CP	0.82*	(0.69-0.96)	0.97	(0.72-1.32)	0.95	(0.57-1.58)
Fixed practice effects and cluster-adjusted SEs	Current AR	0.96	(0.86-1.06)	1.11	(0.88-1.41)	1.29	(0.87-1.92)
	Current CP	0.87*	(0.77-0.97)	1.05	(0.84-1.32)	0.85	(0.57-1.29)
	Expired CP	0.83*	(0.70-0.98)	1.03	(0.75-1.41)	0.99	(0.57-1.74)

*p<0.05, **p<0.01, ***p<0.001 CP: Care plan. AR: Annual review
#Hazard ratios represent the relative hazard of a patient with a care indicator compared with an otherwise identical patient with no care indicator.

For peer review only

For peer review only - http://bmjopen.bmj.com/site/about/guidelines.xhtml

DISCUSSION

Principal findings

Our study examines the relationship between two primary activities framed as care quality indicators (care plans and annual physical health reviews) for patients with SMI, and three measures of secondary healthcare utilization: A&E presentations, SMI admissions, and ACSC admissions. Although care plans and annual physical health reviews were incentivised by the QOF we do not test the effect of incentives. Our research question is whether care plans or annual reviews are influence unplanned hospital utilisation. We find that care plans are associated with a lower hazard of A&E presentation, but not with risk of hospital admissions for SMI or ACSC, and we find no association between annual reviews and the hazard of any of the outcomes.

Strengths and weaknesses

Our study makes a number of important contributions to the evidence base, being (to our knowledge) the first to use linked patient-level data to investigate the relationship between incentivized primary care quality and hospital care for people with SMI in the English NHS. By linking datasets, we track individual patients across primary care and hospital settings over a number of years, and determine the sequence of GP care and subsequent hospital utilisation. Our duration analysis exploits this, providing more robust estimates of the association between care quality indicators and patient's healthcare utilisation than would be possible with aggregate data.

There are a number of limitations to our study. The measures of health status and healthcare utilisation prior to diagnosis of SMI may not fully depict the complexities

For peer review only - http://bmjopen.bmj.com/site/about/guidelines.xhtml

of health status, including severity of SMI, and this may confound the results. However, our findings were robust to whether the number of comorbidities was fixed at baseline or allowed to vary over the period of observation. The outcomes we analyse are measures of hospital utilisation, which may act as imperfect proxies for health outcomes. Our finding of a negative association between care plans and A&E presentations may reflect the presence of unobserved factors that could contribute to the observed association without implying causality. We lack information on the clinical circumstances leading to a care plan or annual review being recorded, and on its content, quality and appropriateness. In the period preceding an A&E attendance, care planning may be superseded by acute management of physical or mental health conditions. Patients who have less insight or lower levels of self-care may be less likely to seek regular primary care, and therefore less likely to have care plans documented, but may also be more likely to seek A&E care. Conversely, our findings could underestimate any real effect of care plans on A&E presentations if other unobserved factors contribute to a (non-causal) positive association, such as care plans being triggered by patients attending their GP for problems that will eventually need hospital care.

We restricted the sample to patients registered with the same practice for the year preceding diagnosis in order to analyse a group of patients at a similar clinical stage, and allow us to include measures of historical utilization and medical history. However, the restriction may have excluded individuals with more severe SMI if this led them to move. In addition, the sample may include some patients who were not newly diagnosed, if their diagnostic information had not been transferred to a new practice, or to a new electronic recording system.

For peer review only - http://bmjopen.bmj.com/site/about/guidelines.xhtml

Interpretation of results and implications for clinicians and policymakers

The association of care plans with reduced hazard of A&E presentations but not with that of hospital admissions might suggest that care plans help patients avoid A&E for conditions that do not require hospital care. The documentation of patients' current health status, early warning signs, triggers, social support needs, co-ordination arrangements with secondary care, and preferred course of action in the event of a clinical relapse could improve the management of the patient's health overall and prevent deterioration, reducing the need for urgent care. It may also direct patients into appropriate services during periods of deterioration, and thereby avert A&E presentations.

Increasing demand for A&E care, especially for problems that could be managed elsewhere, is a policy focus in the UK NHS and in other health care systems [33]. Patients with SMI, in particular, can require high resource input when attending A&E [34 35]. The potential for care planning by GPs to reduce A&E attendances in this patient population could therefore be an important finding for policymakers, suggesting that resources deployed in primary care may reduce demand on secondary care services.

The associations of both 'current' and 'expired' care plans with lower hazard of A&E attendance suggest that an older care plan can still be beneficial. However, the finding that when care plans are considered effective for 15 months rather than 12 months, only 'current' care plans are associated with reduced A&E attendance suggests that there are limits to the duration of impact. The lack of association between annual reviews of physical health and unplanned hospital care may suggest that effectiveness

For peer review only - http://bmjopen.bmj.com/site/about/guidelines.xhtml

of a GP in managing a patient's health, and preventing the need for A&E or hospital care, may be unaffected by whether or not annual reviews or health checks are recorded.

Unanswered questions and future research

This study did not account for care provided by patients' key workers or a crisis resolution and home treatment team, which may provide an alternative to both hospital admission and primary care. The modelling approach does adjust for differences across practices, which may help control for some of these local effects. To address this more adequately, the Mental Health Services Dataset, which can now be linked to CPRD primary care data, could be used to cover mental health care delivered in the community.

Conclusion

Provider incentive schemes are increasingly popular levers for improving value and quality in healthcare worldwide, but evidence is needed on the effectiveness of incentivised activities. Despite the vulnerability of people with SMI to poor health outcomes, relatively few studies have examined the impact of care quality indicators in this population. This study addresses that evidence gap, and advances our understanding of how primary care can influence utilisation of hospital care in this patient population. We find that care plans are associated with reduced risk of A&E presentation, but not of hospital admissions, supporting the hypothesis that this type of care incentivized under the QOF is achieving at least some of its policy objectives.

For peer review only

For peer review only - http://bmjopen.bmj.com/site/about/guidelines.xhtml

For peer review only

For peer review only - http://bmjopen.bmj.com/site/about/guidelines.xhtml

Author contributions

JR, PK, NG, CK, TD, AM, NR, HG, MG, TK, NS, SG, CD, LA, RW and RJ contributed to the design of the research questions, analytical approach, and interpretation of the findings. JR produced a first draft of the paper and contributed to and PK, NG, CK, TD, AM, NR, HG, MG, TK, NS, SG, CD, LA, RW and RJ approved the final manuscript.

Data sharing

Due to the sensitive and confidential nature of the data used for this analysis, and the permissions required to access it, the dataset is not publicly available.

Endnotes

1. We use a twelve-month window rather than the calendar or financial year.
2. We consider any patients with unrelated hospitalisation (e.g. a planned admission for surgery) as having zero hazard of A&E presentation and SMI or ACSC admission during the time they are hospitalised.

Figure 1 legend

Figure 1. Association of care quality indicators with each outcome.

CP: Care plan

AR: Annual review of physical health

For peer review only

For peer review only - http://bmjopen.bmj.com/site/about/guidelines.xhtml

REFERENCES

1. Murray CJ, Richards MA, Newton JN, et al. UK health performance: findings of the Global Burden of Disease Study 2010. The Lancet 2013;**381**(9871):997-1020.
2. Vigo D, Thornicroft G, Atun R. Estimating the true global burden of mental illness. The Lancet Psychiatry 2016;**3**(2):171-78.
3. Osborn DP, Levy G, Nazareth I, et al. Relative risk of cardiovascular and cancer mortality in people with severe mental illness from the United Kingdom's General Practice Research Database. Archives of General Psychiatry 2007;**64**(2):242-49.
4. Miller B, Paschall C, Svendsen D. Mortality and medical comorbidity among patients with SMI. Psychiatric Services 2006;**57**(10):1482-87.
5. Wahlbeck K, Westman J, Nordentoft M, et al. Outcomes of Nordic mental health systems: life expectancy of patients with mental disorders. The British Journal of Psychiatry 2011;**199**(6):453-58.
6. Mental Health Statistics team HaSCIC. HES-MHMDS Data Linkage Report, Additional analysis - 2011-12, England (Experimental statistics. Leeds: Health and Social Care Information Centre, 2013.
7. Hackman AL, Goldberg RW, Brown CH, et al. Use of emergency department services for somatic reasons by people with serious mental illness. Psychiatric services 2006;**57**(4):563-66.
8. Merrick EL, Perloff J, Tompkins CP. Emergency department utilization patterns for Medicare beneficiaries with serious mental disorders. Psychiatric services 2010;**61**(6):628-31.
9. Schoenbaum M, Sutherland JM, Chappel A, et al. Twelve-Month Health Care Use and Mortality in Commercially Insured Young People With Incident Psychosis in the United States. Schizophrenia Bulletin 2017;**43**(6):1262-72.
10. Lally J, Wong YL, Shetty H, et al. Acute hospital service utilization by inpatients in psychiatric hospitals. Gen Hosp Psychiat 2015;**37**(6):577-80.
11. Reilly S, Planner C, Hann M, et al. The role of primary care in service provision for people with severe mental illness in the United Kingdom. PLoS one 2012;**7**(5):e36468.
12. World Health Organization. Quality improvement for mental health: mental health policy and service guidance package. Geneva: WHO, 2003.
13. World Health Organization. QualityRights tool kit to assess and improve quality and human rights in mental health and social care facilities. Geneva: WHO, 2012.
14. World Health Organisation. Paying for performance in health care: Implications for health system performance and accountability. In: Cashin C, Chi Y-L, Smith P, et al., eds. European Observatory on Health Systems and Policies. Maidenhead, UK: Open University Press, 2014.
15. Eijkenaar F, Emmert M, Scheppach M, et al. Effects of pay for performance in health care: a systematic review of systematic reviews. Health Policy 2013;**110**(2):115-30.
16. Flodgren G, Eccles MP, Shepperd S, et al. An overview of reviews evaluating the effectiveness of financial incentives in changing healthcare professional behaviours and patient outcomes. The Cochrane Library 2011.
17. Doran T, Maurer KA, Ryan AM. Impact of Provider Incentives on Quality and Value of Health Care. Annual Review of Public Health 2017;**38**:449-65.
18. Roland M. Linking physicians' pay to the quality of care—a major experiment in the United Kingdom. New England Journal of Medicine 2004;**351**:1448-54.
19. National collaborating Centre for Mental Health. Psychosis and Schizophrenia in Adults: Treatment and Management. NICE Clinical Guideline 178, 2014.

For peer review only - http://bmjopen.bmj.com/site/about/guidelines.xhtml

20. National Collaborating Centre for Mental Health. Bipolar disorder: The assessment and management of bipolar disorder in adults, children and young people in primary and secondary care. Leicester UK: The British Psychological Society 2014.
21. Gutacker N, Mason AR, Kendrick T, et al. Does the quality and outcomes framework reduce psychiatric admissions in people with serious mental illness? A regression analysis. Bmj Open 2015;**5**(4):e007342.
22. Purdy S, Griffin T, Salisbury C, et al. Ambulatory care sensitive conditions: terminology and disease coding need to be more specific to aid policy makers and clinicians. Public Health 2009;**123**(2):169-73.
23. Wykes TL, Lee AA, McKibbin CL, et al. Self-Efficacy and Hemoglobin A1C Among Adults With Serious Mental Illness and Type 2 Diabetes: The Roles of Cognitive Functioning and Psychiatric Symptom Severity. Psychosomatic Medicine 2016;**78**(3):263-70.
24. Chen SR, Chien YP, Kang CM, et al. Comparing self-efficacy and self-care behaviours between outpatients with comorbid schizophrenia and type 2 diabetes and outpatients with only type 2 diabetes. Journal of Psychiatric and Mental Health Nursing 2014;**21**(5):414-22.
25. Herrett E, Gallagher AM, Bhaskaran K, et al. Data resource profile: clinical practice research datalink (CPRD). International Journal of Epidemiology 2015;**44**(3):827-36.
26. Booth N. What are the Read codes? Health Libraries Review 1994;**11**(3):177-82.
27. World Health Organization, editor. *The Tenth Revision of the International Classification of Diseases and Related Health Problems (ICD-10)*. Geneva: World Health Organisation, 1992.
28. Bardsley M, Blunt I, Davies S, et al. Is secondary preventive care improving? Observational study of 10-year trends in emergency admissions for conditions amenable to ambulatory care. BMJ Open 2013;**3**(1):e002007.
29. Longman JM, Passey ME, Ewald DP, et al. Admissions for chronic ambulatory care sensitive conditions - a useful measure of potentially preventable admission? Bmc Health Serv Res 2015;**15**:472.
30. Chang KC-M, Soljak M, Lee JT, et al. Coverage of a national cardiovascular risk assessment and management programme (NHS Health Check): Retrospective database study. Preventive Medicine 2015;**78**:1-8.
31. Cox DR. Regression Models and Life-Tables. Journal of the Royal Statistical Society Series B (Methodological) 1972;**34**(2):187-220.
32. Khan NF, Perera R, Harper S, et al. Adaptation and validation of the Charlson Index for Read/OXMIS coded databases. BMC Family Practice 2010;**11**(1):1.
33. Berchet C. Emergency Care Services. 2015.
34. Broadbent M, Jarman H, Berk M. Emergency department mental health triage scales improve outcomes. Journal of Evaluation in Clinical Practice 2004;**10**(1):57-62.
35. Eric P. Slade PD, Lisa B. Dixon MD, M.P.H. ,, Sarah Semmel BA. Trends in the Duration of Emergency Department Visits, 2001–2006. Psychiatric Services 2010;**61**(9):878-84.

For peer review only - http://bmjopen.bmj.com/site/about/guidelines.xhtml

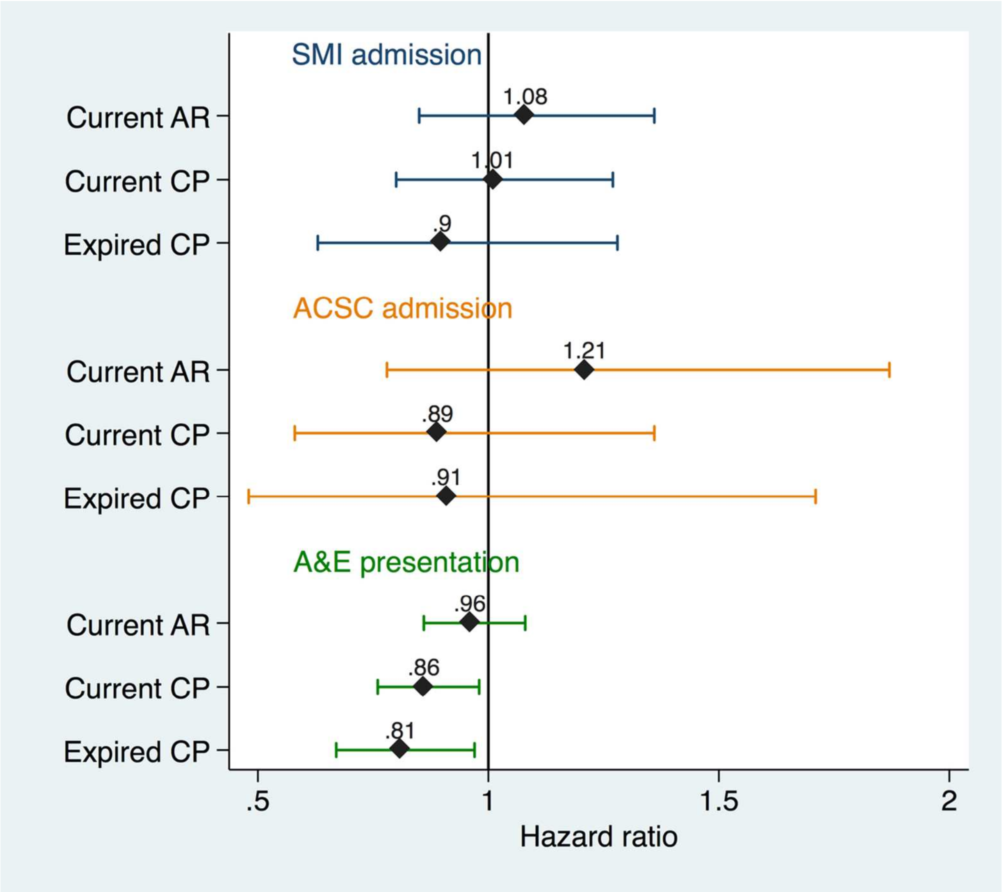

Figure 1: Association of care quality indicators with each outcome‼ ┼
CP: Care plan‼ ┼ AR: Annual review of physical health

112x100mm (300 x 300 DPI)

Supplementary Table 1: Read codes used to identify SMI diagnostic categories

Category	Read codes used to identify diagnostic category
Schizophrenia and other psychoses	E100.00 E100.11 E100000 E100100 E100200 E100300 E100400 E100500 E100z00 E101.00 E101000 E101400 E101500 E101z00 E102.00 E102000 E102100 E102500 E102z00 E103.00 E103000 E103200 E103300 E103400 E103500 E103z00 E104.00 E105.00 E105000 E105200 E105500 E105z00 E106.00 E107.00 E107.11 E107000 E107100 E107200 E107300 E107400 E107500 E107z00 E10y.00 E10y.11 E10y000 E10y100 E10yz00 E10z.00 E120.00 E121.00 E122.00 E123.00 E123.11 E12y.00 E12y000 E12yz00 E12z.00 E13..00 E13..11 E131.00 E132.00 E133.00 E133.11 E134.00 E13y.00 E13y100 E13yz00 E13z.00 E13z.11 E1z..00 E212200 Eu20.00 Eu20000 Eu20011 Eu20100 Eu20111 Eu20200 Eu20211 Eu20212 Eu20213 Eu20214 Eu20300 Eu20311 Eu20400 Eu20500 Eu20511 Eu20600 Eu20y00 Eu20y12 Eu20y13 Eu20z00 Eu21.00 Eu21.11 Eu21.12 Eu21.13 Eu21.14 Eu21.15 Eu21.16 Eu21.17 Eu21.18 Eu22.00 Eu22000 Eu22011 Eu22012 Eu22013 Eu22014 Eu22015 Eu22100 Eu22111 Eu22200 Eu22300 Eu22y00 Eu22y11 Eu22y12 Eu22y13 Eu22z00 Eu23.00 Eu23000 Eu23011 Eu23012 Eu23100 Eu23112 Eu23200 Eu23211 Eu23212 Eu23214 Eu23300 Eu23312 Eu23y00 Eu23z00 Eu23z11 Eu23z12 Eu24.00 Eu24.12 Eu24.13 Eu25.00 Eu25000 Eu25011 Eu25012 Eu25100 Eu25111 Eu25112 Eu25200 Eu25212 Eu25y00 Eu25z00 Eu25z11 Eu26.00 Eu2y.00 Eu2y.11 Eu2z.00 Eu2z.11 Eu44.14
Bipolar disorder and affective psychoses	E11..00 E11..12 E110.00 E110.11 E110000 E110100 E110200 E110300 E110400 E110600 E110z00 E111.00 E111000 E111100 E111200 E111300 E111400 E111500 E111600 E111z00 E112400 E113400 E114.00 E114.11 E114000 E114100 E114200 E114300 E114400 E114500 E114600 E114z00 E115.00 E115.11 E115000 E115100 E115200 E115300 E115400 E115500 E115600 E115z00 E116.00 E116000 E116100 E116200 E116300 E116400 E116500 E116600 E116z00 E117.00 E117000 E117100 E117200 E117300 E117400 E117500 E117600 E117z00 E11y.00 E11y000 E11y100 E11y300 E11yz00 E11z.00 E11z000 E11zz00 E130.00 E130.11 E13y000 Eu30.00 Eu30.11 Eu30000 Eu30100 Eu30200 Eu30211 Eu30212 Eu30y00 Eu30z00 Eu30z11 Eu31.00 Eu31.11 Eu31.12 Eu31.13 Eu31000 Eu31100 Eu31200 Eu31300 Eu31400 Eu31500 Eu31600 Eu31700 Eu31800 Eu31900 Eu31911 Eu31y00 Eu31y11 Eu31y12 Eu31z00 Eu32300 Eu32311 Eu32312 Eu32313 Eu32314 Eu32800 Eu33213 Eu33300 Eu33311 Eu33312 Eu33313 Eu33314 Eu33315 Eu33316 Eu3z.11

For peer review only

Supplementary Figure 1 shows the rate of care indicators recorded by GP practices per patient-year for each financial year in our sample. This rate is the number of times an indicator is recorded in that year across all patients, divided by the number of patient-years of observation in that financial year (which takes into account partial years for patients who were not observed for the full financial year). For example, in 2007/08 the rate of annual reviews (composite measure) recorded was 1.06 per patient-year.

The CP indicator shows a decline over the study period until 2012/13, when there was an increase in the threshold of achievement required to attain the maximum payment. The original AR indicator shows a marked drop-off after it ceased to be incentivized in 2010/11, while the aggregate indicator composed of the ‘health risk’ checks shows less marked change but a small increase after the specific components began to be incentivized in 2011/12. The composite of these two AR indicators shows little change in response to the QOF rule changes.

Supplementary Figure 1: Rate of care indicators per patient-year

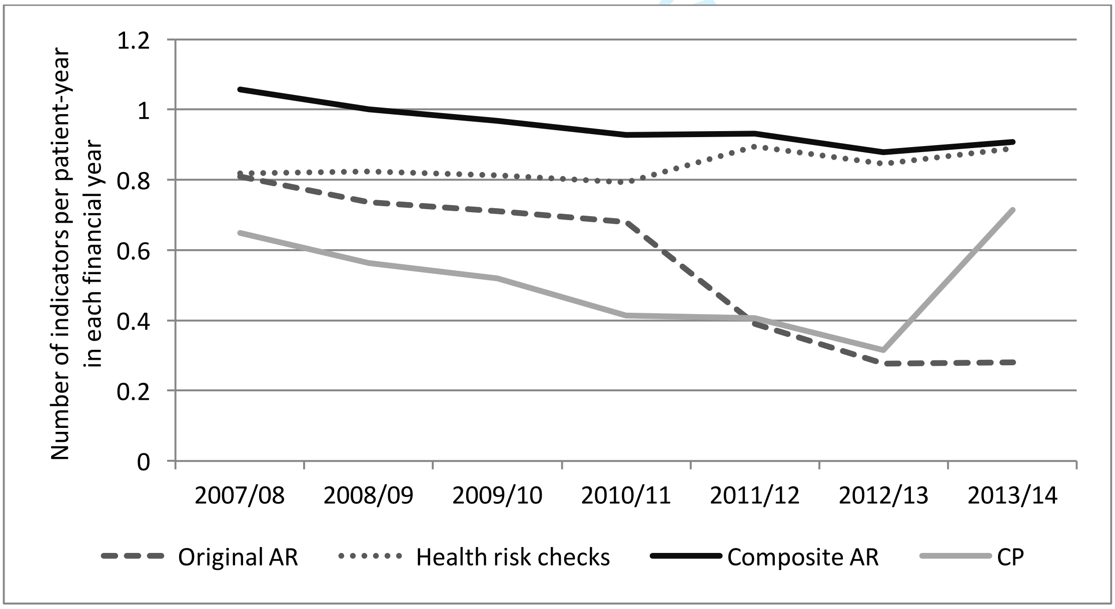

For peer review

Supplementary Table 2: Prevalence of Charlson Index comorbidities at time of SMI diagnosis

Comorbidities at time of SMI diagnosis	Proportion of sample (%)
Cancer	7.31
Cerebrovascular disease	5.08
COPD	19.72
CHD	2.12
Dementia	3.06
Diabetes	7.37
Hemiplegia	0.60
Liver disease	0.78
Myocardial infarction	2.68
Peptic ulcer	2.54
Peripheral vascular disease	1.96
Renal disease	7.60
Rheumatological disease	2.56

For peer review only

Supplementary Table 3: Main model - full results with A&E presentation as the outcome

	Main model HR	(95% CI)
Current annual review	0.963	(0.857-1.081)
Current care plan	0.866*	(0.766-0.979)
Expired care plan	0.808*	(0.671-0.972)
Age at diagnosis		
18-35 (base)		
36-45	0.807*	(0.659-0.989)
46-55	0.620***	(0.508-0.757)
56-65	0.584***	(0.460-0.740)
>=66	1.099	(0.918-1.315)
Male	1.015	(0.859-1.199)
Age at diagnosis*Male		
*18-35*Male (base)*		
36-45*Male	0.921	(0.695-1.220)
46-55*Male	1.215	(0.913-1.617)
56-65*Male	1.436*	(1.040-1.983)
≥66*Male	0.939	(0.729-1.211)
SMI diagnosis group		
Bipolar disorder or affective psychosis (base)		
Schizophrenia or other psychosis	1.102	(0.993-1.223)
Both	1.119	(0.895-1.399)
IMD		
1st quintile (base)		
2nd quintile	0.955	(0.805-1.134)
3rd quintile	1.101	(0.919-1.320)
4th quintile	1.178	(0.978-1.419)
5th quintile	1.196	(0.984-1.452)
Ethnicity white	1.280***	(1.149-1.424)
Number of admissions within 12 months prior to diagnosis		
0 (base)		
1	1.173**	(1.050-1.312)
2	1.190*	(1.028-1.377)
3 or more	1.501***	(1.269-1.775)
Number of primary care contacts within 12 months prior to diagnosis		
<5 (base)		
5-9	1.221**	(1.056-1.410)
10-14	1.337***	(1.165-1.533)
15-19	1.716***	(1.460-2.018)

For peer review only

	Main model HR	(95% CI)
>=20	1.865***	(1.594-2.182)
Current or ex-smoker	1.105	(0.986-1.239)
Current or ex-alcohol user	0.978	(0.882-1.083)
Number of Charlson Index comorbidities	1.111***	(1.052-1.174)
Diagnosis of depression	1.006	(0.905-1.118)
Year of diagnosis		
2007 (base)		
2008	1.263*	(1.041-1.533)
2009	1.382***	(1.149-1.664)
2010	1.645***	(1.356-1.994)
2011	1.551***	(1.244-1.933)
2012	1.387**	(1.106-1.740)
2013	1.266	(0.991-1.617)
2014	0.972	(0.558-1.694)
Practice list size	1.000	(1.000-1.000)
Number of patients	4,446	

*p<0.05, **p<0.01, ***p<0.001

For peer review only

BMJ Open

Do care plans and annual reviews of physical health influence unplanned hospital utilisation for people with serious mental illness? Analysis of linked longitudinal primary and secondary healthcare records

Journal:	*BMJ Open*
Manuscript ID	bmjopen-2018-023135.R1
Article Type:	Research
Date Submitted by the Author:	08-Aug-2018
Complete List of Authors:	Ride, Jemimah; University of York Centre for Health Economics,<br>Kasteridis, Panagiotis; University of York Centre for Health Economics<br>Gutacker, Nils; University of York, Centre for Health Economics<br>Kronenberg, Christoph; University Duisburg-Essen, CINCH ; Leibniz Science Campus Ruhr<br>Doran, Tim; University of York, Health Sciences<br>Mason, Anne; University of York, Centre for Health Economics<br>Rice, Nigel; University of York Centre for Health Economics<br>Gravelle, Hugh; University of York Centre for Health Economics<br>Goddard, Maria; University of York, Centre for Health Economics<br>Kendrick, Tony; University of Southampton, Primary Care and Population Sciences<br>Siddiqi, Najma; University of York, Psychiatry, Hull York Medical School, York and Bradford District Care NHS Foundation Trust, Bradford, UK<br>Gilbody, Simon; The University of York, Department of Health Sciences<br>Dare, Ceri; McPin Foundation,<br>Aylott, Lauren<br>Williams, Rachael; Clinical Practice Research Datalink,<br>Jacobs, Rowena; University of York, Centre for Health Economics
**Primary Subject Heading**:	Health economics
Secondary Subject Heading:	Health policy, Mental health, General practice / Family practice, Health services research
Keywords:	Schizophrenia & psychotic disorders < PSYCHIATRY, PRIMARY CARE, Quality in health care < HEALTH SERVICES ADMINISTRATION & MANAGEMENT, Data linkage, Hospital utilisation

SCHOLARONE™
Manuscripts

Do care plans and annual reviews of physical health influence unplanned hospital utilisation for people with serious mental illness? Analysis of linked longitudinal primary and secondary healthcare records

Authors:

Jemimah Ride (corresponding author): Centre for Health Economics, Alcuin 'A' Block, University of York, Heslington, YO10 5DD, United Kingdom. jemimah.ride@york.ac.uk (+44) 01904 321 459

Panagiotis Kasteridis[a] (panos.kasteridis@york.ac.uk); Nils Gutacker[a] (nils.gutacker@york.ac.uk); Christoph Kronenberg[b] (christoph.kronenberg@uni-due.de); Tim Doran[c] (tim.doran@york.ac.uk); Anne Mason[a] (anne.mason@york.ac.uk); Nigel Rice[a] (nigel.rice@york.ac.uk); Hugh Gravelle[a] (hugh.gravelle@york.ac.uk); Maria Goddard[a] (maria.goddard@york.ac.uk); Tony Kendrick[d] (A.R.Kendrick@soton.ac.uk); Najma Siddiqi[c] (najma.siddiqi@york.ac.uk); Simon Gilbody[c] (simon.gilbody@york.ac.uk); Ceri R Dare[e] (ceri.owen@gmail.com); Lauren Aylott[e] (lmeaylott@gmail.com); Rachael Williams[f] (Rachael.Williams@mhra.gov.uk); Rowena Jacobs[a] (rowena.jacobs@york.ac.uk)

a: Centre for Health Economics, University of York

b: CINCH, University Duisburg-Essen, Essen, Germany; Leibniz Science Campus Ruhr, Essen, Germany; RWI – Leibniz-Institute for Economic Research, Essen, Germany

c: Department of Health Sciences, University of York

d: Primary Care and Population Sciences, University of Southampton

e: Service user

f: Clinical Practice Research Datalink

Word count: 3,919

For peer review only - http://bmjopen.bmj.com/site/about/guidelines.xhtml

ABSTRACT

Objective: To investigate whether two primary care activities that are framed as indicators of primary care quality (comprehensive care plans and annual reviews of physical health) influence unplanned utilisation of hospital services for people with serious mental illness (SMI).

Design & setting: Retrospective observational cohort study using linked primary care and hospital records (Hospital Episode Statistics) for 5,158 patients diagnosed with SMI between April 2006 and March 2014, who attended 213 primary care practices in England that contribute to the Clinical Practice Research Datalink GOLD database. Cox survival models were used to estimate the associations between two primary care quality indicators (care plans and annual reviews of physical health) and the hazards of three types of unplanned hospital utilisation: presentation to Accident & Emergency departments (A&E), admission for SMI, and admission for ambulatory care sensitive conditions (ACSC).

Results: Risk of A&E presentation was 14% lower (hazard ratio (HR) 0.86, 95% CI 0.76-0.98) and risk of admission to hospital for ACSC was 23% lower (HR 0.77, 95%CI 0.60-0.99) for patients with a care plan documented in the previous year compared to those without a care plan. Risk of A&E presentation was 19% lower for those who had a care plan documented earlier but not updated in the previous year (HR: 0.81, 95% CI 0.67-0.97) compared to those without a care plan. Risks of hospital admission for SMI were not associated with care plans, and none of the outcomes were associated with annual reviews.

Conclusions: Care plans documented in primary care for people with SMI are associated with reduced risk of A&E attendance and reduced risk of unplanned admission to hospital for physical health problems, but not with risk of admission for

For peer review only - http://bmjopen.bmj.com/site/about/guidelines.xhtml

mental health problems. Annual reviews of physical health are not associated with risk of unplanned hospital utilisation.

For peer review only

For peer review only - http://bmjopen.bmj.com/site/about/guidelines.xhtml

Strengths and limitations of this study

- Innovative use of linked individual-level patient data from primary care, inpatient admissions and A&E, allowing the sequence of care indicators and unplanned hospital utilisation to be identified.
- Contributes to a limited evidence base on the association of patient outcomes with the types of care incentivised under a national primary care physician incentive scheme.
- Due to the observational nature of the data, no information was available on the circumstances leading to documentation of care quality indicators, or their quality or content.
- Partial capacity to account for patient health status and factors driving utilisation of primary and secondary care.

Keywords

Schizophrenia & psychotic disorders; primary care; quality in health care; data linkage; hospital utilisation.

Funding statement

This project was funded by the National Institute for Health Research HS&DR programme (project number 13/54/40). The views expressed are those of the author(s) and not necessarily those of the NHS, the NIHR or the Department of Health.

Competing interests

No competing interests.

For peer review only - http://bmjopen.bmj.com/site/about/guidelines.xhtml

INTRODUCTION

Serious mental illness (SMI) comprises a set of conditions including schizophrenia and bipolar disorder with profound impacts on the wellbeing of patients and high costs to society. It is linked with a high disease burden[1 2], poor health outcomes, high treatment costs, and lower life expectancy, primarily attributed to preventable physical causes[3-6]. Patients with SMI have high rates of A&E attendance and hospital admission, for both physical and mental health problems[7-10].

In the UK, while specialist mental health services are important to many patients' care, general practice provides the majority of care for patients with SMI[11]. High quality primary care therefore has the potential to improve the management, health and wellbeing of patients with SMI, and can help meet the aims of policymakers seeking to improve population wellbeing and contain costs[12 13].

Several countries have adopted incentive programs to improve the quality and value of healthcare[14], despite mixed evidence of their effectiveness[15-17]. England introduced a voluntary scheme for primary care in 2004, the Quality and Outcomes Framework (QOF), offering GPs incentives to meet quality targets for patients with several chronic conditions, including SMI[18]. Two key SMI indicators in the QOF, which apply to all registered patients with SMI, promote proactive management of physical and mental health: the proportion of patients on the practice SMI register who had an in-date comprehensive care plan, and the proportion who had an annual physical health review.

For peer review only - http://bmjopen.bmj.com/site/about/guidelines.xhtml

There is little evidence on whether these incentivised activities improve patients' mental or physical health, modify patterns of healthcare utilisation, or reduce costs. They derive from NICE guideline recommendations for primary care management of SMI, which are based on consensus in the absence of robust evidence[19 20].

A previous study using data aggregated to practice level found that higher rates of SMI admission were associated with higher achievement on the annual physical review indicator, but not the care plan indicator[21]. However, that study could not ascertain which individuals within a practice received care or whether the care preceded or followed admissions.

As the QOF indicators are regularly reviewed and revised, new evidence on their potential impacts is useful for decision-makers. Our study contributes such evidence, examining whether care plans and annual reviews are associated with utilisation of unplanned secondary care for patients with SMI, by analysing linked primary care and hospital records. We hypothesise that care plans and annual reviews may modify risk of unplanned hospital utilisation, which is potentially preventable by high-quality primary care[22]. Patients with SMI are less likely to report health problems than their peers due to self-neglect, impaired motivation, and social withdrawal[23 24]. Proactive care by GPs may help to identify and manage physical and mental health problems without the need for hospital care.

For peer review only

METHODS

Study design

In this retrospective observational cohort study using linked primary care and hospital data, we investigate the relationship between having a care plan and/or an annual physical health review, and time from diagnosis of SMI to unplanned hospital utilisation.

Data sources and linkage

The main data source is the Clinical Practice Research Datalink (CPRD) GOLD, which holds linked individual-level anonymized primary care records from participating general practices in the UK. These data are representative of the English population with respect to age and gender, but not region. For example, the north-east of England is under-sampled relative to areas in the west and south[25]. The CPRD data service provided information on all patients eligible for linkage and registered with a participating practice in England, and with a diagnosis of SMI documented on or before 31 March 2014 recorded in clinical notes or referral records, using the event date entered by the GP. The sample was limited to patients whose records met an acceptable standard based on recording of registration, clinical events, and demographic details, at practices deemed up to standard according to a CPRD algorithm[25]. Most of the primary care information is recorded using a hierarchical coding system known as 'Read codes'[26], which we use to identify records of SMI diagnosis, care quality indicators, and morbidity profiles. CPRD records were linked to Hospital Episode Statistics (HES), which comprise detailed records for all NHS patients admitted to hospital and presentations to A&E in England. CPRD provided deterministically linked admission and A&E data for all patients included in the study.

For peer review only - http://bmjopen.bmj.com/site/about/guidelines.xhtml

To preserve anonymity, all linkages were carried out under the CPRD routine linkage scheme.

Inclusion and exclusion criteria

Our sample covers eight financial years, from 1 April 2006 to 31 March 2014. The care plan indicator was introduced in 2006/07 and the last year of HES data available to us at the start of the study was 2013/14. The sample comprises patients aged 18 years and over, whose earliest recorded diagnosis of SMI in primary care was after 31 March 2006, and who were registered with the same practice for at least 365 days before that diagnosis. A&E data are only available from 2007/08, so the analysis of A&E presentations is limited to patients with a first diagnosis after 31 March 2007. The restriction to newly diagnosed patients excludes those whose unobserved past care or events could influence their subsequent care.

Outcomes

We analyse three outcomes taken from HES data: time to i) presentations to Accident and Emergency departments (A&E), ii) unplanned hospital admission for SMI, and iii) unplanned admissions for ambulatory care sensitive conditions (ACSCs). Unplanned admissions, based on the HES admission method codes for emergency admissions, were classified by International Classification of Diseases (ICD-10) codes. For SMI admissions this included admissions with primary diagnosis field code categories F20-F31[27]. For ACSC, the codes were those defined by Bardsley, et al.[28] (see Supplementary Table 1 for the list of conditions included). A&E presentations of any cause (mental or physical health) were included.

For peer review only - http://bmjopen.bmj.com/site/about/guidelines.xhtml

Care quality indicators

Primary care quality indicators were taken from CPRD data, and capture care plans and annual reviews of physical health based on Read codes specified under the QOF to identify those two indicators. A good quality care plan documents the patient's current health and social care needs, and how these will be addressed, in agreement with patients and caregivers. It should specify arrangements with secondary care services (where applicable), and recommendations in case of relapse, including the patient's care preferences and goals[19 20]. The QOF incentivises GPs to document care plans annually, so we analyse 'current' care plans, those recorded in the last twelve months. However, we hypothesise that an older care plan may still influence patients' health service utilisation, and therefore also analyse 'expired' care plans, those recorded more than a year ago. Conversely, only 'current' annual reviews (recorded within the last twelve months) are included in the analysis, since expired monitoring of physical health is unlikely to have ongoing benefits. The choice of the twelve-month window to determine expiration status is based on the QOF guidance that a care plan or annual review should be reviewed annually[3].

From 2006/7 to 2010/11 the QOF annual review indicator entailed the patient being given appropriate health promotion and prevention advice. In 2011/12, this broad indicator was split into more specific indicators: a record of alcohol consumption, checks of blood pressure, body mass index (BMI), blood glucose or glycated haemoglobin, and ratio of total to high-density lipoprotein cholesterol, and, where appropriate, cervical screening. In order to explore annual reviews for the full period of our analysis, we formulate an aggregate indicator. This signifies that the patient had at least three of four 'health risk' checks (blood pressure, BMI, cholesterol, and

For peer review only - http://bmjopen.bmj.com/site/about/guidelines.xhtml

glucose) documented within a three month period[29]. The date of the aggregate indicator is the date of the final check. In our analysis, patients are considered to have a 'current' annual review for twelve months after either an annual review (using the original Read coding), or from the date of this aggregate indicator.

The analysis includes three time-varying care quality indicator variables. For annual reviews, the base case is no annual review of physical health within the last twelve months. For care plans the base case is never having had a care plan. Two binary variables indicate whether a care plan or annual review has been recorded within the last twelve months ('current care plan' and 'current annual review'). If a patient receives a further care plan or annual review within the twelve-month window, the 'current' period is extended accordingly. A third variable indicates that the patient received a care plan more than twelve months ago ('expired care plan') but none in the last twelve months (mutually exclusive with 'current care plan').

Control variables

Using primary care data, we control for patients' age, gender, ethnicity, deprivation profile of their area of residence[30], year of SMI diagnosis, Charlson comorbidities[31], diagnosis of depression, history of smoking, and number of primary care attendances in the year prior to diagnosis. Their diagnosis of SMI was classified as "schizophrenia and other psychoses", "bipolar disorder and affective psychoses", or both, if the patient had both recorded. (See Supplementary Table 2 for a list of codes.) From HES data we capture number of hospital admissions in the year prior to diagnosis.

For peer review only - http://bmjopen.bmj.com/site/about/guidelines.xhtml

Statistical analysis

We estimate Cox survival models[32], examining duration from SMI diagnosis to each of the three outcomes separately: first A&E presentation, first SMI admission, and first ACSC admission. We follow each individual until the outcome of interest or until censoring. Censoring can occur because i) a patient dies, ii) registration with the practice ends, or iii) the study period ends (i.e. the patient is still registered on 31 March 2014).

In the Cox model the hazard of the outcome occurring is a function of the baseline hazard, the care indicators, and patient demographic and clinical characteristics. In the main model, we assume patient characteristics to be fixed at baseline, while the care indicators may vary over time. (Further details available in supplementary material.) We stratify on practice, so that the baseline hazard varies across practices, to allow for unobserved differences in patient populations and practice characteristics, and standard errors are adjusted for clustering at practice level to account for within-practice correlation. Coefficient estimates are presented as hazard ratios (HRs) with associated 95% confidence intervals (CIs), where values greater than 1 indicate an increase in the hazard of the outcome associated with a unit change in the explanatory variable, and vice versa for a HR below 1. All analyses were performed in Stata 14 (StataCorp LP, College Station, TX, US).

Sensitivity analyses

We undertake five tests of the sensitivity of our findings to alternate specifications of the model. First, we test the robustness of the annual review variable, which in our main analysis is the composite indicator constructed from both the QOF-specified

For peer review only - http://bmjopen.bmj.com/site/about/guidelines.xhtml

Read codes and the specific health checks that contribute to such reviews. We explore the impact of restricting this to the QOF-specified Read codes, which necessitates limiting the observation period to 2006/07 to 2010/11 (or 2007/08 to 2010/11 for the A&E outcome). Second, we assume the indicators expire after 15 months instead of 12 months. Third, instead of fixing the number of Charlson comorbidities at baseline, we allow this to increase over the period of observation if patients are diagnosed with new comorbidities. We lastly apply two alternate specifications of practice-level characteristics, instead of stratifying on practice. The first assumes no practice-level differences in baseline hazard, but includes practice characteristics (rurality and distance from closest acute hospital and inpatient psychiatric unit) as covariates. The second includes practice fixed effects as explanatory variables. Both models adjust the standard errors for clustering at the practice level as before.

Patient involvement

Two co-authors on the multidisciplinary team responsible for this study have lived experience of SMI. They contributed to the design of the research questions and methodological approach, interpretation of the findings, and writing the paper.

RESULTS

Study population

The full sample consists of 5,158 newly diagnosed patients with SMI from 213 practices in England with a total of 14,376 person-years observed before censoring (for A&E analysis 4,446 patients and a total of 10,952 person-years). On average patients were observed for 2.79 years. Most (67%) were still observed at the end of the study period, 23% exited because their registration with the practice ended during the study period, and 10% died. Table 1 presents the number of individuals with each

For peer review only - http://bmjopen.bmj.com/site/about/guidelines.xhtml

characteristic, and the contribution of those with each characteristic to the total time observed. (Equivalent tables are presented for each analysis sample in Supplementary Tables 3-5). There were more people per year diagnosed in later years of the study period, but each was observed for a shorter period, and therefore had a shorter window of opportunity to experience both the care quality indicators and the outcomes. Similarly, those aged over 65 made up 25% of the sample but only contributed 18% of the total time observed because they had a shorter than average period of observation (2.01 years). The median age at diagnosis in the sample was 46, and the most common physical comorbidities were respiratory disease, renal disease, cancer, and diabetes (further details available in Supplementary Table 6).

For peer review only

For peer review only - http://bmjopen.bmj.com/site/about/guidelines.xhtml

Table 1. Population characteristics: number and proportion of individuals in the sample, total person-years in the sample, and proportion of total person-years

	Individuals (% of total individuals)	Mean person-years (% of total person-years)
Total sample	5,158	14,736
Age at diagnosis		
18-35	1,540 (30%)	4,388 (30%)
36-45	1,008 (20%)	3,206 (22%)
46-55	736 (14%)	2,356 (16%)
56-65	561 (11%)	1,780 (12%)
>65	1,313 (25%)	2,646 (18%)
Index of multiple deprivation		
Quintile 1 (least deprived)	922 (18%)	2,535 (18%)
Quintile 2	980 (19%)	2,709 (19%)
Quintile 3	951 (18%)	2,613 (18%)
Quintile 4	1,150 (22%)	3,232 (22%)
Quintile 5 (most deprived)	1,155 (23%)	3,286 (23%)
Gender		
Male	2,430 (47%)	6,767 (47%)
Female	2,728 (53%)	7,609 (53%)
Ethnicity		
White	3,791 (73%)	10,546 (73%)
Black & minority ethnicities	1,367 (27%)	3,830 (27%)
Number of primary care contacts in year preceding diagnosis		
0-4	1,143 (22%)	3,393 (24%)
5-9	1,341 (26%)	4,032 (28%)
10-14	946 (18%)	2,598 (18%)
15-19	618 (12%)	1,619 (11%)
>=20	1,110 (22%)	2,735 (19%)
Number of hospital admissions in year preceding diagnosis		
0	2,886 (56%)	8,515 (59%)
1	1,250 (24%)	3,384 (23%)
2	544 (11%)	1,385 (10%)
3	478 (9%)	1,092 (8%)
Number of Charlson Index comorbidities at time of diagnosis		
0	3,047 (59%)	9,049 (63%)
1	1,384 (27%)	3,908 (27%)
2	446 (9%)	948 (7%)
3 or more	281 (5%)	471 (3%)
Comorbid depression at time of diagnosis		
History of depression	3,358 (65%)	9,648 (67%)

For peer review only - http://bmjopen.bmj.com/site/about/guidelines.xhtml

No recorded history of depression	1,800 (35%)	4,728 (33%)
Smoking status		
Current or ex-smoker	3,960 (77%)	11,059 (77%)
No recorded history of smoking	1,198 (23%)	3,317 (23%)
SMI diagnostic group		
Schizophrenia & other psychoses	2,884 (56%)	7,292 (51%)
Bipolar disorder & affective psychoses	2,078 (40%)	6,314 (44%)
Both	196 (4%)	770 (5%)
Financial year of diagnosis		
2006/07	617 (12%)	3,205 (22%)
2007/08	523 (10%)	2,381 (17%)
2008/09	573 (11%)	2,269 (16%)
2009/10	619 (12)	2,084 (14%)
2010/11	607 (12%)	1,611 (11%)
2011/12	734 (14%)	1,528 (11%)
2012/13	732 (14%)	969 (7%)
2013/14	753 (15%)	330 (2%)
Care plans (varying over time)^		
Current care plan (in last 12 mo.)		5,692 (40%)
Expired care plan (more than 12 mo. ago)		4,004 (28%)
No care plan at all		4,680 (32%)
Annual reviews (varying over time)^		
Current annual review (in last 12 mo.)		8,579 (60%)
No annual review in last 12mo		5,797 (40%)

Patient characteristics are fixed at baseline (date of diagnosis) except for care quality indicators, which vary over the study period.
^The three care plans categories are mutually exclusive (as are the two annual review categories) so each person's total time observed is the sum of these categories

For peer review only

Overall, 69% of the sample had a current care plan at least once, contributing 40% of the total time observed, while 72% of the sample had a current annual review at least once, contributing 60% of the total time observed, and 42% of the sample had an expired care plan at least once, contributing 28% of the total time observed. More detail on the annual rates of care plan and annual review indicators is available in supplementary material (Supplementary Figure 1).

Outcomes

Summary statistics for each of the three outcome variables are presented in Table 2. During the period of observation, 50% had an A&E presentation, 11% had an SMI admission, and 10% had an ACSC admission.

For peer review only

Table 2. Descriptive statistics for survival analysis, by outcome

Outcome	A&E presentation	SMI admission	ACSC admission
N individuals	4,446	5,158	5,158
Mean years observed	1.50	2.47	2.56
N with outcome (% of sample)	2,213 (50%)	562 (11%)	528 (10%)
Mean years observed to outcome*	1.08	1.20	1.68
N (% of events) with outcome occurring:			
within 12 months of CP (current CP)	756 (36%)	200 (35%)	171 (32%)
>12 months since CP (expired CP)	250 (11%)	77 (14%)	109 (21%)
with no CP	1,207 (54%)	285 (51%)	248 (47%)
within 12 months of AR (current AR)	1,024 (46%)	263 (47%)	301 (57%)
with no current annual in last 12 months	1,189 (54%)	299 (53%)	227 (43%)

The period of observation is shorter in the outcome-specific samples than the full study observation period because the patient exits observation once the first outcome event occurs.
*Mean years observed from diagnosis to outcome for those who experienced the outcome.
CP: Care plan. AR: Annual review of physical health.
A&E presentation: Presentation of any cause to the Accident & Emergency department
SMI admission: Inpatient admission with a primary diagnosis of serious mental illness (ICD-10 codes F20-F31)
ACSC admission: Inpatient admission with a diagnosis of an ambulatory care-sensitive condition (see Supplementary Table 1 for a list of conditions)

For peer review only

Results of survival analysis

Results showing the association of the three key explanatory variables with hazard (HR) of each of the outcomes are presented in Figure 1. HRs for control variables are presented in Supplementary Table 7.

[Figure 1 about here]

The hazard of A&E presentation is 14% lower for patients who have a current care plan (HR 0.86, 95% CI 0.76-0.98) and 19% lower for those with an expired care plan (HR 0.81, 95% CI 0.67-0.97), both compared with those who have had no care plan at all. There is no statistically significant association with annual reviews of physical health (HR 0.96, 95% CI 0.86-1.08). The hazard of first ACSC admission after SMI diagnosis is 23% lower for those with a current care plan (HR 0.77, 95% CI 0.60-0.99), but there is no statistically significant association with expired care plans or annual reviews. Neither indicator shows a statistically significant association with the hazard of SMI admission.

Results of sensitivity testing (detailed in Supplementary Tables 8-10) show no statistically significant association between annual reviews of physical health and any of the outcomes under all assumptions tested. The association between care plans and A&E presentations remained essentially unchanged, except that the association with current care plans became non-significant (as confidence intervals widened) when the analysis was limited to 2007/08 – 2010/11, and similarly with expired care plans when the indicators were assumed to be effective (current) for 15 months instead of 12. There remained no statistically significant association between the indicators and

For peer review only - http://bmjopen.bmj.com/site/about/guidelines.xhtml

SMI admissions under the varying assumptions. The association between ACSC admission and current care plans was less robust to changing assumptions, losing statistical significance under most alternate specifications and changing signs when the period of analysis was limited to 2007/08 – 2010/11.

DISCUSSION

Principal findings

We find that, among patients with SMI, those with a care plan documented in primary care in the last 12 months have a 14% lower hazard of A&E presentation than those without a care plan, and a 23% lower hazard of admission to hospital for ACSC (the types of condition thought to be amenable to primary care). Those with a care plan documented more than 12 months ago also had a lower hazard of A&E presentation (19% lower than those with no care plan). We find no such association between annual reviews of physical health and the hazard of any of the outcomes, nor between care plans and admissions for SMI.

Strengths and weaknesses

Our study makes a number of important contributions to the evidence base, being (to our knowledge) the first to use linked patient-level data to investigate the relationship between incentivized primary care quality and hospital care for people with SMI in the English NHS. By linking datasets, we track individual patients across primary care and hospital settings over a number of years, and determine whether the primary care indicator precedes the hospital utilisation. Our survival analysis exploits this, providing more robust estimates of the association than would be possible with aggregate data.

For peer review only - http://bmjopen.bmj.com/site/about/guidelines.xhtml

There are a number of limitations to our study. The measures of health status and healthcare utilisation prior to diagnosis of SMI may not fully depict the complexities of health status, including severity of SMI, and this may confound the results. However, our findings were robust to whether the number of comorbidities was fixed at baseline or allowed to vary over the period of observation. The outcomes we analyse are measures of hospital utilisation, which are imperfect proxies for health outcomes. Our finding of a negative association between care plans and A&E presentations or ACSC admissions may reflect the presence of unobserved factors that could contribute to the observed association without implying causality. We lack information on the clinical circumstances leading to a care plan or annual review being recorded, and on its content, quality and appropriateness. In the period preceding hospital attendance, it is possible that care planning could be superseded by acute management of physical or mental health conditions. Patients who have less insight or lower levels of self-care may be less likely to seek regular primary care, and therefore less likely to have care plans documented, but may also be more likely to seek A&E care[33 34]. Conversely, our findings could underestimate any real effect of care plans on A&E presentations if other unobserved factors contribute to a (non-causal) positive association, such as care plans being triggered by patients attending their GP for problems that will eventually need hospital care.

We restricted the sample to patients registered with the same practice for the year preceding diagnosis in order to analyse a group of patients at a similar clinical stage, and allow us to include measures of historical utilization and medical history. However, the restriction may have excluded individuals with more severe SMI if this

For peer review only - http://bmjopen.bmj.com/site/about/guidelines.xhtml

led them to move practice. In addition, the sample may include some patients who were not newly diagnosed, if their diagnostic information had not been transferred to a new practice, or to a new electronic recording system.

The collection of HES A&E commenced on an experimental basis in April 2007, and captured 62% of national A&E attendances in the first financial year. The experimental label was lifted by April 2013 after capture had increased to over 80%. However, there is no reason to consider that the capture of attendances is related to whether a patient had a care plan or physical review, meaning our comparative analysis should not be adversely impacted.

Comparison with other studies

There is limited, mixed evidence about the relationship between objective measures of primary care quality and A&E attendance. Baker, et al.[35] found that higher performance on the QOF overall was not a predictor of area-level A&E attendances, while subjective assessment of higher primary care quality has been found to be associated with lower utilisation of A&E[36 37]. Using practice-level data, Gutacker, et al.[21] found that higher achievement on the annual review indicator was associated with a higher rate of SMI admissions. One possible explanation was that patients had QOF indicators documented after a hospital admission. Our analysis, which captures the sequence of events and finds no association between annual reviews and hazard of SMI admission, supports that explanation. Harrison, et al.[38] found, using aggregate data, that the introduction of the QOF was associated with a decrease in unplanned admissions for incentivised ACSCs, consistent with our finding that care plans were associated with a reduced risk of ACSC admissions. Wilson, et al.[39] found, using

For peer review only - http://bmjopen.bmj.com/site/about/guidelines.xhtml

individual-level data, that while introduction of QOF incentives increased detection of cardiovascular risk factors in patients with SMI, there was not necessarily any change in management. This is consistent with our finding no evidence of improved health outcomes (or their proxies, unplanned hospital utilisation) associated with annual reviews of physical health.

Interpretation of results and implications for clinicians and policymakers

The association of care plans with reduced hazard of A&E presentations and ACSC admissions might suggest that care plans help patients avoid hospital for conditions that do not require hospital care. The documentation of patients' current health status, early warning signs, triggers, social support needs, co-ordination arrangements with secondary care, and preferred course of action in the event of a clinical relapse could improve the management of the patient's health overall and prevent deterioration, reducing the need for urgent care represented by ACSC admissions and A&E presentations. It may also direct patients into appropriate services during periods of deterioration, and thereby avert hospital use.

Increasing demand for A&E care, especially for problems that could be managed elsewhere, is a policy focus in the UK NHS and in other health care systems[40]. Patients with SMI, in particular, can require high resource input when attending A&E[41 42]. The potential for care planning by GPs to reduce A&E attendances in this patient population could therefore be an important finding for policymakers, suggesting that continued incentivisation of this activity in primary care may help reduce demand on secondary care services.

For peer review only - http://bmjopen.bmj.com/site/about/guidelines.xhtml

The associations of both ‘current’ and ‘expired’ care plans with lower hazard of A&E attendance suggest that an older care plan can still be beneficial. However, the finding that when care plans are considered effective for 15 months rather than 12 months, only ‘current’ care plans are associated with reduced A&E attendance suggests that there are limits to the duration of impact. The lack of association between annual reviews of physical health and unplanned hospital care may suggest that effectiveness of a GP in managing a patient’s health, and preventing the need for A&E or hospital care, may be unaffected by whether or not annual reviews or health checks are recorded.

Unanswered questions and future research

This study did not account for care provided by patients’ key workers or a crisis resolution and home treatment team, which may provide an alternative to both hospital admission and primary care. The modelling approach does adjust for differences across practices, which may help control for some of these local effects. To address this more adequately, the Mental Health Services Dataset, which can now be linked to CPRD primary care data, could be used to cover mental health care delivered in the community.

Conclusion

Provider incentive schemes are increasingly popular levers for improving value and quality in healthcare worldwide, but evidence is needed on the effectiveness of incentivised activities. Despite the vulnerability of people with SMI to poor health outcomes, relatively few studies have examined the impact of care quality indicators in this population. This study addresses that evidence gap, and advances our

For peer review only - http://bmjopen.bmj.com/site/about/guidelines.xhtml

understanding of how primary care can influence utilisation of hospital care in this patient population. We find that care plans are associated with reduced risk of A&E presentation and hospital admissions for conditions amenable to primary care, supporting the hypothesis that this type of care incentivized under the QOF is achieving at least some of its policy objectives and is worth maintaining.

For peer review only

For peer review only - http://bmjopen.bmj.com/site/about/guidelines.xhtml

Author contributions

JR, PK, NG, CK, TD, AM, NR, HG, MG, TK, NS, SG, CD, LA, RW and RJ contributed to the design of the research questions, analytical approach, and interpretation of the findings. JR produced a first draft of the paper and contributed to and PK, NG, CK, TD, AM, NR, HG, MG, TK, NS, SG, CD, LA, RW and RJ approved the final manuscript.

Data sharing

Due to the sensitive and confidential nature of the data used for this analysis, and the permissions required to access it, the dataset is not publicly available.

Ethical approval

The study protocol was approved by the Independent Scientific Advisory Committee of the Clinical Practice Research Datalink (protocol number 15_213R2A2).

Acknowledgements

This research is funded by the National Institute for Health Services & Delivery Research program (project number 135440). The views and opinions expressed are those of the authors and do not necessarily reflect those of the HS&DR program, NIHR, NHS or the Department of Health. We are grateful to the researchers who extracted and provided the CPRD data. We would like to thank all members of our Scientific Steering Committee (SSC) for their invaluable support and feedback on this study. We are grateful to discussants and participants at the following conferences and seminars for their valuable feedback: Thirteenth Workshop on Costs and Assessment in Psychiatry: Mental health policy and economics, Venice, Italy; International Health

For peer review only

Policy Conference, LSE, London; Supportive care, Early Diagnosis and Advanced disease (SEDA) Research Group, Hull York Medical School (HYMS), Hull; University of Toronto, Centre for Addiction and Mental Health (CAMH) and the Canadian Centre for Health Economics (CCHE), Toronto, Canada.

Endnotes

1. We use a twelve-month window rather than the calendar or financial year.
2. We consider any patients with unrelated hospitalisation (e.g. a planned admission for surgery) as having zero hazard of A&E presentation and SMI or ACSC admission during the time they are hospitalised.

Figure 1 legend

Figure 1. Association of care quality indicators with each outcome.

CP: Care plan

AR: Annual review of physical health

For peer review only

REFERENCES

1. Murray CJ, Richards MA, Newton JN, et al. UK health performance: findings of the Global Burden of Disease Study 2010. The Lancet 2013;**381**(9871):997-1020.
2. Vigo D, Thornicroft G, Atun R. Estimating the true global burden of mental illness. The Lancet Psychiatry 2016;**3**(2):171-78.
3. Osborn DP, Levy G, Nazareth I, et al. Relative risk of cardiovascular and cancer mortality in people with severe mental illness from the United Kingdom's General Practice Research Database. Archives of General Psychiatry 2007;**64**(2):242-49.
4. Miller B, Paschall C, Svendsen D. Mortality and medical comorbidity among patients with SMI. Psychiatric Services 2006;**57**(10):1482-87.
5. Wahlbeck K, Westman J, Nordentoft M, et al. Outcomes of Nordic mental health systems: life expectancy of patients with mental disorders. The British Journal of Psychiatry 2011;**199**(6):453-58.
6. Mental Health Statistics team HaSCIC. HES-MHMDS Data Linkage Report, Additional analysis - 2011-12, England (Experimental statistics. Leeds: Health and Social Care Information Centre, 2013.
7. Hackman AL, Goldberg RW, Brown CH, et al. Use of emergency department services for somatic reasons by people with serious mental illness. Psychiatric services 2006;**57**(4):563-66.
8. Merrick EL, Perloff J, Tompkins CP. Emergency department utilization patterns for Medicare beneficiaries with serious mental disorders. Psychiatric services 2010;**61**(6):628-31.
9. Schoenbaum M, Sutherland JM, Chappel A, et al. Twelve-Month Health Care Use and Mortality in Commercially Insured Young People With Incident Psychosis in the United States. Schizophrenia Bulletin 2017;**43**(6):1262-72.
10. Lally J, Wong YL, Shetty H, et al. Acute hospital service utilization by inpatients in psychiatric hospitals. Gen Hosp Psychiat 2015;**37**(6):577-80.
11. Reilly S, Planner C, Hann M, et al. The role of primary care in service provision for people with severe mental illness in the United Kingdom. Plos One 2012;**7**(5):e36468.
12. World Health Organization. Quality improvement for mental health: mental health policy and service guidance package. Geneva: WHO, 2003.
13. World Health Organization. QualityRights tool kit to assess and improve quality and human rights in mental health and social care facilities. Geneva: WHO, 2012.
14. World Health Organisation. Paying for performance in health care: Implications for health system performance and accountability. In: Cashin C, Chi Y-L, Smith P, et al., eds. European Observatory on Health Systems and Policies. Maidenhead, UK: Open University Press, 2014.
15. Eijkenaar F, Emmert M, Scheppach M, et al. Effects of pay for performance in health care: a systematic review of systematic reviews. Health Policy 2013;**110**(2):115-30.
16. Flodgren G, Eccles MP, Shepperd S, et al. An overview of reviews evaluating the effectiveness of financial incentives in changing healthcare professional behaviours and patient outcomes. The Cochrane Library 2011.
17. Doran T, Maurer KA, Ryan AM. Impact of Provider Incentives on Quality and Value of Health Care. Annual Review of Public Health 2017;**38**:449-65.
18. Roland M. Linking physicians' pay to the quality of care—a major experiment in the United Kingdom. New England Journal of Medicine 2004;**351**:1448-54.
19. National collaborating Centre for Mental Health. Psychosis and Schizophrenia in Adults: Treatment and Management. NICE Clinical Guideline 178, 2014.

For peer review only - http://bmjopen.bmj.com/site/about/guidelines.xhtml

20. National Collaborating Centre for Mental Health. Bipolar disorder: The assessment and management of bipolar disorder in adults, children and young people in primary and secondary care. Leicester UK: The British Psychological Society 2014.
21. Gutacker N, Mason AR, Kendrick T, et al. Does the quality and outcomes framework reduce psychiatric admissions in people with serious mental illness? A regression analysis. Bmj Open 2015;**5**(4):e007342.
22. Purdy S, Griffin T, Salisbury C, et al. Ambulatory care sensitive conditions: terminology and disease coding need to be more specific to aid policy makers and clinicians. Public Health 2009;**123**(2):169-73.
23. Wykes TL, Lee AA, McKibbin CL, et al. Self-Efficacy and Hemoglobin A1C Among Adults With Serious Mental Illness and Type 2 Diabetes: The Roles of Cognitive Functioning and Psychiatric Symptom Severity. Psychosomatic Medicine 2016;**78**(3):263-70.
24. Chen SR, Chien YP, Kang CM, et al. Comparing self-efficacy and self-care behaviours between outpatients with comorbid schizophrenia and type 2 diabetes and outpatients with only type 2 diabetes. Journal of Psychiatric and Mental Health Nursing 2014;**21**(5):414-22.
25. Herrett E, Gallagher AM, Bhaskaran K, et al. Data resource profile: clinical practice research datalink (CPRD). Int J Epidemiol 2015;**44**(3):827-36.
26. Booth N. What are the Read codes? Health Libraries Review 1994;**11**(3):177-82.
27. World Health Organization, editor. *The Tenth Revision of the International Classification of Diseases and Related Health Problems (ICD-10)*. Geneva: World Health Organisation, 1992.
28. Bardsley M, Blunt I, Davies S, et al. Is secondary preventive care improving? Observational study of 10-year trends in emergency admissions for conditions amenable to ambulatory care. BMJ Open 2013;**3**(1):e002007.
29. Chang KC-M, Soljak M, Lee JT, et al. Coverage of a national cardiovascular risk assessment and management programme (NHS Health Check): Retrospective database study. Preventive Medicine 2015;**78**:1-8.
30. McLennan D, Barnes H, Noble M, et al. The English indices of deprivation 2010. London: Department for Communities and Local Government 2011.
31. Khan NF, Perera R, Harper S, et al. Adaptation and validation of the Charlson Index for Read/OXMIS coded databases. Bmc Fam Pract 2010;**11**(1):1.
32. Cox DR. Regression Models and Life-Tables. Journal of the Royal Statistical Society Series B (Methodological) 1972;**34**(2):187-220.
33. Yen C-F, Yeh M-L, Chen C-S, et al. Predictive value of insight for suicide, violence, hospitalization, and social adjustment for outpatients with schizophrenia: a prospective study. Comprehensive psychiatry 2002;**43**(6):443-47.
34. Yen C-F, Chen C-S, Yen J-Y, et al. The predictive effect of insight on adverse clinical outcomes in bipolar I disorder: A two-year prospective study. Journal of Affective Disorders 2008;**108**(1):121-27.
35. Baker R, Bankart MJ, Rashid A, et al. Characteristics of general practices associated with emergency-department attendance rates: a cross-sectional study. BMJ Quality & Safety 2011;**20**(11):953-58.
36. Wong CY, Hall J. Does patients’ experience of general practice affect the use of emergency departments? Evidence from Australia. Health Policy 2018;**122**(2):126-33.
37. Xin H, Kilgore ML, Sen B, et al. Can Nonurgent Emergency Department Care Costs be Reduced? Empirical Evidence from a U.S. Nationally Representative Sample. The Journal of Emergency Medicine 2015;**49**(3):347-54.

For peer review only

For peer review only - http://bmjopen.bmj.com/site/about/guidelines.xhtml

38. Harrison MJ, Dusheiko M, Sutton M, et al. Effect of a national primary care pay for performance scheme on emergency hospital admissions for ambulatory care sensitive conditions: controlled longitudinal study. Bmj 2014;**349**:g6423.
39. Wilson CL, Rhodes KM, Payne RA. Financial incentives improve recognition but not treatment of cardiovascular risk factors in severe mental illness. PLOS ONE 2017;**12**(6):e0179392.
40. Berchet C. Emergency Care Services. 2015.
41. Broadbent M, Jarman H, Berk M. Emergency department mental health triage scales improve outcomes. Journal of Evaluation in Clinical Practice 2004;**10**(1):57-62.
42. Eric P. Slade PD, Lisa B. Dixon MD, M.P.H. ,, Sarah Semmel BA. Trends in the Duration of Emergency Department Visits, 2001–2006. Psychiatric Services 2010;**61**(9):878-84.

For peer review only

For peer review only - http://bmjopen.bmj.com/site/about/guidelines.xhtml

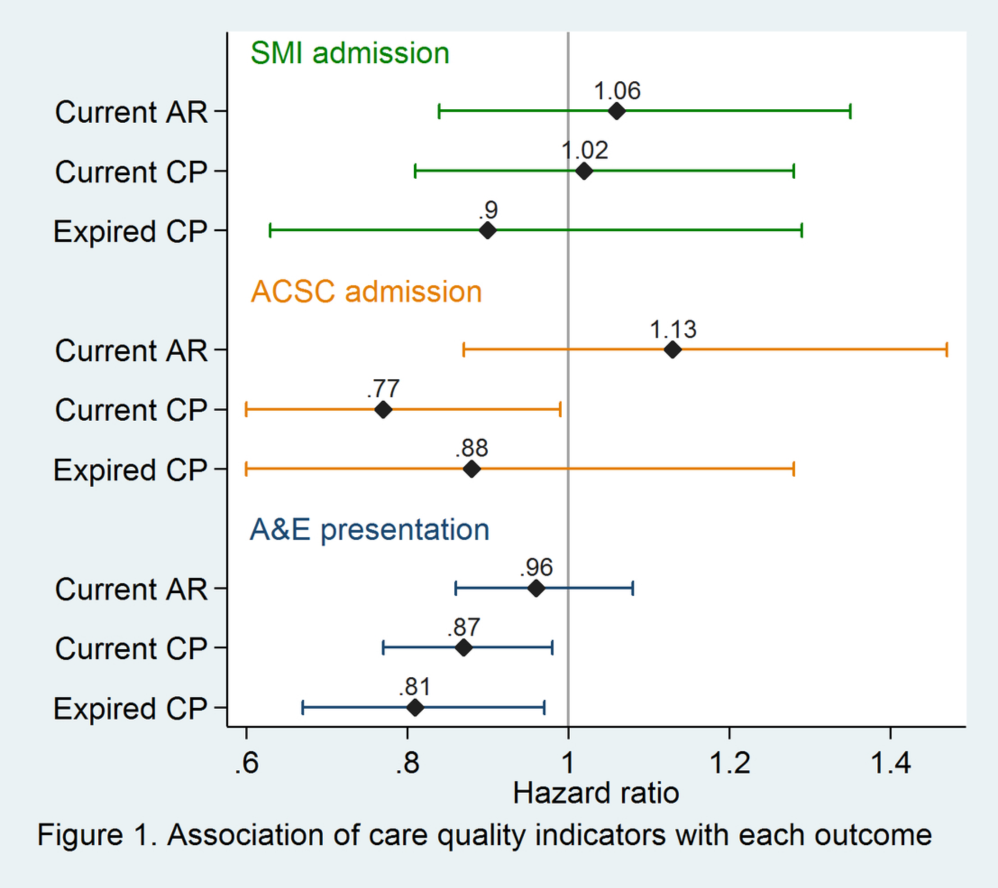

Figure 1. Association of care quality indicators with each outcome.
CP: Care plan
AR: Annual review of physical health

93x83mm (300 x 300 DPI)

Supplementary Table 1: Conditions classified as ambulatory care sensitive admissions^

Condition	ICD-10 codes used to identify the condition#
Angina	I10 I24.0 I24.8 I24.9
Asthma	J45 J46
Chronic obstructive pulmonary disease	J41 J42 J43 J44 J47<br>First diagnosis: J20 + Second diagnosis one of: J41 J42 J43 J44 J47
Congestive heart failure	I50 I11.0 J81
Diabetes (in any diagnosis field)	E10.0 E10.1 E10.2 E10.3 E10.4 E10.5 E10.6 E10.7 E10.8 E11.0 E10.1 E11.2 E10.3 E11.4 E10.5 E11.6 E10.7 E11.8 E12.0 E10.1 E12.2 E10.3 E12.4 E10.5 E12.6 E10.7 E12.8 E13.0 E10.1 E13.2 E10.3 E13.4 E10.5 E13.6 E10.7 E13.8 E14.0 E10.1 E14.2 E10.3 E14.4 E10.5 E14.6 E10.7 E14.8
Epilepsy	G40 G41 O15 R56
Hypertension	I10 I11.9
Anaemia	D50.0 D50.8 D50.9
Cellulitis	L03 L04 L08 L88 L98.0 L98.3
Dehydration	E86
Dental	A69.0 K02 K03 K04 K05 K06 K08 K09.8 K09.9 K12 K13
Ear, nose and throat infections	H66 H67 J02 J03 J06 J31.2
Gangrene (in any diagnosis field)	R02
Gastroenteritis	K52.2 K52.8 K52.9

For peer review only

Nutritional deficiencies	E40 E41 E42 E43 E55 E64.3
Perforated or bleeding ulcer	K25.0 K25.1 K25.2 K25.4 K25.5 K25.6<br>K26.0 K26.1 K26.2 K26.4 K26.5 K26.6<br>K27.0 K27.1 K27.2 K27.4 K27.5 K27.6<br>K28.0 K28.1 K28.2 K28.4 K28.5 K28.6
Urinary tract infection or pyelonephritis	N10 N11 N12 N13.6 N39.0
Influenza (in any diagnosis field, exclude secondary diagnosis of D57)	J10 J11
Pneumonia (in any diagnosis field, exclude secondary diagnosis of D57)	J13 J14 J15.3 J15.4 J15.7 J15.9 J16.8 J18.1 J18.8
Tuberculosis	A15 A16 A19
Other vaccine-preventable diseases (in any diagnosis field)	A35 A36 A37 A80 B05 B06 B16.1 B16.9 B18.0 B18.1<br>B26 G00.0 M01.4

^Based on Bardsley et al. (2013)

#Based on the first diagnosis field in HES data unless otherwise specified.

For peer review only - http://bmjopen.bmj.com/site/about/guidelines.xhtml

For peer review only

Supplementary Table 2: Read codes used to identify SMI diagnostic categories

Category	Read codes used to identify diagnostic category
Schizophrenia and other psychoses	E100.00 E100.11 E100000 E100100 E100200 E100300 E100400 E100500 E100z00 E101.00 E101000 E101400 E101500 E101z00 E102.00 E102000 E102100 E102500 E102z00 E103.00 E103000 E103200 E103300 E103400 E103500 E103z00 E104.00 E105.00 E105000 E105200 E105500 E105z00 E106.00 E107.00 E107.11 E107000 E107100 E107200 E107300 E107400 E107500 E107z00 E10y.00 E10y.11 E10y000 E10y100 E10yz00 E10z.00 E120.00 E121.00 E122.00 E123.00 E123.11 E12y.00 E12y000 E12yz00 E12z.00 E13..00 E13..11 E131.00 E132.00 E133.00 E133.11 E134.00 E13y.00 E13y100 E13yz00 E13z.00 E13z.11 E1z..00 E212200 Eu20.00 Eu20000 Eu20011 Eu20100 Eu20111 Eu20200 Eu20211 Eu20212 Eu20213 Eu20214 Eu20300 Eu20311 Eu20400 Eu20500 Eu20511 Eu20600 Eu20y00 Eu20y12 Eu20y13 Eu20z00 Eu21.00 Eu21.11 Eu21.12 Eu21.13 Eu21.14 Eu21.15 Eu21.16 Eu21.17 Eu21.18 Eu22.00 Eu22000 Eu22011 Eu22012 Eu22013 Eu22014 Eu22015 Eu22100 Eu22111 Eu22200 Eu22300 Eu22y00 Eu22y11 Eu22y12 Eu22y13 Eu22z00 Eu23.00 Eu23000 Eu23011 Eu23012 Eu23100 Eu23112 Eu23200 Eu23211 Eu23212 Eu23214 Eu23300 Eu23312 Eu23y00 Eu23z00 Eu23z11 Eu23z12 Eu24.00 Eu24.12 Eu24.13 Eu25.00 Eu25000 Eu25011 Eu25012 Eu25100 Eu25111 Eu25112 Eu25200 Eu25212 Eu25y00 Eu25z00 Eu25z11 Eu26.00 Eu2y.00 Eu2y.11 Eu2z.00 Eu2z.11 Eu44.14

For peer review only - http://bmjopen.bmj.com/site/about/guidelines.xhtml

Bipolar disorder and affective psychoses	E11..00 E11..12 E110.00 E110.11 E110000 E110100 E110200 E110300 E110400 E110600 E110z00 E111.00 E111000 E111100 E111200 E111300 E111400 E111500 E111600 E111z00 E112400 E113400 E114.00 E114.11 E114000 E114100 E114200 E114300 E114400 E114500 E114600 E114z00 E115.00 E115.11 E115000 E115100 E115200 E115300 E115400 E115500 E115600 E115z00 E116.00 E116000 E116100 E116200 E116300 E116400 E116500 E116600 E116z00 E117.00 E117000 E117100 E117200 E117300 E117400 E117500 E117600 E117z00 E11y.00 E11y000 E11y100 E11y300 E11yz00 E11z.00 E11z000 E11zz00 E130.00 E130.11 E13y000 Eu30.00 Eu30.11 Eu30000 Eu30100 Eu30200 Eu30211 Eu30212 Eu30y00 Eu30z00 Eu30z11 Eu31.00 Eu31.11 Eu31.12 Eu31.13 Eu31000 Eu31100 Eu31200 Eu31300 Eu31400 Eu31500 Eu31600 Eu31700 Eu31800 Eu31900 Eu31911 Eu31y00 Eu31y11 Eu31y12 Eu31z00 Eu32300 Eu32311 Eu32312 Eu32313 Eu32314 Eu32800 Eu33213 Eu33300 Eu33311 Eu33312 Eu33313 Eu33314 Eu33315 Eu33316 Eu3z.11

For peer review only

Rates of care indicators over the study period

Supplementary Figure 1 shows the rate of care indicators recorded by GP practices per patient-year for each financial year in our sample. This rate is the number of times an indicator is recorded in that year across all patients, divided by the number of patient-years of observation in that financial year (which takes into account partial years for patients who were not observed for the full financial year). For example, in 2007/08 the rate of annual reviews (composite measure) recorded was 1.06 per patient-year.

The CP indicator shows a decline over the study period until 2012/13, when there was an increase in the threshold of achievement required to attain the maximum payment. The original AR indicator shows a marked drop-off after it ceased to be incentivized in 2010/11, while the aggregate indicator composed of the 'health risk' checks shows less marked change but a small increase after the specific components began to be incentivized in 2011/12. The composite of these two AR indicators shows little change in response to the QOF rule changes.

Supplementary Figure 1: Rate of care indicators per patient-year

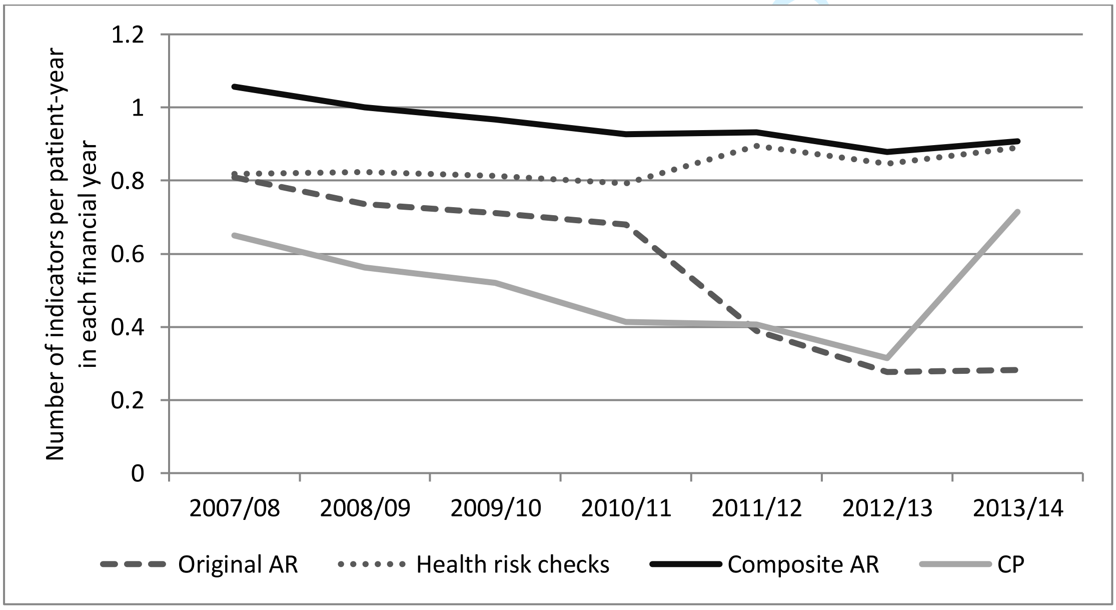

For peer review only - http://bmjopen.bmj.com/site/about/guidelines.xhtml

Survival analysis

We estimate the following hazard function for each outcome, which is the product of the baseline hazard, $\lambda_0(t)$, and an exponentiated linear function of patients' characteristics fixed at baseline (X_i) and care quality indicators for patient *i* at practice *g* at time *t*:

$$h_{ig}(t) = \lambda_{0_g}(t)\exp\{\beta_1 X_{ig1} + \cdots + \beta_k X_{igk} + \alpha_1 CP_{ig}^{cur.}(t) + \alpha_2 CP_{ig}^{exp.}(t) + \alpha_3 AR_{ig}(t)\} \quad (1)$$

The care quality indicators are represented by three time-varying variables:

$$CP_i^{cur.}(t) = \begin{cases} = 1 \text{ if a care plan has been documented in the last 12 months} \\ = 0 \text{ if a care plan has not been documented in the last 12 months} \end{cases}$$

$$CP_i^{exp.}(t) = \begin{cases} = 1 \text{ if a care plan has been documented before the last 12 months} \\ = 0 \text{ if a care plan has never been documented} \end{cases}$$

$$AR_i(t) = \begin{cases} = 1 \text{ if an annual review has been documented in the last 12 months} \\ = 0 \text{ if an annual review has not been documented in the last 12 months} \end{cases}$$

λ_{0_g}: the baseline hazard is allowed to vary by practice

For peer review only - http://bmjopen.bmj.com/site/about/guidelines.xhtml

Supplementary Table 3: Distribution of person-years observed across patient characteristics in sample for analysis of A&E presentations

Sample for A&E presentations	Person-years overall	Care plans			Annual reviews	
		% of row person-years with current CP	% of row person-years with expired CP	% of row person-years with no CP	% of row person-years with current AR	% of row person-years with no current AR
Sample overall	6,654	40%	20%	40%	56%	44%
Age at diagnosis						
18-35	1,901	41%	19%	40%	49%	51%
36-45	1,496	41%	20%	39%	53%	47%
46-55	1.233	41%	23%	36%	62%	38%
56-65	842	39%	20%	41%	62%	38%
>65	1,181	37%	16%	47%	58%	42%
Index of multiple deprivation						
Quintile 1 (least deprived)	1,246	43%	21%	36%	59%	41%
Quintile 2	1,326	41%	185	41%	58%	42%
Quintile 3	1,188	37%	20%	43%	52%	48%
Quintile 4	1,464	40%	20%	40%	55%	45%
Quintile 5 (most deprived)	1,430	39%	19%	42%	54%	46%
Gender						
Male	3,131	41%	20%	39%	54%	46%
Female	3,522	39%	20%	41%	57%	43%
Ethnicity						
White	4,620	40%	19%	41%	56%	44%
Black & minority ethnicities	2,034	40%	21%	39%	54%	46%
Number of primary care contacts in year preceding diagnosis						
0-4	1,726	38%	22%	40%	49%	51%
5-9	1,894	42%	19%	39%	56%	44%
10-14	1,255	41%	18%	41%	58%	42%

For peer review only - http://bmjopen.bmj.com/site/about/guidelines.xhtml

For peer review only

Sample for A&E presentations	Person-years overall	Care plans: % of row person-years with current CP	% of row person-years with expired CP	% of row person-years with no CP	Annual reviews: % of row person-years with current AR	% of row person-years with no current AR
15-19	694	40%	18%	42%	59%	41%
>=20	1,085	40%	18%	42%	61%	39%
Number of hospital admissions in year preceding diagnosis						
0	4,119	38%	20%	42%	55%	45%
1	1,510	43%	21%	36%	56%	44%
2	611	46%	18%	36%	62%	38%
3	414	40%	19%	41%	57%	43%
Number of Charlson Index comorbidities at time of diagnosis						
0	4,346	40%	20%	40%	54%	46%
1	1,702	41%	20%	39%	58%	42%
2	405	34%	15%	51%	61%	39%
3 or more	201	31%	16%	52%	64%	36%
Comorbid depression at time of diagnosis						
History of depression	4,409	41%	19%	40%	56%	44%
No recorded history of depression	2,245	39%	21%	40%	54%	46%
Smoking status						
Current or ex-smoker	4,976	39%	19%	42%	56%	44%
No recorded history of smoking	1,678	42%	21%	37%	56%	44%
SMI diagnostic group						
Schizophrenia & other psychoses	3,427	40%	20%	40%	54%	46%
Bipolar disorder & affective psychoses	2,887	40%	19%	41%	57%	43%
Both	341	47%	22%	31%	61%	39%
Financial year of diagnosis						

For peer review only - http://bmjopen.bmj.com/site/about/guidelines.xhtml

Sample for A&E presentations	Person-years overall	Care plans			Annual reviews	
		% of row person-years with current CP	% of row person-years with expired CP	% of row person-years with no CP	% of row person-years with current AR	% of row person-years with no current AR
2007/08	1,407	43%	30%	27%	65%	35%
2008/09	1,265	40%	28%	32%	61%	39%
2009/10	1,159	38%	23%	39%	59%	41%
2010/11	931	41%	18%	41%	57%	43%
2011/12	937	38%	9%	53%	49%	51%
2012/13	684	39%	2%	59%	40%	60%
2013/14	272	39%	0	61%	29%	71%

For peer review only

Supplementary Table 4: Distribution of person-years observed across patient characteristics in sample for analysis of SMI admissions

Sample for SMI admissions	Person-years overall	Care plans			Annual reviews	
		% of row person-years with current CP	% of row person-years with expired CP	% of row person-years with no CP	% of row person-years with current AR	% of row person-years with no current AR
Sample overall	12,724	39%	27%	34%	59%	41%
Age at diagnosis						
18-35	3,825	40%	26%	34%	51%	29%
36-45	2,886	39%	29%	32%	57%	43%
46-55	2,080	40%	27%	33%	65%	35%
56-65	1,570	38%	29%	33%	68%	32%
>65	2,362	35%	24%	41%	63%	37%
Index of multiple deprivation						
Quintile 1 (least deprived)	2,255	39%	27%	34%	60%	40%
Quintile 2	2,394	40%	26%	34%	60%	40%
Quintile 3	2,327	35%	29%	36%	56%	44%
Quintile 4	2,874	40%	27%	33%	60%	40%
Quintile 5 (most deprived)	2,875	39%	26%	35%	58%	42%
Gender						
Male	5,976	38%	28%	34%	55%	45%
Female	6,748	39%	27%	34%	62%	38%
Ethnicity						
White	9,154	38%	27%	35%	59%	41%
Black & minority ethnicities	3,570	38%	28%	34%	58%	42%
Number of primary care contacts in year preceding diagnosis						
0-4	2,913	37%	29%	34%	50%	50%
5-9	3,587	39%	27%	34%	57%	43%
10-14	2,373	40%	27%	33%	60%	40%

For peer review only - http://bmjopen.bmj.com/site/about/guidelines.xhtml

Sample for SMI admissions	Person-years overall	Care plans: % of row person-years with current CP	Care plans: % of row person-years with expired CP	Care plans: % of row person-years with no CP	Annual reviews: % of row person-years with current AR	Annual reviews: % of row person-years with no current AR
15-19	1,422	39%	25%	36%	63%	37%
>=20	2,429	38%	26%	36%	68%	32%
Number of hospital admissions in year preceding diagnosis						
0	7,723	37%	28%	35%	57%	43%
1	2,899	41%	27%	32%	60%	40%
2	1,192	42%	25%	33%	64%	36%
3	910	38%	26%	36%	62%	38%
Number of Charlson Index comorbidities at time of diagnosis						
0	7,970	39%	28%	33%	56%	44%
1	3,472	40%	27%	33%	62%	38%
2	849	33%	22%	45%	66%	34%
3 or more	432	31%	23%	46%	68%	32%
Comorbid depression at time of diagnosis						
History of depression	8,641	40%	27%	33%	60%	40%
No recorded history of depression	4,083	37%	27%	36%	56%	44%
Smoking status						
Current or ex-smoker	9,823	39%	27%	34%	59%	41%
No recorded history of smoking	2,901	39%	27%	34%	58%	42%
SMI diagnostic group						
Schizophrenia & other psychoses	6,394	39%	28%	33%	61%	39%
Bipolar disorder & affective psychoses	5,811	39%	28%	33%	61%	39%
Both	582	44%	30%	26%	63%	37%
Financial year of diagnosis						

For peer review only

Sample for SMI admissions	Person-years overall	Care plans			Annual reviews	
		% of row person-years with current CP	% of row person-years with expired CP	% of row person-years with no CP	% of row person-years with current AR	% of row person-years with no current AR
2006/07	2,752	38%	39%	23%	65%	35%
2007/08	2,077	40%	34%	26%	64%	36%
2008/09	2,006	38%	32%	30%	62%	38%
2009/10	1,818	36%	29%	35%	59%	41%
2010/11	1,454	42%	21%	37%	60%	40%
2011/12	1,402	37%	12%	51%	50%	50%
2012/13	901	40%	3%	57%	42%	58%
2013/14	314	40%	0	60%	28%	72%

For peer review only

Supplementary Table 5: Distribution of person-years observed across patient characteristics in sample for analysis of ACSC admissions

Sample for ACSC admissions	Person-years overall	Care plans			Annual reviews	
		% of row person-years with current CP	% of row person-years with expired CP	% of row person-years with no CP	% of row person-years with current AR	% of row person-years with no current AR
Sample overall	13,203	40%	27%	33%	59%	41%
Age at diagnosis						
18-35	4,111	41%	27%	32%	52%	48%
36-45	2,990	40%	29%	31%	57%	43%
46-55	2,215	41%	29%	31%	65%	35%
56-65	1,618	41%	28%	31%	68%	32%
>65	2,269	37%	23%	40%	63%	37%
Index of multiple deprivation						
Quintile 1 (least deprived)	2,364	41%	27%	32%	61%	39%
Quintile 2	2,500	41%	27%	32%	61%	39%
Quintile 3	2,408	37%	29%	34%	56%	44%
Quintile 4	2,979	42%	26%	32%	60%	40%
Quintile 5 (most deprived)	2,952	40%	26%	34%	58%	42%
Gender						
Male	6,305	40%	27%	33%	56%	44%
Female	6,898	40%	27%	33%	62%	38%
Ethnicity						
White	9,527	40%	27%	33%	60%	40%
Black & minority ethnicities	3,676	39%	27%	34%	57%	43%
Number of primary care contacts in year preceding diagnosis						
0-4	3,209	38%	29%	33%	51%	49%
5-9	3,746	41%	27%	32%	58%	42%
10-14	2,415	41%	27%	32%	61%	39%

For peer review only

15-19	1,447	40%	25%	35%	63%	27%
>=20	2,386	39%	27%	34%	67%	33%
Number of hospital admissions in year preceding diagnosis						
0	8,010	38%	28%	34%	58%	42%
1	3,071	43%	27%	30%	60%	40%
2	1,221	44%	25%	31%	63%	37%
3	901	41%	26%	33%	63%	37%
Number of Charlson Index comorbidities at time of diagnosis						
0	8,502	40%	28%	32%	57%	43%
1	3,534	41%	27%	32%	62%	38%
2	778	35%	22%	43%	66%	34%
3 or more	389	30%	23%	47%	68%	32%
Comorbid depression at time of diagnosis						
History of depression	8,860	41%	27%	32%	60%	40%
No recorded history of depression	4,343	38%	28%	34%	57%	43%
Smoking status						
Current or ex-smoker	10,118	40%	27%	33%	60%	40%
No recorded history of smoking	3,085	40%	28%	32%	58%	42%
SMI diagnostic group						
Schizophrenia & other psychoses	6,687	40%	26%	34%	57%	43%
Bipolar disorder & affective psychoses	5,820	40%	28%	32%	61%	39%
Both	696	44%	32%	24%	66%	34%
Financial year of diagnosis						
2006/07	2,884	39%	39%	21%	65%	35%
2007/08	2,151	42%	34%	24%	65%	35%
2008/09	2,033	40%	31%	29%	63%	37%
2009/10	1,936	38%	30%	32%	60%	40%
2010/11	1,511	42%	22%	36%	60%	40%

For peer review only - http://bmjopen.bmj.com/site/about/guidelines.xhtml

2011/12	1,438	38%	12%	50%	50%	50%
2012/13	932	41%	2%	56%	43%	57%
2013/14	319	40%	0	60%	29%	71%

For peer review only

Supplementary Table 6: Prevalence of Charlson Index comorbidities at time of SMI diagnosis

Comorbidities at time of SMI diagnosis	Proportion of sample (%)
Cancer	7.31
Cerebrovascular disease	5.08
COPD/ Asthma	19.72
CHD	2.12
Dementia	3.06
Diabetes	7.37
Hemiplegia	0.60
Liver disease	0.78
Myocardial infarction	2.68
Peptic ulcer	2.54
Peripheral vascular disease	1.96
Renal disease	7.60
Rheumatological disease	2.56

For peer review only

Supplementary Table 7: Main model - full results with A&E presentation as the outcome

		Main model	
		HR	(95% CI)
Current annual review		0.962	(0.856-1.080)
Current care plan		0.866*	(0.766-0.979)
Expired care plan		0.808*	(0.672-0.972)
Age at diagnosis			
	18-35 (base)		
	36-45	0.806*	(0.657-0.988)
	46-55	0.619***	(0.507-0.755)
	56-65	0.582***	(0.460-0.738)
	>=66	1.098	(0.917-1.314)
Male		1.016	(0.861-1.199)
Age at diagnosis*Male			
	18-35*Male (base)		
	36-45*Male	0.919	(0.694-1.216)
	46-55*Male	1.215	(0.911-1.614)
	56-65*Male	1.432*	(1.037-1.978)
	≥66*Male	0.939	(0.727-1.203)
SMI diagnosis group			
Bipolar disorder or affective psychosis (base)			
Schizophrenia or other psychosis		1.103	(0.997-1.220)
Both		1.119	(0.888-1.411)
IMD			
	1st quintile (base)		

For peer review only

		Main model	
		HR	(95% CI)
	2nd quintile	0.955	(0.804-1.133)
	3rd quintile	1.100	(0.918-1.319)
	4th quintile	1.178	(0.978-1.419)
	5th quintile	1.196	(0.985-1.453)
Ethnicity white		1.277***	(1.149-1.421)
Number of admissions within 12 months prior to diagnosis			
	0 (base)		
	1	1.174**	(1.050-1.313)
	2	1.190*	(1.028-1.377)
	3 or more	1.500***	(1.268-1.775)
Number of primary care contacts within 12 months prior to diagnosis			
	<5 (base)		
	5-9	1.219**	(1.056-1.407)
	10-14	1.334***	(1.165-1.528)
	15-19	1.714***	(1.459-2.013)
	>=20	1.861***	(1.592-2.176)
Current or ex-smoker		1.101	(0.983-1.233)
Number of Charlson Index comorbidities		1.111***	(1.052-1.174)
Diagnosis of depression		1.005	(0.904-1.118)
Year of diagnosis			
	2007 (base)		

For peer review only

	Main model	
	HR	(95% CI)
2008	1.263*	(1.040-1.532)
2009	1.383***	(1.149-1.665)
2010	1.644***	(1.356-1.994)
2011	1.551***	(1.244-1.933)
2012	1.385**	(1.105-1.737)
2013	1.266	(0.990-1.614)
2014	0.970	(0.557-1.691)
Practice list size	1.000	(1.000-1.000)
Number of patients	4,446	

*p<0.05, **p<0.01, ***p<0.001

For peer review only

For peer review only - http://bmjopen.bmj.com/site/about/guidelines.xhtml

Supplementary Table 8: Sensitivity tests with A&E presentation as the outcome

Model specifications	Current AR		Current CP		Expired CP	
	HR	(95% CI)	HR	(95% CI)	HR	(95% CI)
Main analysis	0.96	(0.86-1.08)	0.87*	(0.77-0.98)	0.81*	(0.67-0.97)
AR using QOF codes only (limited to 2010/11)	0.92	(0.74-1.15)	0.88	(0.68-1.15)	0.64*	(0.42-0.99)
CP and AR current for 15 months	0.93	(0.83-1.05)	0.87*	(0.77-0.98)	0.85	(0.69-1.04)
Number of Charlson index comorbidities varies over time	0.96	(0.85-1.07)	0.87*	(0.77-0.98)	0.81*	(0.67-0.98)
Observed practice characteristics and cluster-adjusted SEs	0.95	(0.86-1.05)	0.89*	(0.80-0.99)	0.82*	(0.69-0.96)
Fixed practice effects and cluster-adjusted SEs	0.96	(0.86-1.06)	0.87*	(0.77-0.97)	0.83*	(0.70-0.98)

*p<0.05, **p<0.01, ***p<0.001

CP: Care plan (base: never had a care plan)

AR: Annual review (base: no annual review in last 12 months)

Hazard ratios represent the relative hazard of a patient with a care indicator compared with an otherwise identical patient with the base level care indicator.

For peer review only

Supplementary Table 9: Sensitivity tests with SMI admission as the outcome

Model specifications	Current AR		Current CP		Expired CP	
	HR	(95% CI)	HR	(95% CI)	HR	(95% CI)
Main analysis	1.06	(0.84-1.35)	1.02	(0.81-1.28)	0.90	(0.63-1.29)
AR using original codes only (limited to 2010/11)	1.03	(0.72-1.47)	1.01	(0.69-1.47)	0.84	(0.46-1.53)
CP and AR current for 15 months	1.06	(0.82-1.36)	1.00	(0.79-1.27)	1.00	(0.68-1.48)
Number of Charlson index comorbidities varies over time	1.08	(0.86-1.37)	1.00	(0.80-1.26)	0.89	(0.62-1.27)
Observed practice characteristics and cluster-adjusted SEs	1.08	(0.87-1.34)	1.05	(0.84-1.31)	0.98	(0.72-1.32)
Fixed practice effects and cluster-adjusted SEs	1.11	(0.88-1.40)	1.06	(0.85-1.33)	1.04	(0.75-1.43)

*p<0.05, **p<0.01, ***p<0.001

CP: Care plan (base: never had a care plan)

AR: Annual review (base: no annual review in last 12 months)

Hazard ratios represent the relative hazard of a patient with a care indicator compared with an otherwise identical patient with the base level care indicator.

For peer review only - http://bmjopen.bmj.com/site/about/guidelines.xhtml

Supplementary Table 10: Sensitivity tests with ACSC admission as the outcome

Model specifications	Current AR		Current CP		Expired CP	
	HR	(95% CI)	HR	(95% CI)	HR	(95% CI)
Main analysis	1.13	(0.87-1.47)	0.77*	(0.60-0.99)	0.88	(0.60-1.28)
AR using original codes only (limited to 2010/11)	0.85	(0.57-1.28)	1.07	(0.67-1.70)	0.84	(0.44-1.58)
CP and AR current for 15 months	1.08	(0.83-1.41)	0.79	(0.61-1.02)	0.90	(0.62-1.30)
Number of Charlson index comorbidities varies over time	1.08	(0.83-1.42)	0.79	(0.61-1.02)	0.92	(0.62-1.36)
Observed practice characteristics and cluster-adjusted SEs	1.05	(0.84-1.32)	0.79*	(0.63-0.99)	0.88	(0.63-1.22)
Fixed practice effects and cluster-adjusted SEs	1.03	(0.81-1.30)	0.82	(0.64-1.05)	0.95	(0.66-1.35)

*p<0.05, **p<0.01, ***p<0.001

CP: Care plan (base: never had a care plan)

AR: Annual review (base: no annual review in last 12 months)

Hazard ratios represent the relative hazard of a patient with a care indicator compared with an otherwise identical patient with the base level care indicator.

For peer review only - http://bmjopen.bmj.com/site/about/guidelines.xhtml

BMJ Open

Do care plans and annual reviews of physical health influence unplanned hospital utilisation for people with serious mental illness? Analysis of linked longitudinal primary and secondary healthcare records in England.

Journal:	*BMJ Open*
Manuscript ID	bmjopen-2018-023135.R2
Article Type:	Research
Date Submitted by the Author:	09-Oct-2018
Complete List of Authors:	Ride, Jemimah; University of York Centre for Health Economics,<br>Kasteridis, Panagiotis; University of York Centre for Health Economics<br>Gutacker, Nils; University of York, Centre for Health Economics<br>Kronenberg, Christoph; University Duisburg-Essen, CINCH ; Leibniz Science Campus Ruhr<br>Doran, Tim; University of York, Health Sciences<br>Mason, Anne; University of York, Centre for Health Economics<br>Rice, Nigel; University of York Centre for Health Economics<br>Gravelle, Hugh; University of York Centre for Health Economics<br>Goddard, Maria; University of York, Centre for Health Economics<br>Kendrick, Tony; University of Southampton, Primary Care and Population Sciences<br>Siddiqi, Najma; University of York, Psychiatry, Hull York Medical School, York and Bradford District Care NHS Foundation Trust, Bradford, UK<br>Gilbody, Simon; The University of York, Department of Health Sciences<br>Dare, Ceri; McPin Foundation,<br>Aylott, Lauren<br>Williams, Rachael; Clinical Practice Research Datalink,<br>Jacobs, Rowena; University of York, Centre for Health Economics
**Primary Subject Heading**:	Health economics
Secondary Subject Heading:	Health policy, Mental health, General practice / Family practice, Health services research
Keywords:	Schizophrenia & psychotic disorders < PSYCHIATRY, PRIMARY CARE, Quality in health care < HEALTH SERVICES ADMINISTRATION & MANAGEMENT, Data linkage, Hospital utilisation

SCHOLARONE™
Manuscripts

Do care plans and annual reviews of physical health influence unplanned hospital utilisation for people with serious mental illness? Analysis of linked longitudinal primary and secondary healthcare records in England.

Authors:

Jemimah Ride (corresponding author): Centre for Health Economics, Alcuin 'A' Block, University of York, Heslington, YO10 5DD, United Kingdom. jemimah.ride@york.ac.uk (+44) 01904 321 459

Panagiotis Kasteridis[a] (panos.kasteridis@york.ac.uk); Nils Gutacker [a] (nils.gutacker@york.ac.uk); Christoph Kronenberg [b] (christoph.kronenberg@uni-due.de); Tim Doran[c] (tim.doran@york.ac.uk); Anne Mason [a] (anne.mason@york.ac.uk); Nigel Rice [a] (nigel.rice@york.ac.uk); Hugh Gravelle [a] (hugh.gravelle@york.ac.uk); Maria Goddard [a] (maria.goddard@york.ac.uk); Tony Kendrick [d] (A.R.Kendrick@soton.ac.uk); Najma Siddiqi [c] (najma.siddiqi@york.ac.uk); Simon Gilbody [c] (simon.gilbody@york.ac.uk); Ceri R Dare[e] (ceri.owen@gmail.com); Lauren Aylott[e] (lmeaylott@gmail.com); Rachael Williams[f] (Rachael.Williams@mhra.gov.uk); Rowena Jacobs[a] (rowena.jacobs@york.ac.uk)

a: Centre for Health Economics, University of York

b: CINCH, University Duisburg-Essen, Essen, Germany; Leibniz Science Campus Ruhr, Essen, Germany; RWI – Leibniz-Institute for Economic Research, Essen, Germany

c: Department of Health Sciences, University of York

d: Primary Care and Population Sciences, University of Southampton

e: Service user

f: Clinical Practice Research Datalink

Word count: 3,922

For peer review only - http://bmjopen.bmj.com/site/about/guidelines.xhtml

ABSTRACT

Objective: To investigate whether two primary care activities that are framed as indicators of primary care quality (comprehensive care plans and annual reviews of physical health) influence unplanned utilisation of hospital services for people with serious mental illness (SMI).

Design, setting, participants: Retrospective observational cohort study using linked primary care and hospital records (Hospital Episode Statistics) for 5,158 patients diagnosed with SMI between April 2006 and March 2014, who attended 213 primary care practices in England that contribute to the Clinical Practice Research Datalink GOLD database.

Outcomes & analysis: Cox survival models were used to estimate the associations between two primary care quality indicators (care plans and annual reviews of physical health) and the hazards of three types of unplanned hospital utilisation: presentation to Accident & Emergency departments (A&E), admission for SMI, and admission for ambulatory care sensitive conditions (ACSC).

Results: Risk of A&E presentation was 13% lower (hazard ratio (HR) 0.87, 95% CI 0.77-0.98) and risk of admission to hospital for ACSC was 23% lower (HR 0.77, 95%CI 0.60-0.99) for patients with a care plan documented in the previous year compared to those without a care plan. Risk of A&E presentation was 19% lower for those who had a care plan documented earlier but not updated in the previous year (HR: 0.81, 95% CI 0.67-0.97) compared to those without a care plan. Risks of hospital admission for SMI were not associated with care plans, and none of the outcomes were associated with annual reviews.

Conclusions*:* Care plans documented in primary care for people with SMI are associated with reduced risk of A&E attendance and reduced risk of unplanned

For peer review only - http://bmjopen.bmj.com/site/about/guidelines.xhtml

admission to hospital for physical health problems, but not with risk of admission for mental health problems. Annual reviews of physical health are not associated with risk of unplanned hospital utilisation.

For peer review only

For peer review only - http://bmjopen.bmj.com/site/about/guidelines.xhtml

Strengths and limitations of this study

- Innovative use of linked individual-level patient data from primary care, inpatient admissions and A&E, allowing the sequence of care indicators and unplanned hospital utilisation to be identified.
- Contributes to a limited evidence base on the association of patient outcomes with the types of care incentivised under a national primary care physician incentive scheme.
- Due to the observational nature of the data, no information was available on the circumstances leading to documentation of care quality indicators, or their quality or content.
- Partial capacity to account for patient health status and factors driving utilisation of primary and secondary care.

Keywords

Schizophrenia & psychotic disorders; primary care; quality in health care; data linkage; hospital utilisation.

Funding statement

This project was funded by the National Institute for Health Research HS&DR programme (project number 13/54/40). The views expressed are those of the author(s) and not necessarily those of the NHS, the NIHR or the Department of Health.

Competing interests

No competing interests.

For peer review only - http://bmjopen.bmj.com/site/about/guidelines.xhtml

INTRODUCTION

Serious mental illness (SMI) comprises a set of conditions including schizophrenia and bipolar disorder with profound impacts on the wellbeing of patients and high costs to society. It is linked with a high disease burden[1 2], poor health outcomes, high treatment costs, and lower life expectancy, primarily attributed to preventable physical causes[3-6]. Patients with SMI have high rates of A&E attendance and hospital admission, for both physical and mental health problems[7-10].

In the UK, while specialist mental health services are important to many patients' care, general practice provides the majority of care for patients with SMI[11]. High quality primary care therefore has the potential to improve the management, health and wellbeing of patients with SMI, and can help meet the aims of policymakers seeking to improve population wellbeing and contain costs[12 13].

Several countries have adopted incentive programs to improve the quality and value of healthcare[14], despite mixed evidence of their effectiveness[15-17]. England introduced a voluntary scheme for primary care in 2004, the Quality and Outcomes Framework (QOF), offering GPs incentives to meet quality targets for patients with several chronic conditions, including SMI[18]. Two key SMI indicators in the QOF, which apply to all registered patients with SMI, promote proactive management of physical and mental health: the proportion of patients on the practice SMI register who had an in-date comprehensive care plan, and the proportion who had an annual physical health review.

For peer review only - http://bmjopen.bmj.com/site/about/guidelines.xhtml

There is little evidence on whether these incentivised activities improve patients' mental or physical health, modify patterns of healthcare utilisation, or reduce costs. They derive from NICE guideline recommendations for primary care management of SMI, which are based on consensus in the absence of robust evidence[19 20].

A previous study using data aggregated to practice level found that higher rates of SMI admission were associated with higher achievement on the annual physical review indicator, but not the care plan indicator[21]. However, that study could not ascertain which individuals within a practice received care or whether the care preceded or followed admissions.

As the QOF indicators are regularly reviewed and revised, new evidence on their potential impacts is useful for decision-makers. Our study contributes such evidence, examining whether care plans and annual reviews are associated with utilisation of unplanned secondary care for patients with SMI, by analysing linked primary care and hospital records. We hypothesise that care plans and annual reviews may modify risk of unplanned hospital utilisation, which is potentially preventable by high-quality primary care[22]. Patients with SMI are less likely to report health problems than their peers due to self-neglect, impaired motivation, and social withdrawal[23 24]. Proactive care by GPs may help to identify and manage physical and mental health problems without the need for hospital care.

For peer review only - http://bmjopen.bmj.com/site/about/guidelines.xhtml

METHODS

Study design

In this retrospective observational cohort study using linked primary care and hospital data, we investigate the relationship between having a care plan and/or an annual physical health review, and time from diagnosis of SMI to unplanned hospital utilisation.

Data sources and linkage

The main data source is the Clinical Practice Research Datalink (CPRD) GOLD, which holds linked individual-level anonymized primary care records from participating general practices in the UK. These data are representative of the English population with respect to age and gender, but not region. For example, the north-east of England is under-sampled relative to areas in the west and south[25]. The CPRD data service provided information on all patients eligible for linkage and registered with a participating practice in England, and with a diagnosis of SMI documented on or before 31 March 2014 recorded in clinical notes or referral records, using the event date entered by the GP. The sample was limited to patients whose records met an acceptable standard based on recording of registration, clinical events, and demographic details, at practices deemed up to standard according to a CPRD algorithm[25]. Most of the primary care information is recorded using a hierarchical coding system known as 'Read codes'[26], which we use to identify records of SMI diagnosis, care quality indicators, and morbidity profiles. CPRD records were linked to Hospital Episode Statistics (HES), which comprise detailed records for all NHS patients admitted to hospital and presentations to A&E in England. CPRD provided deterministically linked admission and A&E data for all patients included in the study.

For peer review only - http://bmjopen.bmj.com/site/about/guidelines.xhtml
For peer review only

To preserve anonymity, all linkages were carried out under the CPRD routine linkage scheme.

Inclusion and exclusion criteria

Our sample covers eight financial years, from 1 April 2006 to 31 March 2014. The care plan indicator was introduced in 2006/07 and the last year of HES data available to us at the start of the study was 2013/14. The sample comprises patients aged 18 years and over, whose earliest recorded diagnosis of SMI in primary care was after 31 March 2006, and who were registered with the same practice for at least 365 days before that diagnosis. A&E data are only available from 2007/08, so the analysis of A&E presentations is limited to patients with a first diagnosis after 31 March 2007. The restriction to newly diagnosed patients excludes those whose unobserved past care or events could influence their subsequent care.

Outcomes

We analyse three outcomes taken from HES data: time to i) presentations to Accident and Emergency departments (A&E), ii) unplanned hospital admission for SMI, and iii) unplanned admissions for ambulatory care sensitive conditions (ACSCs). Unplanned admissions, based on the HES admission method codes for emergency admissions, were classified by International Classification of Diseases (ICD-10) codes. For SMI admissions this included admissions with primary diagnosis field code categories F20-F31[27]. For ACSC, the codes were those defined by Bardsley, et al.[28] (see Supplementary Table 1 for the list of conditions included). A&E presentations of any cause (mental or physical health) were included.

For peer review only - http://bmjopen.bmj.com/site/about/guidelines.xhtml

Care quality indicators

Primary care quality indicators were taken from CPRD data, and capture care plans and annual reviews of physical health based on Read codes specified under the QOF to identify those two indicators. A good quality care plan documents the patient's current health and social care needs, and how these will be addressed, in agreement with patients and caregivers. It should specify arrangements with secondary care services (where applicable), and recommendations in case of relapse, including the patient's care preferences and goals[19 20]. The QOF incentivises GPs to document care plans annually, so we analyse 'current' care plans, those recorded in the last twelve months. However, we hypothesise that an older care plan may still influence patients' health service utilisation, and therefore also analyse 'expired' care plans, those recorded more than a year ago. Conversely, only 'current' annual reviews (recorded within the last twelve months) are included in the analysis, since expired monitoring of physical health is unlikely to have ongoing benefits. The choice of the twelve-month window to determine expiration status is based on the QOF guidance that a care plan or annual review should be reviewed annually[3].

From 2006/7 to 2010/11 the QOF annual review indicator entailed the patient being given appropriate health promotion and prevention advice. In 2011/12, this broad indicator was split into more specific indicators: a record of alcohol consumption, checks of blood pressure, body mass index (BMI), blood glucose or glycated haemoglobin, and ratio of total to high-density lipoprotein cholesterol, and, where appropriate, cervical screening. In order to explore annual reviews for the full period of our analysis, we formulate an aggregate indicator. This signifies that the patient had at least three of four 'health risk' checks (blood pressure, BMI, cholesterol, and

For peer review only - http://bmjopen.bmj.com/site/about/guidelines.xhtml

glucose) documented within a three month period[29]. The date of the aggregate indicator is the date of the final check. In our analysis, patients are considered to have a 'current' annual review for twelve months after either an annual review (using the original Read coding), or from the date of this aggregate indicator.

The analysis includes three time-varying care quality indicator variables. For annual reviews, the base case is no annual review of physical health within the last twelve months. For care plans the base case is never having had a care plan. Two binary variables indicate whether a care plan or annual review has been recorded within the last twelve months ('current care plan' and 'current annual review'). If a patient receives a further care plan or annual review within the twelve-month window, the 'current' period is extended accordingly. A third variable indicates that the patient received a care plan more than twelve months ago ('expired care plan') but none in the last twelve months (mutually exclusive with 'current care plan').

Control variables

Using primary care data, we control for patients' age, gender, ethnicity, deprivation profile of their area of residence[30], year of SMI diagnosis, Charlson comorbidities[31], diagnosis of depression, history of smoking, and number of primary care attendances in the year prior to diagnosis. Their diagnosis of SMI was classified as "schizophrenia and other psychoses", "bipolar disorder and affective psychoses", or both, if the patient had both recorded. (See Supplementary Table 2 for a list of codes.) From HES data we capture number of hospital admissions in the year prior to diagnosis.

For peer review only - http://bmjopen.bmj.com/site/about/guidelines.xhtml

Statistical analysis

We estimate Cox survival models[32], examining duration from SMI diagnosis to each of the three outcomes separately: first A&E presentation, first SMI admission, and first ACSC admission. We follow each individual until the outcome of interest or until censoring. Censoring can occur because i) a patient dies, ii) registration with the practice ends, or iii) the study period ends (i.e. the patient is still registered on 31 March 2014).

In the Cox model the hazard of the outcome occurring is a function of the baseline hazard, the care indicators, and patient demographic and clinical characteristics. In the main model, we assume patient characteristics to be fixed at baseline, while the care indicators may vary over time. (Further details available in supplementary material.) We stratify on practice, so that the baseline hazard varies across practices, to allow for unobserved differences in patient populations and practice characteristics, and standard errors are adjusted for clustering at practice level to account for within-practice correlation. Coefficient estimates are presented as hazard ratios (HRs) with associated 95% confidence intervals (CIs), where values greater than 1 indicate an increase in the hazard of the outcome associated with a unit change in the explanatory variable, and vice versa for a HR below 1. All analyses were performed in Stata 14 (StataCorp LP, College Station, TX, US).

Sensitivity analyses

We undertake five tests of the sensitivity of our findings to alternate specifications of the model. First, we test the robustness of the annual review variable, which in our main analysis is the composite indicator constructed from both the QOF-specified

For peer review only - http://bmjopen.bmj.com/site/about/guidelines.xhtml

Read codes and the specific health checks that contribute to such reviews. We explore the impact of restricting this to the QOF-specified Read codes, which necessitates limiting the observation period to 2006/07 to 2010/11 (or 2007/08 to 2010/11 for the A&E outcome). Second, we assume the indicators expire after 15 months instead of 12 months. Third, instead of fixing the number of Charlson comorbidities at baseline, we allow this to increase over the period of observation if patients are diagnosed with new comorbidities. We lastly apply two alternate specifications of practice-level characteristics, instead of stratifying on practice. The first assumes no practice-level differences in baseline hazard, but includes practice characteristics (rurality and distance from closest acute hospital and inpatient psychiatric unit) as covariates. The second includes practice fixed effects as explanatory variables. Both models adjust the standard errors for clustering at the practice level as before.

Patient involvement

Two co-authors on the multidisciplinary team responsible for this study have lived experience of SMI. They contributed to the design of the research questions and methodological approach, interpretation of the findings, and writing the paper.

RESULTS

Study population

The full sample consists of 5,158 newly diagnosed patients with SMI from 213 practices in England with a total of 14,376 person-years observed before censoring (for A&E analysis 4,446 patients and a total of 10,952 person-years). On average patients were observed for 2.79 years. Most (67%) were still observed at the end of the study period, 23% exited because their registration with the practice ended during the study period, and 10% died. Table 1 presents the number of individuals with each

For peer review only - http://bmjopen.bmj.com/site/about/guidelines.xhtml

For peer review only

characteristic, and the contribution of those with each characteristic to the total time observed. (Equivalent tables are presented for each analysis sample in Supplementary Tables 3-5). There were more people per year diagnosed in later years of the study period, but each was observed for a shorter period, and therefore had a shorter window of opportunity to experience both the care quality indicators and the outcomes. Similarly, those aged over 65 made up 25% of the sample but only contributed 18% of the total time observed because they had a shorter than average period of observation (2.01 years). The median age at diagnosis in the sample was 46, and the most common physical comorbidities were respiratory disease, renal disease, cancer, and diabetes (further details available in Supplementary Table 6).

For peer review only

For peer review only - http://bmjopen.bmj.com/site/about/guidelines.xhtml

Table 1. Population characteristics: number and proportion of individuals in the sample, total person-years in the sample, and proportion of total person-years

	Individuals (% of total individuals)	Mean person-years (% of total person-years)
Total sample	5,158	14,736
Age at diagnosis		
18-35	1,540 (30%)	4,388 (30%)
36-45	1,008 (20%)	3,206 (22%)
46-55	736 (14%)	2,356 (16%)
56-65	561 (11%)	1,780 (12%)
>65	1,313 (25%)	2,646 (18%)
Index of multiple deprivation		
Quintile 1 (least deprived)	922 (18%)	2,535 (18%)
Quintile 2	980 (19%)	2,709 (19%)
Quintile 3	951 (18%)	2,613 (18%)
Quintile 4	1,150 (22%)	3,232 (22%)
Quintile 5 (most deprived)	1,155 (23%)	3,286 (23%)
Gender		
Male	2,430 (47%)	6,767 (47%)
Female	2,728 (53%)	7,609 (53%)
Ethnicity		
White	3,791 (73%)	10,546 (73%)
Black & minority ethnicities	1,367 (27%)	3,830 (27%)
Number of primary care contacts in year preceding diagnosis		
0-4	1,143 (22%)	3,393 (24%)
5-9	1,341 (26%)	4,032 (28%)
10-14	946 (18%)	2,598 (18%)
15-19	618 (12%)	1,619 (11%)
>=20	1,110 (22%)	2,735 (19%)
Number of hospital admissions in year preceding diagnosis		
0	2,886 (56%)	8,515 (59%)
1	1,250 (24%)	3,384 (23%)
2	544 (11%)	1,385 (10%)
3	478 (9%)	1,092 (8%)
Number of Charlson Index comorbidities at time of diagnosis		
0	3,047 (59%)	9,049 (63%)
1	1,384 (27%)	3,908 (27%)
2	446 (9%)	948 (7%)
3 or more	281 (5%)	471 (3%)
Comorbid depression at time of diagnosis		
History of depression	3,358 (65%)	9,648 (67%)

For peer review only - http://bmjopen.bmj.com/site/about/guidelines.xhtml

No recorded history of depression	1,800 (35%)	4,728 (33%)
Smoking status		
Current or ex-smoker	3,960 (77%)	11,059 (77%)
No recorded history of smoking	1,198 (23%)	3,317 (23%)
SMI diagnostic group		
Schizophrenia & other psychoses	2,884 (56%)	7,292 (51%)
Bipolar disorder & affective psychoses	2,078 (40%)	6,314 (44%)
Both	196 (4%)	770 (5%)
Financial year of diagnosis		
2006/07	617 (12%)	3,205 (22%)
2007/08	523 (10%)	2,381 (17%)
2008/09	573 (11%)	2,269 (16%)
2009/10	619 (12)	2,084 (14%)
2010/11	607 (12%)	1,611 (11%)
2011/12	734 (14%)	1,528 (11%)
2012/13	732 (14%)	969 (7%)
2013/14	753 (15%)	330 (2%)
Care plans (varying over time)^		
Current care plan (in last 12 mo.)		5,692 (40%)
Expired care plan (more than 12 mo. ago)		4,004 (28%)
No care plan at all		4,680 (32%)
Annual reviews (varying over time)^		
Current annual review (in last 12 mo.)		8,579 (60%)
No annual review in last 12mo		5,797 (40%)

Patient characteristics are fixed at baseline (date of diagnosis) except for care quality indicators, which vary over the study period.
^The three care plans categories are mutually exclusive (as are the two annual review categories) so each person's total time observed is the sum of these categories

For peer review only - http://bmjopen.bmj.com/site/about/guidelines.xhtml

Overall, 69% of the sample had a current care plan at least once, contributing 40% of the total time observed, while 72% of the sample had a current annual review at least once, contributing 60% of the total time observed, and 42% of the sample had an expired care plan at least once, contributing 28% of the total time observed. More detail on the annual rates of care plan and annual review indicators is available in supplementary material (Supplementary Figure 1).

Outcomes

Summary statistics for each of the three outcome variables are presented in Table 2. During the period of observation, 50% had an A&E presentation, 11% had an SMI admission, and 10% had an ACSC admission.

For peer review only - http://bmjopen.bmj.com/site/about/guidelines.xhtml

Table 2. Descriptive statistics for survival analysis, by outcome

Outcome	A&E presentation	SMI admission	ACSC admission
N individuals	4,446	5,158	5,158
Mean years observed	1.50	2.47	2.56
N with outcome (% of sample)	2,213 (50%)	562 (11%)	528 (10%)
Mean years observed to outcome*	1.08	1.20	1.68
N (% of events) with outcome occurring:			
within 12 months of CP (current CP)	756 (36%)	200 (35%)	171 (32%)
>12 months since CP (expired CP)	250 (11%)	77 (14%)	109 (21%)
with no CP	1,207 (54%)	285 (51%)	248 (47%)
within 12 months of AR (current AR)	1,024 (46%)	263 (47%)	301 (57%)
with no current annual in last 12 months	1,189 (54%)	299 (53%)	227 (43%)

The period of observation is shorter in the outcome-specific samples than the full study observation period because the patient exits observation once the first outcome event occurs.
*Mean years observed from diagnosis to outcome for those who experienced the outcome.
CP: Care plan. AR: Annual review of physical health.
A&E presentation: Presentation of any cause to the Accident & Emergency department
SMI admission: Inpatient admission with a primary diagnosis of serious mental illness (ICD-10 codes F20-F31)
ACSC admission: Inpatient admission with a diagnosis of an ambulatory care-sensitive condition (see Supplementary Table 1 for a list of conditions)

For peer review only

For peer review only - http://bmjopen.bmj.com/site/about/guidelines.xhtml

Table 3: Main model - full results with A&E presentation as the outcome

		Main model HR	(95% CI)
Current annual review		0.962	(0.856-1.080)
Current care plan		0.866*	(0.766-0.979)
Expired care plan		0.808*	(0.672-0.972)
Age at diagnosis			
	18-35 (base)		
	36-45	0.806*	(0.657-0.988)
	46-55	0.619***	(0.507-0.755)
	56-65	0.582***	(0.460-0.738)
	>=66	1.098	(0.917-1.314)
Male		1.016	(0.861-1.199)
Age at diagnosis*Male			
	18-35*Male (base)		
	36-45*Male	0.919	(0.694-1.216)
	46-55*Male	1.215	(0.911-1.614)
	56-65*Male	1.432*	(1.037-1.978)
	≥66*Male	0.939	(0.727-1.203)
SMI diagnosis group			
Bipolar disorder or affective psychosis (base)			
Schizophrenia or other psychosis		1.103	(0.997-1.220)
Both		1.119	(0.888-1.411)
IMD			
	1st quintile (base)		
	2nd quintile	0.955	(0.804-1.133)
	3rd quintile	1.100	(0.918-1.319)
	4th quintile	1.178	(0.978-1.419)
	5th quintile	1.196	(0.985-1.453)
Ethnicity white		1.277***	(1.149-1.421)
Number of admissions within 12 months prior to diagnosis			
	0 (base)		
	1	1.174**	(1.050-1.313)
	2	1.190*	(1.028-1.377)
	3 or more	1.500***	(1.268-1.775)
Number of primary care contacts within 12 months prior to diagnosis			
	<5 (base)		
	5-9	1.219**	(1.056-1.407)
	10-14	1.334***	(1.165-1.528)
	15-19	1.714***	(1.459-2.013)
	>=20	1.861***	(1.592-2.176)
Current or ex-smoker		1.101	(0.983-1.233)
Number of Charlson Index comorbidities		1.111***	(1.052-1.174)

For peer review only - http://bmjopen.bmj.com/site/about/guidelines.xhtml

	Main model HR	(95% CI)
Diagnosis of depression	1.005	(0.904-1.118)
Year of diagnosis		
2007 (base)		
2008	1.263*	(1.040-1.532)
2009	1.383***	(1.149-1.665)
2010	1.644***	(1.356-1.994)
2011	1.551***	(1.244-1.933)
2012	1.385**	(1.105-1.737)
2013	1.266	(0.990-1.614)
2014	0.970	(0.557-1.691)
Practice list size	1.000	(1.000-1.000)
Number of patients	4,446	

*p<0.05, **p<0.01, ***p<0.001

For peer review only

Results of survival analysis

Results showing the association of the three key explanatory variables with hazard (HR) of each of the outcomes are presented in Figure 1. HRs for control variables are presented in Table 3 with A&E as the outcome.

[Figure 1 about here]

The hazard of A&E presentation is 13% lower for patients who have a current care plan (HR 0.87, 95% CI 0.77-0.98) and 19% lower for those with an expired care plan (HR 0.81, 95% CI 0.67-0.97), both compared with those who have had no care plan at all. There is no statistically significant association with annual reviews of physical health (HR 0.96, 95% CI 0.86-1.08). The hazard of first ACSC admission after SMI diagnosis is 23% lower for those with a current care plan (HR 0.77, 95% CI 0.60-0.99), but there is no statistically significant association with expired care plans or annual reviews. Neither indicator shows a statistically significant association with the hazard of SMI admission.

Results of sensitivity testing (detailed in Supplementary Tables 7-9) show no statistically significant association between annual reviews of physical health and any of the outcomes under all assumptions tested. The association between care plans and A&E presentations remained essentially unchanged, except that the association with current care plans became non-significant (as confidence intervals widened) when the analysis was limited to 2007/08 – 2010/11, and similarly with expired care plans when the indicators were assumed to be effective (current) for 15 months instead of 12. There remained no statistically significant association between the indicators and

For peer review only - http://bmjopen.bmj.com/site/about/guidelines.xhtml

SMI admissions under the varying assumptions. The association between ACSC admission and current care plans was less robust to changing assumptions, losing statistical significance under most alternate specifications and changing signs when the period of analysis was limited to 2007/08 – 2010/11.

DISCUSSION

Principal findings

We find that, among patients with SMI, those with a care plan documented in primary care in the last 12 months have a 13% lower hazard of A&E presentation than those without a care plan, and a 23% lower hazard of admission to hospital for ACSC (the types of condition thought to be amenable to primary care). Those with a care plan documented more than 12 months ago also had a lower hazard of A&E presentation (19% lower than those with no care plan). We find no such association between annual reviews of physical health and the hazard of any of the outcomes, nor between care plans and admissions for SMI.

Strengths and weaknesses

Our study makes a number of important contributions to the evidence base, being (to our knowledge) the first to use linked patient-level data to investigate the relationship between incentivized primary care quality and hospital care for people with SMI in the English NHS. By linking datasets, we track individual patients across primary care and hospital settings over a number of years, and determine whether the primary care indicator precedes the hospital utilisation. Our survival analysis exploits this, providing more robust estimates of the association than would be possible with aggregate data.

For peer review only - http://bmjopen.bmj.com/site/about/guidelines.xhtml

There are a number of limitations to our study. The measures of health status and healthcare utilisation prior to diagnosis of SMI may not fully depict the complexities of health status, including severity of SMI, and this may confound the results. However, our findings were robust to whether the number of comorbidities was fixed at baseline or allowed to vary over the period of observation. The outcomes we analyse are measures of hospital utilisation, which are imperfect proxies for health outcomes. Our finding of a negative association between care plans and A&E presentations or ACSC admissions may reflect the presence of unobserved factors that could contribute to the observed association without implying causality. We lack information on the clinical circumstances leading to a care plan or annual review being recorded, and on its content, quality and appropriateness. In the period preceding hospital attendance, it is possible that care planning could be superseded by acute management of physical or mental health conditions. Patients who have less insight or lower levels of self-care may be less likely to seek regular primary care, and therefore less likely to have care plans documented, but may also be more likely to seek A&E care[33 34]. Conversely, our findings could underestimate any real effect of care plans on A&E presentations if other unobserved factors contribute to a (non-causal) positive association, such as care plans being triggered by patients attending their GP for problems that will eventually need hospital care.

We restricted the sample to patients registered with the same practice for the year preceding diagnosis in order to analyse a group of patients at a similar clinical stage, and allow us to include measures of historical utilization and medical history. However, the restriction may have excluded individuals with more severe SMI if this

For peer review only - http://bmjopen.bmj.com/site/about/guidelines.xhtml

led them to move practice. In addition, the sample may include some patients who were not newly diagnosed, if their diagnostic information had not been transferred to a new practice, or to a new electronic recording system.

The collection of HES A&E commenced on an experimental basis in April 2007, and captured 62% of national A&E attendances in the first financial year. The experimental label was lifted by April 2013 after capture had increased to over 80%. However, there is no reason to consider that the capture of attendances is related to whether a patient had a care plan or physical review, meaning our comparative analysis should not be adversely impacted.

Comparison with other studies

There is limited, mixed evidence about the relationship between objective measures of primary care quality and A&E attendance. Baker, et al.[35] found that higher performance on the QOF overall was not a predictor of area-level A&E attendances, while subjective assessment of higher primary care quality has been found to be associated with lower utilisation of A&E[36 37]. Using practice-level data, Gutacker, et al.[21] found that higher achievement on the annual review indicator was associated with a higher rate of SMI admissions. One possible explanation was that patients had QOF indicators documented after a hospital admission. Our analysis, which captures the sequence of events and finds no association between annual reviews and hazard of SMI admission, supports that explanation. Harrison, et al.[38] found, using aggregate data, that the introduction of the QOF was associated with a decrease in unplanned admissions for incentivised ACSCs, consistent with our finding that care plans were associated with a reduced risk of ACSC admissions. Wilson, et al.[39] found, using

For peer review only - http://bmjopen.bmj.com/site/about/guidelines.xhtml

individual-level data, that while introduction of QOF incentives increased detection of cardiovascular risk factors in patients with SMI, there was not necessarily any change in management. This is consistent with our finding no evidence of improved health outcomes (or their proxies, unplanned hospital utilisation) associated with annual reviews of physical health.

Interpretation of results and implications for clinicians and policymakers

The association of care plans with reduced hazard of A&E presentations and ACSC admissions might suggest that care plans help patients avoid hospital for conditions that do not require hospital care. The documentation of patients' current health status, early warning signs, triggers, social support needs, co-ordination arrangements with secondary care, and preferred course of action in the event of a clinical relapse could improve the management of the patient's health overall and prevent deterioration, reducing the need for urgent care represented by ACSC admissions and A&E presentations. It may also direct patients into appropriate services during periods of deterioration, and thereby avert hospital use.

Increasing demand for A&E care, especially for problems that could be managed elsewhere, is a policy focus in the UK NHS and in other health care systems[40]. Patients with SMI, in particular, can require high resource input when attending A&E[41 42]. The potential for care planning by GPs to reduce A&E attendances in this patient population could therefore be an important finding for policymakers, suggesting that continued incentivisation of this activity in primary care may help reduce demand on secondary care services.

For peer review only - http://bmjopen.bmj.com/site/about/guidelines.xhtml

The associations of both ‘current’ and ‘expired’ care plans with lower hazard of A&E attendance suggest that an older care plan can still be beneficial. However, the finding that when care plans are considered effective for 15 months rather than 12 months, only ‘current’ care plans are associated with reduced A&E attendance suggests that there are limits to the duration of impact. The lack of association between annual reviews of physical health and unplanned hospital care may suggest that effectiveness of a GP in managing a patient’s health, and preventing the need for A&E or hospital care, may be unaffected by whether or not annual reviews or health checks are recorded.

Unanswered questions and future research

This study did not account for care provided by patients’ key workers or a crisis resolution and home treatment team, which may provide an alternative to both hospital admission and primary care. The modelling approach does adjust for differences across practices, which may help control for some of these local effects. To address this more adequately, the Mental Health Services Dataset, which can now be linked to CPRD primary care data, could be used to cover mental health care delivered in the community.

Conclusion

Provider incentive schemes are increasingly popular levers for improving value and quality in healthcare worldwide, but evidence is needed on the effectiveness of incentivised activities. Despite the vulnerability of people with SMI to poor health outcomes, relatively few studies have examined the impact of care quality indicators in this population. This study addresses that evidence gap, and advances our

For peer review only - http://bmjopen.bmj.com/site/about/guidelines.xhtml

understanding of how primary care can influence utilisation of hospital care in this patient population. We find that care plans are associated with reduced risk of A&E presentation and hospital admissions for conditions amenable to primary care, supporting the hypothesis that this type of care incentivized under the QOF is achieving at least some of its policy objectives and is worth maintaining.

For peer review only

For peer review only - http://bmjopen.bmj.com/site/about/guidelines.xhtml

Author contributions

JR, PK, NG, CK, TD, AM, NR, HG, MG, TK, NS, SG, CD, LA, RW and RJ contributed to the design of the research questions, analytical approach, and interpretation of the findings. JR produced a first draft of the paper and contributed to and PK, NG, CK, TD, AM, NR, HG, MG, TK, NS, SG, CD, LA, RW and RJ approved the final manuscript.

Data sharing

Due to the sensitive and confidential nature of the data used for this analysis, and the permissions required to access it, the dataset is not publicly available.

Ethical approval

The study protocol was approved by the Independent Scientific Advisory Committee of the Clinical Practice Research Datalink (protocol number 15_213R2A2).

Acknowledgements

This research is funded by the National Institute for Health Services & Delivery Research program (project number 135440). The views and opinions expressed are those of the authors and do not necessarily reflect those of the HS&DR program, NIHR, NHS or the Department of Health. We are grateful to the researchers who extracted and provided the CPRD data. We would like to thank all members of our Scientific Steering Committee (SSC) for their invaluable support and feedback on this study. We are grateful to discussants and participants at the following conferences and seminars for their valuable feedback: Thirteenth Workshop on Costs and Assessment in Psychiatry: Mental health policy and economics, Venice, Italy; International Health

For peer review only

For peer review only - http://bmjopen.bmj.com/site/about/guidelines.xhtml

Policy Conference, LSE, London; Supportive care, Early Diagnosis and Advanced disease (SEDA) Research Group, Hull York Medical School (HYMS), Hull; University of Toronto, Centre for Addiction and Mental Health (CAMH) and the Canadian Centre for Health Economics (CCHE), Toronto, Canada.

Endnotes

1. We use a twelve-month window rather than the calendar or financial year.
2. We consider any patients with unrelated hospitalisation (e.g. a planned admission for surgery) as having zero hazard of A&E presentation and SMI or ACSC admission during the time they are hospitalised.

Figure 1 legend

Figure 1. Association of care quality indicators with each outcome.

CP: Care plan

AR: Annual review of physical health

For peer review only

For peer review only - http://bmjopen.bmj.com/site/about/guidelines.xhtml

REFERENCES

1. Murray CJ, Richards MA, Newton JN, et al. UK health performance: findings of the Global Burden of Disease Study 2010. The Lancet 2013;**381**(9871):997-1020.
2. Vigo D, Thornicroft G, Atun R. Estimating the true global burden of mental illness. The Lancet Psychiatry 2016;**3**(2):171-78.
3. Osborn DP, Levy G, Nazareth I, et al. Relative risk of cardiovascular and cancer mortality in people with severe mental illness from the United Kingdom's General Practice Research Database. Archives of General Psychiatry 2007;**64**(2):242-49.
4. Miller B, Paschall C, Svendsen D. Mortality and medical comorbidity among patients with SMI. Psychiatric Services 2006;**57**(10):1482-87.
5. Wahlbeck K, Westman J, Nordentoft M, et al. Outcomes of Nordic mental health systems: life expectancy of patients with mental disorders. The British Journal of Psychiatry 2011;**199**(6):453-58.
6. Mental Health Statistics team HaSCIC. HES-MHMDS Data Linkage Report, Additional analysis - 2011-12, England (Experimental statistics. Leeds: Health and Social Care Information Centre, 2013.
7. Hackman AL, Goldberg RW, Brown CH, et al. Use of emergency department services for somatic reasons by people with serious mental illness. Psychiatric services 2006;**57**(4):563-66.
8. Merrick EL, Perloff J, Tompkins CP. Emergency department utilization patterns for Medicare beneficiaries with serious mental disorders. Psychiatric services 2010;**61**(6):628-31.
9. Schoenbaum M, Sutherland JM, Chappel A, et al. Twelve-Month Health Care Use and Mortality in Commercially Insured Young People With Incident Psychosis in the United States. Schizophrenia Bulletin 2017;**43**(6):1262-72.
10. Lally J, Wong YL, Shetty H, et al. Acute hospital service utilization by inpatients in psychiatric hospitals. Gen Hosp Psychiat 2015;**37**(6):577-80.
11. Reilly S, Planner C, Hann M, et al. The role of primary care in service provision for people with severe mental illness in the United Kingdom. Plos One 2012;**7**(5):e36468.
12. World Health Organization. Quality improvement for mental health: mental health policy and service guidance package. Geneva: WHO, 2003.
13. World Health Organization. QualityRights tool kit to assess and improve quality and human rights in mental health and social care facilities. Geneva: WHO, 2012.
14. World Health Organisation. Paying for performance in health care: Implications for health system performance and accountability. In: Cashin C, Chi Y-L, Smith P, et al., eds. European Observatory on Health Systems and Policies. Maidenhead, UK: Open University Press, 2014.
15. Eijkenaar F, Emmert M, Scheppach M, et al. Effects of pay for performance in health care: a systematic review of systematic reviews. Health Policy 2013;**110**(2):115-30.
16. Flodgren G, Eccles MP, Shepperd S, et al. An overview of reviews evaluating the effectiveness of financial incentives in changing healthcare professional behaviours and patient outcomes. The Cochrane Library 2011.
17. Doran T, Maurer KA, Ryan AM. Impact of Provider Incentives on Quality and Value of Health Care. Annual Review of Public Health 2017;**38**:449-65.
18. Roland M. Linking physicians' pay to the quality of care—a major experiment in the United Kingdom. New England Journal of Medicine 2004;**351**:1448-54.
19. National collaborating Centre for Mental Health. Psychosis and Schizophrenia in Adults: Treatment and Management. NICE Clinical Guideline 178, 2014.

For peer review only - http://bmjopen.bmj.com/site/about/guidelines.xhtml

20. National Collaborating Centre for Mental Health. Bipolar disorder: The assessment and management of bipolar disorder in adults, children and young people in primary and secondary care. Leicester UK: The British Psychological Society 2014.
21. Gutacker N, Mason AR, Kendrick T, et al. Does the quality and outcomes framework reduce psychiatric admissions in people with serious mental illness? A regression analysis. Bmj Open 2015;**5**(4):e007342.
22. Purdy S, Griffin T, Salisbury C, et al. Ambulatory care sensitive conditions: terminology and disease coding need to be more specific to aid policy makers and clinicians. Public Health 2009;**123**(2):169-73.
23. Wykes TL, Lee AA, McKibbin CL, et al. Self-Efficacy and Hemoglobin A1C Among Adults With Serious Mental Illness and Type 2 Diabetes: The Roles of Cognitive Functioning and Psychiatric Symptom Severity. Psychosomatic Medicine 2016;**78**(3):263-70.
24. Chen SR, Chien YP, Kang CM, et al. Comparing self-efficacy and self-care behaviours between outpatients with comorbid schizophrenia and type 2 diabetes and outpatients with only type 2 diabetes. Journal of Psychiatric and Mental Health Nursing 2014;**21**(5):414-22.
25. Herrett E, Gallagher AM, Bhaskaran K, et al. Data resource profile: clinical practice research datalink (CPRD). Int J Epidemiol 2015;**44**(3):827-36.
26. Booth N. What are the Read codes? Health Libraries Review 1994;**11**(3):177-82.
27. World Health Organization, editor. *The Tenth Revision of the International Classification of Diseases and Related Health Problems (ICD-10)*. Geneva: World Health Organisation, 1992.
28. Bardsley M, Blunt I, Davies S, et al. Is secondary preventive care improving? Observational study of 10-year trends in emergency admissions for conditions amenable to ambulatory care. BMJ Open 2013;**3**(1):e002007.
29. Chang KC-M, Soljak M, Lee JT, et al. Coverage of a national cardiovascular risk assessment and management programme (NHS Health Check): Retrospective database study. Preventive Medicine 2015;**78**:1-8.
30. McLennan D, Barnes H, Noble M, et al. The English indices of deprivation 2010. London: Department for Communities and Local Government 2011.
31. Khan NF, Perera R, Harper S, et al. Adaptation and validation of the Charlson Index for Read/OXMIS coded databases. Bmc Fam Pract 2010;**11**(1):1.
32. Cox DR. Regression Models and Life-Tables. Journal of the Royal Statistical Society Series B (Methodological) 1972;**34**(2):187-220.
33. Yen C-F, Yeh M-L, Chen C-S, et al. Predictive value of insight for suicide, violence, hospitalization, and social adjustment for outpatients with schizophrenia: a prospective study. Comprehensive psychiatry 2002;**43**(6):443-47.
34. Yen C-F, Chen C-S, Yen J-Y, et al. The predictive effect of insight on adverse clinical outcomes in bipolar I disorder: A two-year prospective study. Journal of Affective Disorders 2008;**108**(1):121-27.
35. Baker R, Bankart MJ, Rashid A, et al. Characteristics of general practices associated with emergency-department attendance rates: a cross-sectional study. BMJ Quality & Safety 2011;**20**(11):953-58.
36. Wong CY, Hall J. Does patients' experience of general practice affect the use of emergency departments? Evidence from Australia. Health Policy 2018;**122**(2):126-33.
37. Xin H, Kilgore ML, Sen B, et al. Can Nonurgent Emergency Department Care Costs be Reduced? Empirical Evidence from a U.S. Nationally Representative Sample. The Journal of Emergency Medicine 2015;**49**(3):347-54.

For peer review only

For peer review only - http://bmjopen.bmj.com/site/about/guidelines.xhtml

38. Harrison MJ, Dusheiko M, Sutton M, et al. Effect of a national primary care pay for performance scheme on emergency hospital admissions for ambulatory care sensitive conditions: controlled longitudinal study. Bmj 2014;**349**:g6423.
39. Wilson CL, Rhodes KM, Payne RA. Financial incentives improve recognition but not treatment of cardiovascular risk factors in severe mental illness. PLOS ONE 2017;**12**(6):e0179392.
40. Berchet C. Emergency Care Services. 2015.
41. Broadbent M, Jarman H, Berk M. Emergency department mental health triage scales improve outcomes. Journal of Evaluation in Clinical Practice 2004;**10**(1):57-62.
42. Eric P. Slade PD, Lisa B. Dixon MD, M.P.H. ,, Sarah Semmel BA. Trends in the Duration of Emergency Department Visits, 2001–2006. Psychiatric Services 2010;**61**(9):878-84.

For peer review only

For peer review only - http://bmjopen.bmj.com/site/about/guidelines.xhtml

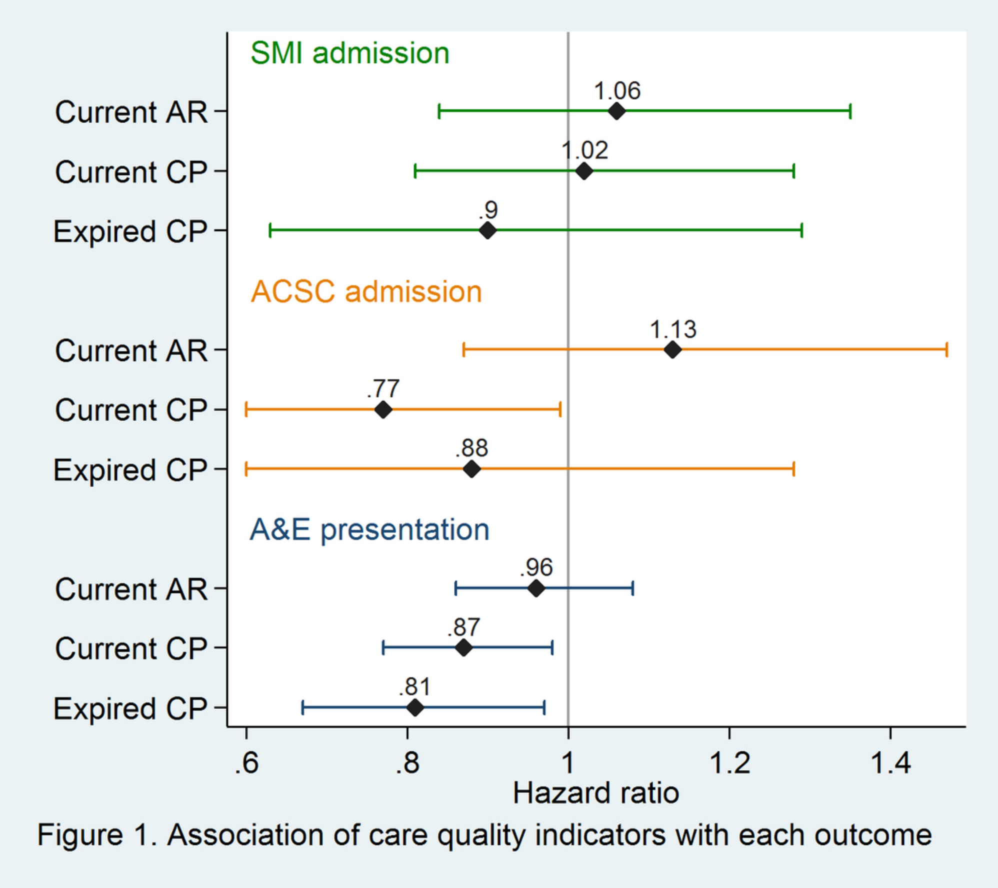

Figure 1. Association of care quality indicators with each outcome.
CP: Care plan
AR: Annual review of physical health

93x83mm (300 x 300 DPI)

Supplementary Table 1: Conditions classified as ambulatory care sensitive admissions^

Condition	ICD-10 codes used to identify the condition#
Angina	I10 I24.0 I24.8 I24.9
Asthma	J45 J46
Chronic obstructive pulmonary disease	J41 J42 J43 J44 J47<br>First diagnosis: J20 + Second diagnosis one of: J41 J42 J43 J44 J47
Congestive heart failure	I50 I11.0 J81
Diabetes (in any diagnosis field)	E10.0 E10.1 E10.2 E10.3 E10.4 E10.5 E10.6 E10.7 E10.8<br>E11.0 E10.1 E11.2 E10.3 E11.4 E10.5 E11.6 E10.7 E11.8<br>E12.0 E10.1 E12.2 E10.3 E12.4 E10.5 E12.6 E10.7 E12.8<br>E13.0 E10.1 E13.2 E10.3 E13.4 E10.5 E13.6 E10.7 E13.8<br>E14.0 E10.1 E14.2 E10.3 E14.4 E10.5 E14.6 E10.7 E14.8
Epilepsy	G40 G41 O15 R56
Hypertension	I10 I11.9
Anaemia	D50.0 D50.8 D50.9
Cellulitis	L03 L04 L08 L88 L98.0 L98.3
Dehydration	E86
Dental	A69.0 K02 K03 K04 K05 K06 K08 K09.8 K09.9 K12 K13
Ear, nose and throat infections	H66 H67 J02 J03 J06 J31.2
Gangrene (in any diagnosis field)	R02
Gastroenteritis	K52.2 K52.8 K52.9

For peer review only

Nutritional deficiencies	E40 E41 E42 E43 E55 E64.3
Perforated or bleeding ulcer	K25.0 K25.1 K25.2 K25.4 K25.5 K25.6<br>K26.0 K26.1 K26.2 K26.4 K26.5 K26.6<br>K27.0 K27.1 K27.2 K27.4 K27.5 K27.6<br>K28.0 K28.1 K28.2 K28.4 K28.5 K28.6
Urinary tract infection or pyelonephritis	N10 N11 N12 N13.6 N39.0
Influenza (in any diagnosis field, exclude secondary diagnosis of D57)	J10 J11
Pneumonia (in any diagnosis field, exclude secondary diagnosis of D57)	J13 J14 J15.3 J15.4 J15.7 J15.9 J16.8 J18.1 J18.8
Tuberculosis	A15 A16 A19
Other vaccine-preventable diseases (in any diagnosis field)	A35 A36 A37 A80 B05 B06 B16.1 B16.9 B18.0 B18.1<br>B26 G00.0 M01.4

^Based on Bardsley et al. (2013)

#Based on the first diagnosis field in HES data unless otherwise specified.

For peer review only - http://bmjopen.bmj.com/site/about/guidelines.xhtml

Supplementary Table 2: Read codes used to identify SMI diagnostic categories

Category	Read codes used to identify diagnostic category
Schizophrenia and other psychoses	E100.00 E100.11 E100000 E100100 E100200 E100300 E100400 E100500 E100z00 E101.00 E101000 E101400 E101500 E101z00 E102.00 E102000 E102100 E102500 E102z00 E103.00 E103000 E103200 E103300 E103400 E103500 E103z00 E104.00 E105.00 E105000 E105200 E105500 E105z00 E106.00 E107.00 E107.11 E107000 E107100 E107200 E107300 E107400 E107500 E107z00 E10y.00 E10y.11 E10y000 E10y100 E10yz00 E10z.00 E120.00 E121.00 E122.00 E123.00 E123.11 E12y.00 E12y000 E12yz00 E12z.00 E13..00 E13..11 E131.00 E132.00 E133.00 E133.11 E134.00 E13y.00 E13y100 E13yz00 E13z.00 E13z.11 E1z..00 E212200 Eu20.00 Eu20000 Eu20011 Eu20100 Eu20111 Eu20200 Eu20211 Eu20212 Eu20213 Eu20214 Eu20300 Eu20311 Eu20400 Eu20500 Eu20511 Eu20600 Eu20y00 Eu20y12 Eu20y13 Eu20z00 Eu21.00 Eu21.11 Eu21.12 Eu21.13 Eu21.14 Eu21.15 Eu21.16 Eu21.17 Eu21.18 Eu22.00 Eu22000 Eu22011 Eu22012 Eu22013 Eu22014 Eu22015 Eu22100 Eu22111 Eu22200 Eu22300 Eu22y00 Eu22y11 Eu22y12 Eu22y13 Eu22z00 Eu23.00 Eu23000 Eu23011 Eu23012 Eu23100 Eu23112 Eu23200 Eu23211 Eu23212 Eu23214 Eu23300 Eu23312 Eu23y00 Eu23z00 Eu23z11 Eu23z12 Eu24.00 Eu24.12 Eu24.13 Eu25.00 Eu25000 Eu25011 Eu25012 Eu25100 Eu25111 Eu25112 Eu25200 Eu25212 Eu25y00 Eu25z00 Eu25z11 Eu26.00 Eu2y.00 Eu2y.11 Eu2z.00 Eu2z.11 Eu44.14

For peer review only

Bipolar disorder and affective psychoses	E11..00 E11..12 E110.00 E110.11 E110000 E110100 E110200 E110300 E110400 E110600 E110z00 E111.00 E111000 E111100 E111200 E111300 E111400 E111500 E111600 E111z00 E112400 E113400 E114.00 E114.11 E114000 E114100 E114200 E114300 E114400 E114500 E114600 E114z00 E115.00 E115.11 E115000 E115100 E115200 E115300 E115400 E115500 E115600 E115z00 E116.00 E116000 E116100 E116200 E116300 E116400 E116500 E116600 E116z00 E117.00 E117000 E117100 E117200 E117300 E117400 E117500 E117600 E117z00 E11y.00 E11y000 E11y100 E11y300 E11yz00 E11z.00 E11z000 E11zz00 E130.00 E130.11 E13y000 Eu30.00 Eu30.11 Eu30000 Eu30100 Eu30200 Eu30211 Eu30212 Eu30y00 Eu30z00 Eu30z11 Eu31.00 Eu31.11 Eu31.12 Eu31.13 Eu31000 Eu31100 Eu31200 Eu31300 Eu31400 Eu31500 Eu31600 Eu31700 Eu31800 Eu31900 Eu31911 Eu31y00 Eu31y11 Eu31y12 Eu31z00 Eu32300 Eu32311 Eu32312 Eu32313 Eu32314 Eu32800 Eu33213 Eu33300 Eu33311 Eu33312 Eu33313 Eu33314 Eu33315 Eu33316 Eu3z.11

For peer review only

Rates of care indicators over the study period

Supplementary Figure 1 shows the rate of care indicators recorded by GP practices per patient-year for each financial year in our sample. This rate is the number of times an indicator is recorded in that year across all patients, divided by the number of patient-years of observation in that financial year (which takes into account partial years for patients who were not observed for the full financial year). For example, in 2007/08 the rate of annual reviews (composite measure) recorded was 1.06 per patient-year.

The CP indicator shows a decline over the study period until 2012/13, when there was an increase in the threshold of achievement required to attain the maximum payment. The original AR indicator shows a marked drop-off after it ceased to be incentivized in 2010/11, while the aggregate indicator composed of the 'health risk' checks shows less marked change but a small increase after the specific components began to be incentivized in 2011/12. The composite of these two AR indicators shows little change in response to the QOF rule changes.

Supplementary Figure 1: Rate of care indicators per patient-year

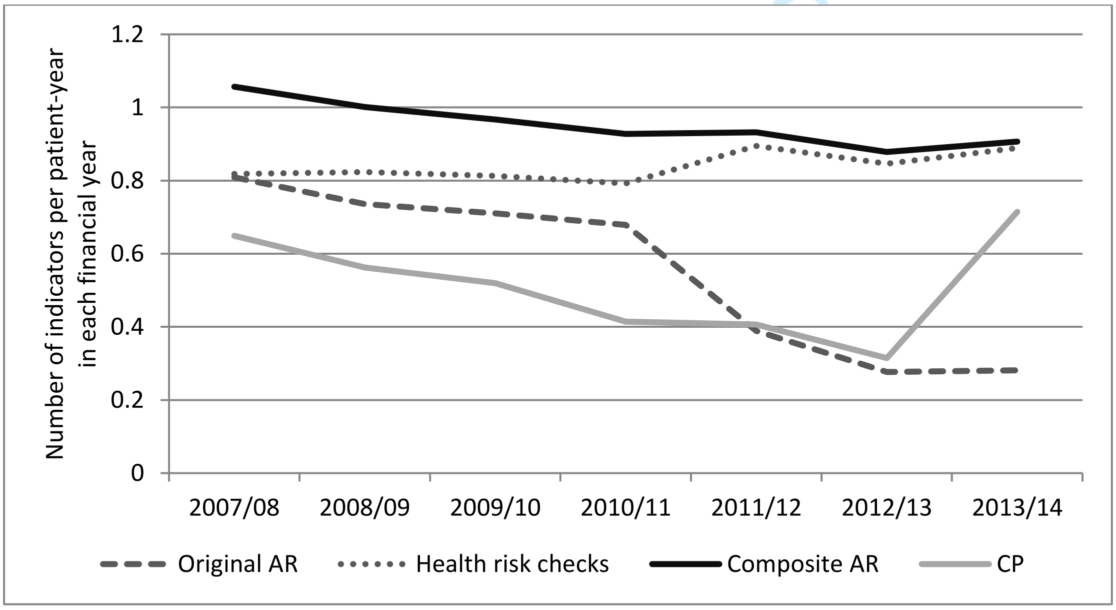

For peer review only - http://bmjopen.bmj.com/site/about/guidelines.xhtml

Survival analysis

We estimate the following hazard function for each outcome, which is the product of the baseline hazard, $\lambda_0(t)$, and an exponentiated linear function of patients' characteristics fixed at baseline (X_i) and care quality indicators for patient *i* at practice g at time *t*:

$$h_{ig}(t) = \lambda_{0_g}(t)\exp\{\beta_1 X_{ig1} + \cdots + \beta_k X_{igk} + \alpha_1 CP_{ig}^{cur.}(t) + \alpha_2 CP_{ig}^{exp.}(t) + \alpha_3 AR_{ig}(t)\} \quad (1)$$

The care quality indicators are represented by three time-varying variables:

$$CP_i^{cur.}(t) = \begin{cases} = 1 \text{ if a care plan has been documented in the last 12 months} \\ = 0 \text{ if a care plan has not been documented in the last 12 months} \end{cases}$$

$$CP_i^{exp.}(t) = \begin{cases} = 1 \text{ if a care plan has been documented before the last 12 months} \\ = 0 \text{ if a care plan has never been documented} \end{cases}$$

$$AR_i(t) = \begin{cases} = 1 \text{ if an annual review has been documented in the last 12 months} \\ = 0 \text{ if an annual review has not been documented in the last 12 months} \end{cases}$$

λ_{0_g}: the baseline hazard is allowed to vary by practice

For peer review only - http://bmjopen.bmj.com/site/about/guidelines.xhtml

Supplementary Table 3: Distribution of person-years observed across patient characteristics in sample for analysis of A&E presentations

Sample for A&E presentations	Person-years overall	Care plans: % of row person-years with current CP	Care plans: % of row person-years with expired CP	Care plans: % of row person-years with no CP	Annual reviews: % of row person-years with current AR	Annual reviews: % of row person-years with no current AR
Sample overall	6,654	40%	20%	40%	56%	44%
Age at diagnosis						
18-35	1,901	41%	19%	40%	49%	51%
36-45	1,496	41%	20%	39%	53%	47%
46-55	1.233	41%	23%	36%	62%	38%
56-65	842	39%	20%	41%	62%	38%
>65	1,181	37%	16%	47%	58%	42%
Index of multiple deprivation						
Quintile 1 (least deprived)	1,246	43%	21%	36%	59%	41%
Quintile 2	1,326	41%	185	41%	58%	42%
Quintile 3	1,188	37%	20%	43%	52%	48%
Quintile 4	1,464	40%	20%	40%	55%	45%
Quintile 5 (most deprived)	1,430	39%	19%	42%	54%	46%
Gender						
Male	3,131	41%	20%	39%	54%	46%
Female	3,522	39%	20%	41%	57%	43%
Ethnicity						
White	4,620	40%	19%	41%	56%	44%
Black & minority ethnicities	2,034	40%	21%	39%	54%	46%
Number of primary care contacts in year preceding diagnosis						
0-4	1,726	38%	22%	40%	49%	51%
5-9	1,894	42%	19%	39%	56%	44%
10-14	1,255	41%	18%	41%	58%	42%

For peer review only - http://bmjopen.bmj.com/site/about/guidelines.xhtml

Sample for A&E presentations	Person-years overall	Care plans: % of row person-years with current CP	Care plans: % of row person-years with expired CP	Care plans: % of row person-years with no CP	Annual reviews: % of row person-years with current AR	Annual reviews: % of row person-years with no current AR
15-19	694	40%	18%	42%	59%	41%
>=20	1,085	40%	18%	42%	61%	39%
Number of hospital admissions in year preceding diagnosis						
0	4,119	38%	20%	42%	55%	45%
1	1,510	43%	21%	36%	56%	44%
2	611	46%	18%	36%	62%	38%
3	414	40%	19%	41%	57%	43%
Number of Charlson Index comorbidities at time of diagnosis						
0	4,346	40%	20%	40%	54%	46%
1	1,702	41%	20%	39%	58%	42%
2	405	34%	15%	51%	61%	39%
3 or more	201	31%	16%	52%	64%	36%
Comorbid depression at time of diagnosis						
History of depression	4,409	41%	19%	40%	56%	44%
No recorded history of depression	2,245	39%	21%	40%	54%	46%
Smoking status						
Current or ex-smoker	4,976	39%	19%	42%	56%	44%
No recorded history of smoking	1,678	42%	21%	37%	56%	44%
SMI diagnostic group						
Schizophrenia & other psychoses	3,427	40%	20%	40%	54%	46%
Bipolar disorder & affective psychoses	2,887	40%	19%	41%	57%	43%
Both	341	47%	22%	31%	61%	39%
Financial year of diagnosis						

For peer review only - http://bmjopen.bmj.com/site/about/guidelines.xhtml

Sample for A&E presentations	Person-years overall	Care plans			Annual reviews	
		% of row person-years with current CP	% of row person-years with expired CP	% of row person-years with no CP	% of row person-years with current AR	% of row person-years with no current AR
2007/08	1,407	43%	30%	27%	65%	35%
2008/09	1,265	40%	28%	32%	61%	39%
2009/10	1,159	38%	23%	39%	59%	41%
2010/11	931	41%	18%	41%	57%	43%
2011/12	937	38%	9%	53%	49%	51%
2012/13	684	39%	2%	59%	40%	60%
2013/14	272	39%	0	61%	29%	71%

For peer review only

Supplementary Table 4: Distribution of person-years observed across patient characteristics in sample for analysis of SMI admissions

Sample for SMI admissions	Person-years overall	Care plans			Annual reviews	
		% of row person-years with current CP	% of row person-years with expired CP	% of row person-years with no CP	% of row person-years with current AR	% of row person-years with no current AR
Sample overall	12,724	39%	27%	34%	59%	41%
Age at diagnosis						
18-35	3,825	40%	26%	34%	51%	29%
36-45	2,886	39%	29%	32%	57%	43%
46-55	2,080	40%	27%	33%	65%	35%
56-65	1,570	38%	29%	33%	68%	32%
>65	2,362	35%	24%	41%	63%	37%
Index of multiple deprivation						
Quintile 1 (least deprived)	2,255	39%	27%	34%	60%	40%
Quintile 2	2,394	40%	26%	34%	60%	40%
Quintile 3	2,327	35%	29%	36%	56%	44%
Quintile 4	2,874	40%	27%	33%	60%	40%
Quintile 5 (most deprived)	2,875	39%	26%	35%	58%	42%
Gender						
Male	5,976	38%	28%	34%	55%	45%
Female	6,748	39%	27%	34%	62%	38%
Ethnicity						
White	9,154	38%	27%	35%	59%	41%
Black & minority ethnicities	3,570	38%	28%	34%	58%	42%
Number of primary care contacts in year preceding diagnosis						
0-4	2,913	37%	29%	34%	50%	50%
5-9	3,587	39%	27%	34%	57%	43%
10-14	2,373	40%	27%	33%	60%	40%

For peer review only - http://bmjopen.bmj.com/site/about/guidelines.xhtml

For peer review only

Sample for SMI admissions	Person-years overall	Care plans: % of row person-years with current CP	Care plans: % of row person-years with expired CP	Care plans: % of row person-years with no CP	Annual reviews: % of row person-years with current AR	Annual reviews: % of row person-years with no current AR
15-19	1,422	39%	25%	36%	63%	37%
>=20	2,429	38%	26%	36%	68%	32%
Number of hospital admissions in year preceding diagnosis						
0	7,723	37%	28%	35%	57%	43%
1	2,899	41%	27%	32%	60%	40%
2	1,192	42%	25%	33%	64%	36%
3	910	38%	26%	36%	62%	38%
Number of Charlson Index comorbidities at time of diagnosis						
0	7,970	39%	28%	33%	56%	44%
1	3,472	40%	27%	33%	62%	38%
2	849	33%	22%	45%	66%	34%
3 or more	432	31%	23%	46%	68%	32%
Comorbid depression at time of diagnosis						
History of depression	8,641	40%	27%	33%	60%	40%
No recorded history of depression	4,083	37%	27%	36%	56%	44%
Smoking status						
Current or ex-smoker	9,823	39%	27%	34%	59%	41%
No recorded history of smoking	2,901	39%	27%	34%	58%	42%
SMI diagnostic group						
Schizophrenia & other psychoses	6,394	39%	28%	33%	61%	39%
Bipolar disorder & affective psychoses	5,811	39%	28%	33%	61%	39%
Both	582	44%	30%	26%	63%	37%
Financial year of diagnosis						

For peer review only

Sample for SMI admissions	Person-years overall	Care plans			Annual reviews	
		% of row person-years with current CP	% of row person-years with expired CP	% of row person-years with no CP	% of row person-years with current AR	% of row person-years with no current AR
2006/07	2,752	38%	39%	23%	65%	35%
2007/08	2,077	40%	34%	26%	64%	36%
2008/09	2,006	38%	32%	30%	62%	38%
2009/10	1,818	36%	29%	35%	59%	41%
2010/11	1,454	42%	21%	37%	60%	40%
2011/12	1,402	37%	12%	51%	50%	50%
2012/13	901	40%	3%	57%	42%	58%
2013/14	314	40%	0	60%	28%	72%

For peer review only

Supplementary Table 5: Distribution of person-years observed across patient characteristics in sample for analysis of ACSC admissions

Sample for ACSC admissions	Person-years overall	Care plans			Annual reviews	
		% of row person-years with current CP	% of row person-years with expired CP	% of row person-years with no CP	% of row person-years with current AR	% of row person-years with no current AR
Sample overall	13,203	40%	27%	33%	59%	41%
Age at diagnosis						
18-35	4,111	41%	27%	32%	52%	48%
36-45	2,990	40%	29%	31%	57%	43%
46-55	2,215	41%	29%	31%	65%	35%
56-65	1,618	41%	28%	31%	68%	32%
>65	2,269	37%	23%	40%	63%	37%
Index of multiple deprivation						
Quintile 1 (least deprived)	2,364	41%	27%	32%	61%	39%
Quintile 2	2,500	41%	27%	32%	61%	39%
Quintile 3	2,408	37%	29%	34%	56%	44%
Quintile 4	2,979	42%	26%	32%	60%	40%
Quintile 5 (most deprived)	2,952	40%	26%	34%	58%	42%
Gender						
Male	6,305	40%	27%	33%	56%	44%
Female	6,898	40%	27%	33%	62%	38%
Ethnicity						
White	9,527	40%	27%	33%	60%	40%
Black & minority ethnicities	3,676	39%	27%	34%	57%	43%
Number of primary care contacts in year preceding diagnosis						
0-4	3,209	38%	29%	33%	51%	49%
5-9	3,746	41%	27%	32%	58%	42%
10-14	2,415	41%	27%	32%	61%	39%

For peer review only

15-19	1,447	40%	25%	35%	63%	27%
>=20	2,386	39%	27%	34%	67%	33%
Number of hospital admissions in year preceding diagnosis						
0	8,010	38%	28%	34%	58%	42%
1	3,071	43%	27%	30%	60%	40%
2	1,221	44%	25%	31%	63%	37%
3	901	41%	26%	33%	63%	37%
Number of Charlson Index comorbidities at time of diagnosis						
0	8,502	40%	28%	32%	57%	43%
1	3,534	41%	27%	32%	62%	38%
2	778	35%	22%	43%	66%	34%
3 or more	389	30%	23%	47%	68%	32%
Comorbid depression at time of diagnosis						
History of depression	8,860	41%	27%	32%	60%	40%
No recorded history of depression	4,343	38%	28%	34%	57%	43%
Smoking status						
Current or ex-smoker	10,118	40%	27%	33%	60%	40%
No recorded history of smoking	3,085	40%	28%	32%	58%	42%
SMI diagnostic group						
Schizophrenia & other psychoses	6,687	40%	26%	34%	57%	43%
Bipolar disorder & affective psychoses	5,820	40%	28%	32%	61%	39%
Both	696	44%	32%	24%	66%	34%
Financial year of diagnosis						
2006/07	2,884	39%	39%	21%	65%	35%
2007/08	2,151	42%	34%	24%	65%	35%
2008/09	2,033	40%	31%	29%	63%	37%
2009/10	1,936	38%	30%	32%	60%	40%
2010/11	1,511	42%	22%	36%	60%	40%

For peer review only - http://bmjopen.bmj.com/site/about/guidelines.xhtml

2011/12	1,438	38%	12%	50%	50%	50%
2012/13	932	41%	2%	56%	43%	57%
2013/14	319	40%	0	60%	29%	71%

For peer review only

For peer review only - http://bmjopen.bmj.com/site/about/guidelines.xhtml

Supplementary Table 6: Prevalence of Charlson Index comorbidities at time of SMI diagnosis

Comorbidities at time of SMI diagnosis	Proportion of sample (%)
Cancer	7.31
Cerebrovascular disease	5.08
COPD/ Asthma	19.72
CHD	2.12
Dementia	3.06
Diabetes	7.37
Hemiplegia	0.60
Liver disease	0.78
Myocardial infarction	2.68
Peptic ulcer	2.54
Peripheral vascular disease	1.96
Renal disease	7.60
Rheumatological disease	2.56

For peer review only

Supplementary Table 7: Sensitivity tests with A&E presentation as the outcome

Model specifications	Current AR		Current CP		Expired CP	
	HR	(95% CI)	HR	(95% CI)	HR	(95% CI)
Main analysis	0.96	(0.86-1.08)	0.87*	(0.77-0.98)	0.81*	(0.67-0.97)
AR using QOF codes only (limited to 2010/11)	0.92	(0.74-1.15)	0.88	(0.68-1.15)	0.64*	(0.42-0.99)
CP and AR current for 15 months	0.93	(0.83-1.05)	0.87*	(0.77-0.98)	0.85	(0.69-1.04)
Number of Charlson index comorbidities varies over time	0.96	(0.85-1.07)	0.87*	(0.77-0.98)	0.81*	(0.67-0.98)
Observed practice characteristics and cluster-adjusted SEs	0.95	(0.86-1.05)	0.89*	(0.80-0.99)	0.82*	(0.69-0.96)
Fixed practice effects and cluster-adjusted SEs	0.96	(0.86-1.06)	0.87*	(0.77-0.97)	0.83*	(0.70-0.98)

*p<0.05, **p<0.01, ***p<0.001

CP: Care plan (base: never had a care plan)

AR: Annual review (base: no annual review in last 12 months)

Hazard ratios represent the relative hazard of a patient with a care indicator compared with an otherwise identical patient with the base level care indicator.

For peer review only

Supplementary Table 8: Sensitivity tests with SMI admission as the outcome

Model specifications	Current AR		Current CP		Expired CP	
	HR	(95% CI)	HR	(95% CI)	HR	(95% CI)
Main analysis	1.06	(0.84-1.35)	1.02	(0.81-1.28)	0.90	(0.63-1.29)
AR using original codes only (limited to 2010/11)	1.03	(0.72-1.47)	1.01	(0.69-1.47)	0.84	(0.46-1.53)
CP and AR current for 15 months	1.06	(0.82-1.36)	1.00	(0.79-1.27)	1.00	(0.68-1.48)
Number of Charlson index comorbidities varies over time	1.08	(0.86-1.37)	1.00	(0.80-1.26)	0.89	(0.62-1.27)
Observed practice characteristics and cluster-adjusted SEs	1.08	(0.87-1.34)	1.05	(0.84-1.31)	0.98	(0.72-1.32)
Fixed practice effects and cluster-adjusted SEs	1.11	(0.88-1.40)	1.06	(0.85-1.33)	1.04	(0.75-1.43)

*p<0.05, **p<0.01, ***p<0.001

CP: Care plan (base: never had a care plan)

AR: Annual review (base: no annual review in last 12 months)

Hazard ratios represent the relative hazard of a patient with a care indicator compared with an otherwise identical patient with the base level care indicator.

For peer review only

For peer review only - http://bmjopen.bmj.com/site/about/guidelines.xhtml

Supplementary Table 9: Sensitivity tests with ACSC admission as the outcome

Model specifications	Current AR		Current CP		Expired CP	
	HR	(95% CI)	HR	(95% CI)	HR	(95% CI)
Main analysis	1.13	(0.87-1.47)	0.77*	(0.60-0.99)	0.88	(0.60-1.28)
AR using original codes only (limited to 2010/11)	0.85	(0.57-1.28)	1.07	(0.67-1.70)	0.84	(0.44-1.58)
CP and AR current for 15 months	1.08	(0.83-1.41)	0.79	(0.61-1.02)	0.90	(0.62-1.30)
Number of Charlson index comorbidities varies over time	1.08	(0.83-1.42)	0.79	(0.61-1.02)	0.92	(0.62-1.36)
Observed practice characteristics and cluster-adjusted SEs	1.05	(0.84-1.32)	0.79*	(0.63-0.99)	0.88	(0.63-1.22)
Fixed practice effects and cluster-adjusted SEs	1.03	(0.81-1.30)	0.82	(0.64-1.05)	0.95	(0.66-1.35)

*p<0.05, **p<0.01, ***p<0.001

CP: Care plan (base: never had a care plan)

AR: Annual review (base: no annual review in last 12 months)

Hazard ratios represent the relative hazard of a patient with a care indicator compared with an otherwise identical patient with the base level care indicator.

For peer review only - http://bmjopen.bmj.com/site/about/guidelines.xhtml

The RECORD statement – checklist of items, extended from the STROBE statement, that should be reported in observational studies using routinely collected health data.

	Item No.	STROBE items	Location in manuscript where items are reported	RECORD items	Location in manuscript where items are reported
**Title and abstract**					
	1	(a) Indicate the study's design with a commonly used term in the title or the abstract (b) Provide in the abstract an informative and balanced summary of what was done and what was found		RECORD 1.1: The type of data used should be specified in the title or abstract. When possible, the name of the databases used should be included.<br><br>RECORD 1.2: If applicable, the geographic region and timeframe within which the study took place should be reported in the title or abstract.<br><br>RECORD 1.3: If linkage between databases was conducted for the study, this should be clearly stated in the title or abstract.	P1
**Introduction**					
Background rationale	2	Explain the scientific background and rationale for the investigation being reported			P5-6
Objectives	3	State specific objectives, including any prespecified hypotheses			P6
**Methods**					
Study Design	4	Present key elements of study design early in the paper			P7
Setting	5	Describe the setting, locations, and relevant dates, including periods of recruitment, exposure, follow-up, and data collection			P7
Participants	6	*(a) Cohort study* - Give the		RECORD 6.1: The methods of study	

		eligibility criteria, and the sources and methods of selection of participants. Describe methods of follow-up<br>*Case-control study* - Give the eligibility criteria, and the sources and methods of case ascertainment and control selection. Give the rationale for the choice of cases and controls<br>*Cross-sectional study* - Give the eligibility criteria, and the sources and methods of selection of participants<br><br>*(b) Cohort study* - For matched studies, give matching criteria and number of exposed and unexposed<br>*Case-control study* - For matched studies, give matching criteria and the number of controls per case		population selection (such as codes or algorithms used to identify subjects) should be listed in detail. If this is not possible, an explanation should be provided.<br><br>RECORD 6.2: Any validation studies of the codes or algorithms used to select the population should be referenced. If validation was conducted for this study and not published elsewhere, detailed methods and results should be provided.<br><br>RECORD 6.3: If the study involved linkage of databases, consider use of a flow diagram or other graphical display to demonstrate the data linkage process, including the number of individuals with linked data at each stage.	P7
Variables	7	Clearly define all outcomes, exposures, predictors, potential confounders, and effect modifiers. Give diagnostic criteria, if applicable.		RECORD 7.1: A complete list of codes and algorithms used to classify exposures, outcomes, confounders, and effect modifiers should be provided. If these cannot be reported, an explanation should be provided.	Supp. tables 1&2
Data sources/ measurement	8	For each variable of interest, give sources of data and details of methods of assessment (measurement).<br>Describe comparability of assessment methods if there is more than one group			P8-10
Bias	9	Describe any efforts to address potential sources of bias			P8-11

Study size	10	Explain how the study size was arrived at			P7-8, 12
Quantitative variables	11	Explain how quantitative variables were handled in the analyses. If applicable, describe which groupings were chosen, and why			P10, Tables 1&2
Statistical methods	12	(a) Describe all statistical methods, including those used to control for confounding<br>(b) Describe any methods used to examine subgroups and interactions<br>(c) Explain how missing data were addressed<br>(d) *Cohort study* - If applicable, explain how loss to follow-up was addressed<br>*Case-control study* - If applicable, explain how matching of cases and controls was addressed<br>*Cross-sectional study* - If applicable, describe analytical methods taking account of sampling strategy<br>(e) Describe any sensitivity analyses			P7-8,10-12
Data access and cleaning methods		..		RECORD 12.1: Authors should describe the extent to which the investigators had access to the database population used to create the study population.<br><br>RECORD 12.2: Authors should provide information on the data cleaning methods used in the study.	P7-8
Linkage		..		RECORD 12.3: State whether the	

For peer review only - http://bmjopen.bmj.com/site/about/guidelines.xhtml

				study included person-level, institutional-level, or other data linkage across two or more databases. The methods of linkage and methods of linkage quality evaluation should be provided.	P7
**Results**					
Participants	13	(a) Report the numbers of individuals at each stage of the study (*e.g.*, numbers potentially eligible, examined for eligibility, confirmed eligible, included in the study, completing follow-up, and analysed)<br>(b) Give reasons for non-participation at each stage.<br>(c) Consider use of a flow diagram		RECORD 13.1: Describe in detail the selection of the persons included in the study (*i.e.,* study population selection) including filtering based on data quality, data availability and linkage. The selection of included persons can be described in the text and/or by means of the study flow diagram.	P7-8, 12-13,<br>Table 1
Descriptive data	14	(a) Give characteristics of study participants (*e.g.*, demographic, clinical, social) and information on exposures and potential confounders<br>(b) Indicate the number of participants with missing data for each variable of interest<br>(c) *Cohort study* - summarise follow-up time (*e.g.*, average and total amount)			Table 1
Outcome data	15	*Cohort study* - Report numbers of outcome events or summary measures over time<br>*Case-control study* - Report numbers in each exposure category, or summary measures of exposure<br>*Cross-sectional study* - Report numbers of outcome events or			P16, Table 2

For peer review only - http://bmjopen.bmj.com/site/about/guidelines.xhtml

		summary measures			
Main results	16	(a) Give unadjusted estimates and, if applicable, confounder-adjusted estimates and their precision (e.g., 95% confidence interval). Make clear which confounders were adjusted for and why they were included<br>(b) Report category boundaries when continuous variables were categorized<br>(c) If relevant, consider translating estimates of relative risk into absolute risk for a meaningful time period			P20, Table 3
Other analyses	17	Report other analyses done—e.g., analyses of subgroups and interactions, and sensitivity analyses			Supp. Tables 7-9
**Discussion**					
Key results	18	Summarise key results with reference to study objectives			P21
Limitations	19	Discuss limitations of the study, taking into account sources of potential bias or imprecision. Discuss both direction and magnitude of any potential bias		RECORD 19.1: Discuss the implications of using data that were not created or collected to answer the specific research question(s). Include discussion of misclassification bias, unmeasured confounding, missing data, and changing eligibility over time, as they pertain to the study being reported.	P22-23
Interpretation	20	Give a cautious overall interpretation of results considering objectives, limitations, multiplicity of analyses, results from similar studies, and other relevant evidence			P21-25

For peer review only - http://bmjopen.bmj.com/site/about/guidelines.xhtml

Generalisability	21	Discuss the generalisability (external validity) of the study results			P22
**Other Information**					
Funding	22	Give the source of funding and the role of the funders for the present study and, if applicable, for the original study on which the present article is based			P4
Accessibility of protocol, raw data, and programming code		..		RECORD 22.1: Authors should provide information on how to access any supplemental information such as the study protocol, raw data, or programming code.	P27

*Reference: Benchimol EI, Smeeth L, Guttmann A, Harron K, Moher D, Petersen I, Sørensen HT, von Elm E, Langan SM, the RECORD Working Committee. The REporting of studies Conducted using Observational Routinely-collected health Data (RECORD) Statement. *PLoS Medicine* 2015; in press.

*Checklist is protected under Creative Commons Attribution (CC BY) license.
